# Resistive Switching Random-Access Memory (RRAM): Applications and Requirements for Memory and Computing

**DOI:** 10.1021/acs.chemrev.4c00845

**Published:** 2025-05-02

**Authors:** Daniele Ielmini, Giacomo Pedretti

**Affiliations:** † Dipartimento di Elettronica, Informazione e Bioingegneria (DEIB), Politecnico di Milano and IUNET, piazza L. da Vinci 32, 20133, Milano, Italy; ‡ Artificial Intelligence Research Lab, Hewlett-Packard Labs, 820 N McCarthy Blvd, Milpitas, California 95035, United States

## Abstract

In the information age, novel hardware solutions are urgently needed to efficiently store and process increasing amounts of data. In this scenario, memory devices must evolve significantly to provide the necessary bit capacity, performance, and energy efficiency needed in computation. In particular, novel computing paradigms have emerged to minimize data movement, which is known to contribute the largest amount of energy consumption in conventional computing systems based on the von Neumann architecture. In-memory computing (IMC) provides a means to compute within data with minimum data movement and excellent energy efficiency and performance. To meet these goals, resistive-switching random-access memory (RRAM) appears to be an ideal candidate thanks to its excellent scalability and nonvolatile storage. However, circuit implementations of modern artificial intelligence (AI) models require highly specialized device properties that need careful RRAM device engineering. This work addresses the RRAM concept from materials, device, circuit, and application viewpoints, focusing on the physical device properties and the requirements for storage and computing applications. Memory applications, such as embedded nonvolatile memory (eNVM) in novel microcontroller units (MCUs) and storage class memory (SCM), are highlighted. Applications in IMC, such as hardware accelerators of neural networks, data query, and algebra functions, are illustrated by referring to the reported demonstrators with RRAM technology, evidencing the remaining challenges for the development of a low-power, sustainable AI.

## Graphical abstract

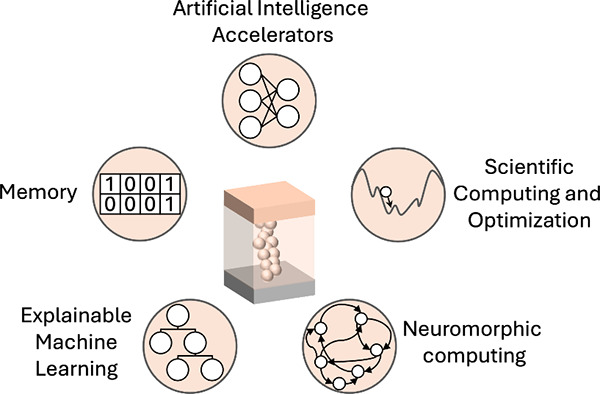

## 1 Introduction

According to the von Neumann architecture, a computer consists of two essential parts, namely, the central processing unit (CPU) and the memory. The latter must support both instructions and data for the computation, which is executed in the CPU. With the massive increase of data and the widespread use of artificial intelligence (AI) in our modern digital society, memory and computing demand have seen an exponential increase which dictates the introduction of novel memory technologies and computing paradigms. In particular, there is a need to introduce a novel memory concept that can provide a large density combined with a high performance in terms of data access time, thus unifying the properties of storage and memory modules.^1^ In addition, for modern AI and machine learning applications, the processing time and energy consumption become limited by the data movement between the memory and the CPU.^2^ Overcoming this fundamental gap of performance requires the introduction of novel computing paradigms, such as in-memory computing (IMC), capable of moving a large portion of the computation within the memory, thus alleviating the memory bottleneck.^3,4^

In this scenario, memory technologies acquire paramount importance, as they must provide a broad scope of properties, including nonvolatile storage, low voltage/current operation, high scaling capability, compatibility with the CMOS process flow, and integration in the back end of the line (BEOL). The last 25 years have seen the introduction of several emerging nonvolatile memory (NVM) technologies, such as resistive switching random-access memory (RRAM), phase change memory (PCM), magnetic random-access memory (MRAM), and ferroelectric random-access memory (FeRAM).^1,5^ Historically, these technologies have been known for a relatively long time due to pioneering research works on a variety of materials and devices, such as oxides^6^ or chalcogenides.^7^ Currently, these memory concepts can hardly replace existing established technologies, such as static random-access memory (SRAM), dynamic random-access memory (DRAM), and nonvolatile flash memory, due to insufficient performance and excessive cost. On the other hand, emerging memories can provide a unique solution for embedded memories, where a high-capacity memory needs to be integrated into the same chip of a computing system, such as a microcontroller unit (MCU) for edge computing. Emerging memories also provide an improved radiation hardness compared to conventional CMOS-based memories, such as Flash memories. In particular, RRAM has been shown to have excellent radiation hardness, which is crucial for radiation-tolerant systems in spaceborne applications.^8,9^ Also, emerging memory devices combining nonvolatile storage and high density are a suitable platform for IMC circuits for AI applications.^10^

Among the emerging NVM technologies, RRAM displays a simple device structure and fabrication process that are amenable to crossbar array (CBA) architecture and 3D integration to achieve extremely high density. The nonvolatile switching behavior ensures good retention even at elevated temperatures, while program/erase cycling shows strong endurance, making RRAM an ideal solution for embedded nonvolatile memory (eNVM). The properties of RRAM devices match well with the requirements of several computing applications, such as nonvolatile behavior, multilevel operation, good scaling capability, and high linearity. RRAM can also offer unconventional properties such as stochastic phenomena and short-term memory effects, which are useful in selected computing applications. All of these properties can be tuned and optimized by careful materials and device engineering as well as circuit design. Usually, computing application requirements are met by a detailed design/technology co-optimization (DTCO), where the most convenient solution is provided by a specific set of materials, stack, process steps, device geometry, and circuit design.

Overall, thanks to its simple structure and flexible concept, RRAM appears as a strong candidate for advanced memory technology and IMC. However, many challenges still need to be addressed, including the optimization of the programming precision, linearity, and endurance, as well as the feasibility and energy efficiency of the overall RRAM computing system, which also includes ancillary circuits such as the analog-digital converters, the programming periphery circuits, the select/unselect decoders, and the digital controller. To solve these fundamental challenges, a cross-disciplinary research approach is essential, where materials engineering, device technology, circuit design, conceptual architecture, and final application, including its requirements, are fully understood and carefully monitored.

To meet these goals, the purpose of this work is to provide a comprehensive overview of RRAM from materials, devices, circuits, systems, and applications viewpoints. The review is organized as follows. Section 2 provides an overview of RRAM devices including device structure, characteristics, and operation. Section 3 describes the RRAM cell and array structure for memory applications, including a summary of presented demonstrators in the literature. Section 4 presents RRAM circuits for computing primitives, such as matrix-vector multiplication (MVM) and inverse matrix calculation. Section 5 addresses RRAM-based computing applications, focusing on various AI, neural networks, and other popular machine learning tasks and highlighting the specific requirements which are essential for each computing task. Section 6 provides a conclusion and a perspective on the open research challenges.

## 2 RRAM Devices

Resistive switching random-access memory (RRAM) is a memory device capable of changing its resistance upon the application of electrical pulses.^11–13^ Most typically, the RRAM structure consists of a metal–insulator–metal (MIM) stack, where the insulating layer can be modified by the presence and growth of a conductive filament (CF) shunting the two metal electrodes. This is shown in Figure 1a, indicating the MIM where a CF connects the metal electrodes across the insulating layer. Modification of the CF leads to a resistance change of the MIM structure, which is thus responsible for the resistance switching effect. The CF is generally first introduced in the MIM structure by an electrical forming operation, also known as electroforming, which consists of a controlled voltage-induced breakdown operation of the insulating layer.^14^ Then, the CF can be activated or deactivated by generation of a depleted gap across the filament as shown in Figure 1b. Figure 1c shows the typical current–voltage (*I*–*V*) curve for a RRAM device, displaying the set transition for the switching from high to low resistance and the reset transition for the switching from high to low resistance. The type of switching displayed in Figure 1c is the *unipolar* switching of RRAM, where the set and reset transition can take place at the same voltage polarity.^15,16^ Most relevant for the memory and computing applications of RRAM is the *bipolar* characteristic in Figure 1d, where the set and reset transitions take place at opposite polarities.^17^ The set transition generally shows a steep slope in the *I*–*V* curve from high to low resistance, which is attributed to the negative differential resistance (NDR) due to CF formation and the consequent growth of a low resistance path across the oxide. The reset transition instead shows a more gradual, continuous change in the *I*–*V* curve, as the CF is gradually disconnected or retracted in response to the electric field.^14^

**Figure 1:**
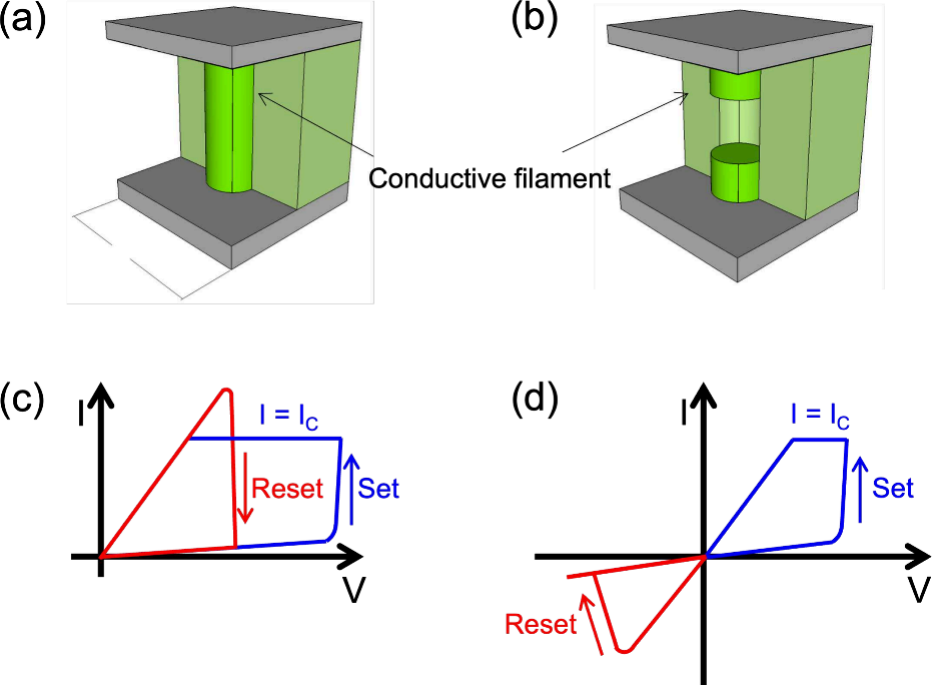
Sketch of RRAM and its switching characteristics. (a, b) Sketch of the RRAM device, including the conductive filament (CF). (c) Schematic *I*–*V* curve for a unipolar switching RRAM device. (d) Schematic *I*–*V* curve for a bipolar switching RRAM device. Reproduced from ref 14. Copyright 2016 IOP Publishing Ltd. with Creative Commons Attribution 3.0 license https://creativecommons.org/licenses/by/3.0/.

In the case of unipolar switching, although set/reset operations occur at the same polarity, they differ by the current condition, in that the set operation requires a limitation in current known as the compliance current (CC) to prevent the destructive breakdown of the device. The CC is generally adopted for the set process of bipolar switching as well, to minimize degradation and enable tight control of the final resistive state. The two stable states of RRAM are known as the high resistance state (HRS) and the low resistance state (LRS), which are obtained after the reset and set transition, respectively.

Early reports about resistive switching (RS) have been published in the 1960s within studies of the reversible breakdown phenomena in thin metal oxides, such as SiO_*x*_, Al_2_O_3_, Ta_2_O_5_, ZrO_2_, and TiO_2_.^6^ In general, these layers displayed an NDR effect which was explained by a space-charge-limited current (SCLC) originating from the trapping of electrons in localized states.^18^ Studies in niobium oxide (Nb_2_O_5_) layers demonstrated bistable RS between two stable states.^19^ Figure 2 shows one of the first reported *I*–*V* curves for RS, where the LRS (a) is first subject to a reset transition to the HRS at negative voltage (b), followed by a set transition back to the LRS at positive voltage (c).^20^ Both the HRS and LRS were found to be stable, thus supporting the possibility of conceiving a nonvolatile memory (NVM) from a RRAM device.

**Figure 2:**
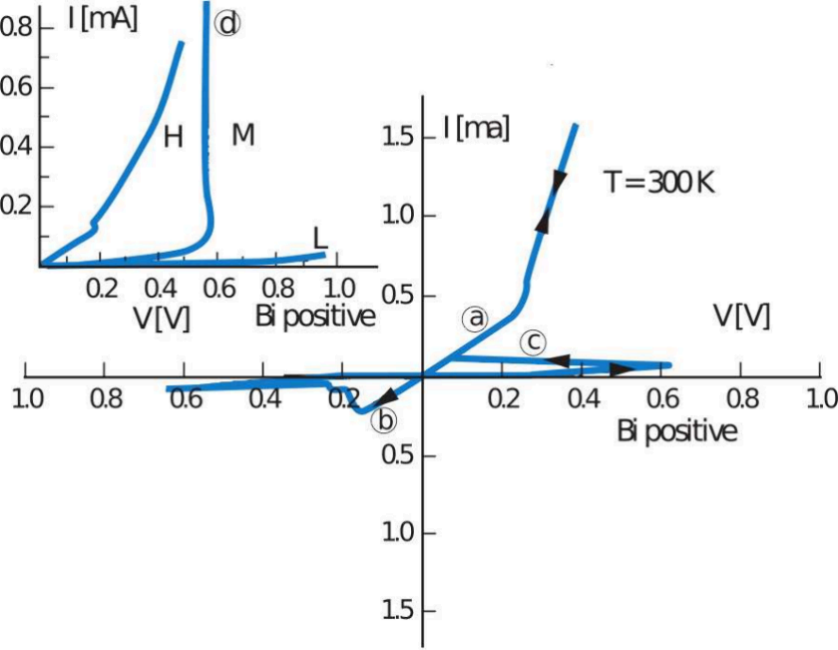
Measured *I*–*V* curves of a MIM stack with a Nb_2_O_5_ insulating layer. Resistance switching is demonstrated where the application of a negative voltage (b) causes a reset transition from LRS (a) to HRS, and the application of a positive voltage (c) causes a set transition from HRS to LRS. Reproduced with permission from ref 20. Copyright 1965 American Institute of Physics.

Those early studies were mostly aimed at elucidating the fundamental transport properties of insulating layers, such as transition metal oxide. However, in the early 2000s interest in RS phenomena rose significantly for studying NVM applications. Similar to phase change memory (PCM), ferroelectric random access memory (FeRAM), and magnetic random access memory (MRAM), RRAM devices were extensively studied with the specific purpose of developing a new class of memory technology. In particular, research on these emerging memory concepts was aimed at assessing the scalability, density, performance, energy consumption, reliability, and cost of the technology, to be compared to conventional memories of complementary metal-oxide-semiconductor (CMOS) technology, such as static random access memory (SRAM), dynamic random access memory (DRAM) and Flash NVM. Given the excellent combination of speed, reliability, low voltage operation, and endurance, RRAM devices were even targeted as potential ‘universal’ memory, capable of satisfying the requirements of all major device technologies, from SRAM to Flash.^13^

### 2.1 Unipolar Switching RRAM

Unipolar switching in NiO-based RRAM devices first attracted interest as a high-density NVM technology. Figure 3 shows the measured *I*–*V* curve for polycrystalline NiO films deposited on Pt/Ti/SiO_2_/Si substrates, indicating unipolar switching for both positive and negative applied voltages, also referred to as nonpolar switching.^15^ Two types of switching are shown in Figure 3, namely, nonvolatile, or memory, switching (Figure 3a) and volatile, or threshold, switching (Figure 3b), where the set transition results in an unstable LRS, which spontaneously switches back to HRS within a short retention time. Memory and threshold switching in NiO were found for different ratios of Ni and O concentrations in the NiO film, which were Ni/O = 1.05 and 0.95 in Figure 3a and b, respectively. Memory switching took place under the same polarity by applying a proper k during the set transition. On the other hand, threshold switching leads to a transition from HRS to LRS with CC of 3 mA; however, the HRS was recovered as the voltage decreased below a characteristic holding voltage VH. Although not useful for NVM technology, threshold switching has a significant role in several applications for both storage, *e.g*. select devices in CBAs,^21^ and computing, such as short-term memory^22^ and oscillating circuits.^23^ Unipolar memory switching with RRAM was explored given the simplicity of the circuit integration, where not only a field-effect transistor (FET)^16^ but also unipolar diodes or bipolar junction transistors can be adopted for high-density NVM CBAs.^24^ Metal-oxide *p*–*n* diodes suitable for integration in the back-end of the line (BEOL) of the CMOS process flow were demonstrated as RRAM selectors, thus enabling 3D stackable high-density CBAs.^25^

**Figure 3:**
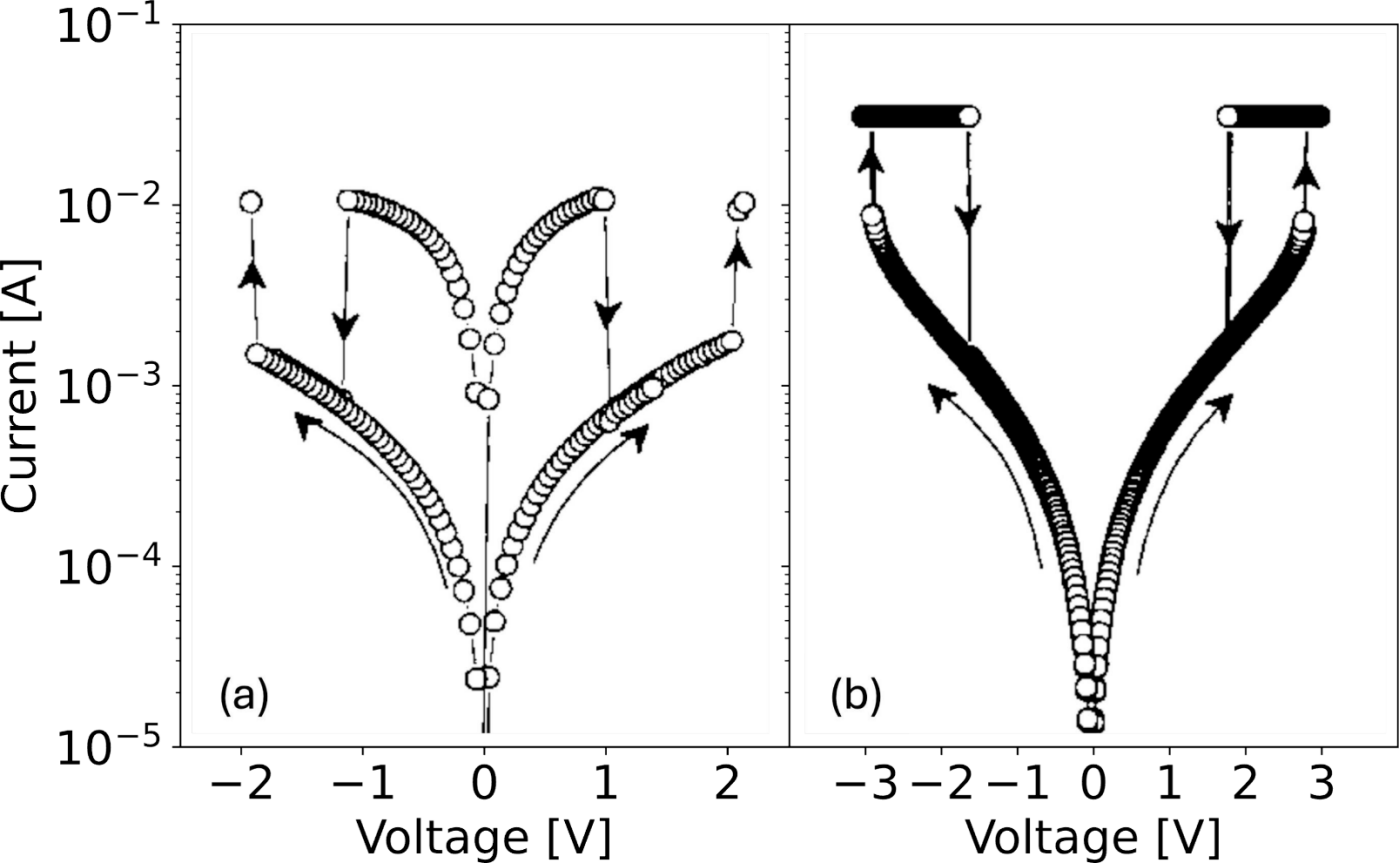
Measured *I*–*V* curves for Pt/NiO/Pt RRAM devices with memory switching (a) and threshold switching (b) under either polarity, thus demonstrating nonpolar unipolar switching. The different behaviors are due to different Ni/O ratios in the switching layer, namely, 1.05 and 0.95 in (a) and (b), respectively. Reproduced with permission from ref 15. Copyright 2004 AIP Publishing.

### 2.2 Bipolar Switching RRAM

While advantageous from an integration viewpoint, unipolar switching RRAM devices showed poor reproducibility of switching mainly due to the lack of control of the CF size and resistance in the LRS. Unipolar switching is mainly explained by thermochemical oxidation and diffusion of the material locally at the CF as a result of Joule heating, which lacks directionality.^26^ Bipolar switching RRAM then attracted interest because of the improved ability to control the ionic migration responsible for CF growth and disconnection. Contrary to thermally induced oxidation and diffusion, field-induced migration can be directed toward either electrode side, thus enabling the controllable modulation of CF resistance.^27^ After the seminal works of the 1960s,^19^ studies on bipolar switching of metal oxides were revived by covering perovskite materials, such as SrZrO_3_,^28^ and binary metal oxides such as TiO_2_ ^29^ and HfO_2_.^17,30,31^ Among the latter materials, HfO_2_ raised considerable interest, mostly thanks to the relevance of this material as a high-k gate dielectric for the logic CMOS technology.^32,33^

Figure 4a shows the measured *I*–*V* curves of HfO_2_-based RRAM, indicating bipolar switching with controllable LRS resistance via the CC.^17^ The bipolar switching effect can be understood by the directional migration of ionic species, such as oxygen vacancies responsible for the higher local conductivity in the CF.^27^ During reset, field- and temperature-induced ionic migration cause the opening of a depleted gap across the CF, thus bringing the device into an HRS. By increasing the time and/or the voltage of the reset operation, the gap length increases its length, thus resulting in a higher resistance and enabling tight control of the HRS resistance.^35^ During the set transition, the applied field causes the migration of ions in the opposite direction, thus replenishing the previously opened gap and restoring the LRS conductance.^27^ The CC plays a key role during the set transition by limiting the final resistance of the LRS to the value *R* = *V*_*C*_/*I*_*C*_, where *V*_*C*_ is a critical voltage, characteristic of the microscopic ion-migration process, and *I*_*C*_ is the CC.^33,36^ The critical voltage *V*_*C*_ represents the voltage value for the acceleration of the CF growth by ionic migration at the time scale characteristic of the experiment, e.g. about 1 s for a typical quasi-static experiment. Experimental results indicate that this voltage increases at decreasing times during the set transition.^37^ Due to the weak dependence of *V*_*C*_ among different RRAM materials, the LRS resistance was found to follow a universal behavior when plotted as a function of the CC.^33^ Note that ionic migration is a directional process guided by the field, thus supporting the repeatability of the set-reset process at the basis of cycling endurance. Cycle-to-cycle variability is also strongly reduced compared to unipolar switching RRAM,^38^ as the same defects are consistently reused during bipolar set/reset processes, thus mitigating defect-number variation.^39^ HfO_2_-based RRAM also showed excellent switching speed^30^ and scaling in the 10 nm range,^40^ thus supporting this materials system as a promising solution for scalable RRAM.

**Figure 4:**
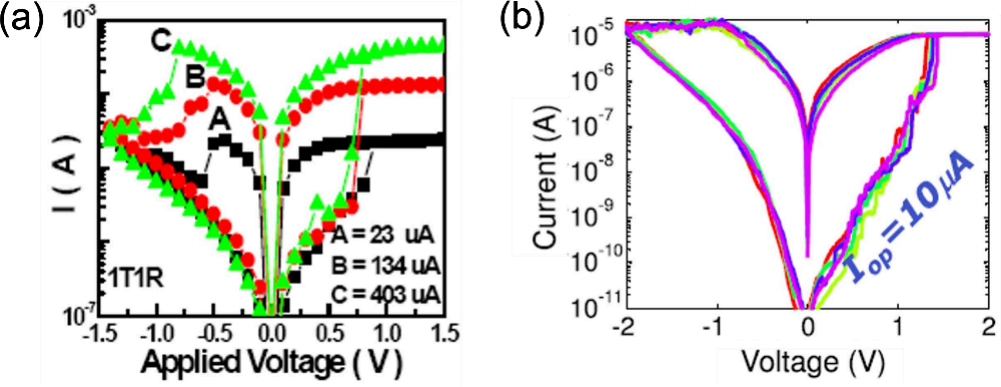
Measured *I*–*V* curves for bipolar switching RRAM devices, namely Ti/HfO_2_/Pt RRAM^17^ (a) and Cu/AlO_*x*_ conductive-bridge random-access memory (CBRAM) device (b).^34^ Panel (a) is adapted with permission from ref 17. Copyright 2008 IEEE. Panel (b) is adapted with permission from ref 34. Copyright 2013 IEEE.

### 2.3 RRAM Stack Optimization

Despite the outstanding performance of HfO_2_-based RRAM, it was soon realized that RRAM optimization requires an overarching engineering effort aimed at the whole RRAM stack, including both metal oxide and metal electrodes, in terms of composition profile, material structure, and interfaces. Several RRAM stacks were then reported with the objective of optimizing the device behavior from various perspectives.

Figure 4b shows the *I*–*V* curve for a conductive-bridge random-access memory (CBRAM), also known as the electrochemical metallization (ECM) device.^34^ In CBRAM, the top electrode material is replaced by an active metal, such as Cu,^34,41–43^ Ag,^44^ or CuTe.^45^ Application of a positive voltage to the top electrode causes the field-induced oxidation and migration of electrode cations across the insulating layer, also known as the electrolyte. The latter consists of a chalcogenide layer, such as GeSe^41^ or GeS_2_,^44^ or an oxide layer, such as Al_2_O_3_,^34^ ZrO_*x*_,^42^ SiO_2_ ^43^ or GdO_*x*_.^45^ Compared to conventional oxide-based RRAM devices, CBRAMs display a larger resistance window,^46^ in a range of 10^4^ compared to about 10^2^ for the case of metal-oxide RRAM. The higher resistance window can be explained by the higher ionic mobility of Cu and Ag in CBRAM compared to oxygen vacancies and enables the design and integration in high-density memory arrays.^47^ The relatively high ionic mobility of Cu and Ag can be challenging due to thermally induced diffusion during the BEOL process at 400 °C. Process-induced Cu diffusion was reduced by diffusion barriers such as TiW inserted between the electrolyte and the Cu injecting electrode without compromising the memory performance.^34^ Thanks to the large resistance window, multilevel operation over a resistance range of 6 orders of magnitude of the LRS was demonstrated by CC-control of the set transition.^48^ Due to the high mobility of the cation species, especially in the case of Ag, the CF generally displays a short retention time, which enables short-term memory and other dynamic properties that become useful in neuromorphic computing (Section 5.7).

Binary metal oxide layers also require careful design and engineering to improve the electrical performance. Figure 5a shows the transmission electron microscopy (TEM) image of the cross-section of a RRAM device with a TiN/Ti/HfO_*x*_/TiN stack.^38^ A thin Ti cap was introduced between TiN and HfO_*x*_ to enable oxygen exchange according to the following reaction:

**Figure 5:**
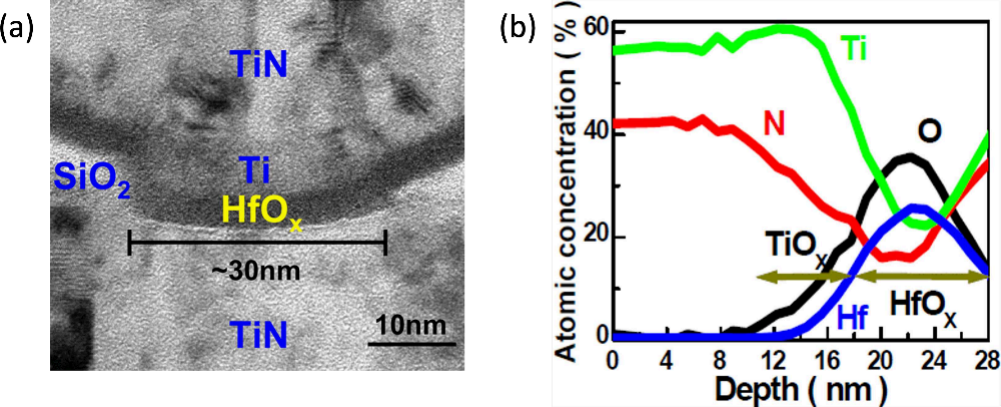
Top electrode engineering for bipolar switching RRAM devices. (a) TEM image of a TiN/Ti/HfO_*x*_/TiN stack for a bipolar switching RRAM device.^38^ (b) XPS depth profile of a TiN/Ti/HfO_2_/TiN stack indicating the presence of the OEL.^17^ Panel (a) is adapted with permission from ref 17. Copyright 2008 IEEE. Panel (b) is adapted with permission from ref 38. Copyright 2009 IEEE.

1 $$Ti+HfO_{2}↔TiO_{x}+HfO_{x}$$

thus resulting in an intermediate oxygen exchange layer (OEL) with a high concentration of oxygen vacancies. The OEL is clearly shown in Figure 5b, reporting the X-ray photoelectron spectroscopy (XPS) profile of the stack in Figure 5a and indicating a relatively wide transition region between the TiN and HfO_*x*_ layers.^17^ The generation of oxygen vacancies provides an initial reservoir of defects available for migration during forming, set, and reset operation of the RRAM device, thus supporting good performance and reliability of the device. Similar cap layers to form the OEL were adopted in several RRAM reports, with the cap consisting of Ti,^17,29^ Hf,^30^ or Ta.^49,50^

To better assess the impact of the OEL on the device performance, Figure 6a shows the measured *I*–*V* curves for a HfO_*x*_-based RRAM device under forming, set, and reset operation.^51^ The forming voltage is critical for the device, since it dictates the size of the selector and decoder transistors, which must sustain part of the applied voltage soon after the forming event. The forming voltage is directly linked to the leakage current across the pristine device, as shown in Figure 6b.^52^ Here, the leakage was increased and the forming voltage was decreased by increasing the thickness of the Ti cap layer in the RRAM stack, resulting in a more extensive O exchange and, hence, a larger concentration of oxygen vacancies.^52^ Optimizing the metal cap and thermal annealing to activate the O exchange are essential to control and minimize the forming voltage. Forming-free RRAM devices have also been developed to mitigate the forming issue.^53^

**Figure 6:**
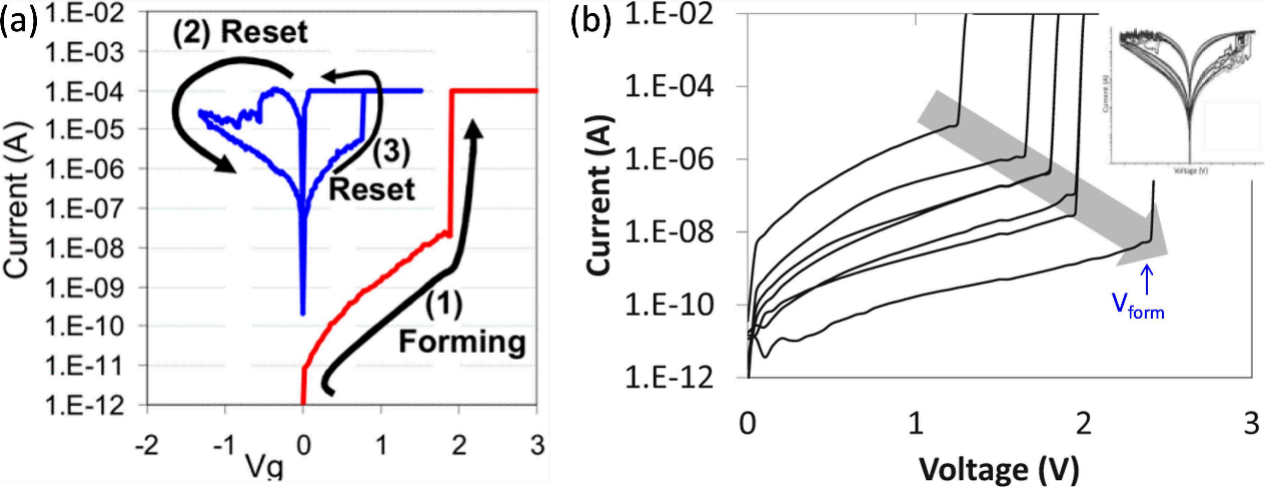
Impact of OEL on forming. (a) Measured *I*–*V* curves showing set, reset and forming characteristics for a HfO_*x*_-based RRAM.^51^ (b) Measured *I*–*V* curves showing an increase of leakage current and a decrease of forming voltage for increasing thickness of the Ti cap layer.^52^ Panel (a) is adapted with permission from ref 51. Copyright 2011 IEEE. Panel (b) is adapted with permission from ref 52. Copyright 2013 IEEE.

Stack optimization is important not only for forming but also for set operation. A key issue of both forming and set is the abrupt transition to a lower resistance, which can result in a high-voltage degradation of the select transistor, as well as in current overshoot effects causing device overprogramming and excessive reset currents.^36^ To minimize the overshoot effects, a local series resistance can be integrated close to the RRAM device to accommodate part of the applied voltage at the set/forming transition. Such close integration of the switching device and the conductive device can be achieved by bilayer structures, where one layer acts as a series resistance while the other layer acts as the proper switching layer.^49^ This is the case for the Ta_2_O_5_/TaO_*x*_ bilayer structures shown in Figure 7a, consisting of a relatively thick conductive TaO_*x*_ layer and a relatively thin switching Ta_2_O_5_ layer.^54^ Figure 7b shows the cross-sectional TEM image of the RRAM stack, including the Pd top and bottom electrodes. As shown in Figure 7a, the CF extends across only the thin switching layer, whereas the conductive layer only serves as a series resistance to prevent excessive degradation to the select transistors and overshoot effects. The integration of the series resistance within the RRAM stack enables high scalability and low parasitic capacitances. Bilayer structures based on the TiN/TaO_*x*_/HfO_2_/TiN stack, where TaO_*x*_ and HfO_2_ serve as conductive and switching layers, respectively, were recently reported to enable analog-type switching with improved control of the HRS and LRS states.^56^

**Figure 7:**
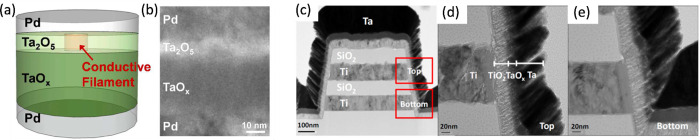
Bilayer RRAM structures. (a) Sketch and (b) cross-sectional TEM image of a Ta_2_O_5_/TaO_*x*_ bilayer, where Ta_2_O_5_ and TaO_*x*_ layers serve as the switching and the conductive layers, respectively.^54^ (c) Cross-sectional TEM of a vertical RRAM device with TiO_2_/TaO_*x*_ bilayers, with close-up images of the (d) top and (e) bottom device in the vertical structure.^55^ Panels (a) and (b) are reprinted from ref 54. Copyright 2014 ACS. Panels (c,d,e) are adapted with permission from ref 56. Copyright 2016 IEEE.

Similar RRAM structures consisting of TiO_2_/TaO_*x*_ bilayers were reported to enable analog switching in vertical RRAM devices as shown in Figure 7c,d,e.^55,57–59^ Atomic layer deposition (ALD) is generally adopted as a deposition tool to tightly control the thickness, uniformity, composition, and structure of each layer in the bilayer stack.^60^ Analog switching was optimized in bilayer stacks, such as HfO_2_/Al:TiO_2_ ^61^ and TaO_*x*_/HfO_2_,^62^ to achieve high linearity, high symmetry and high endurance, which are essential in hardware accelerators for supervised training of neural networks (see Section 5.3).

In addition to the top electrode and oxide layers, bottom electrode engineering is also essential, particularly for reliability optimization. Cycling endurance in bipolar RRAM devices was shown to be limited by the unwanted set transition occurring under negative polarity when the normal set process was expected under positive voltage.^63^ To prevent a negative set, the bottom electrode should be as chemically inert as possible. RRAM with bottom electrodes based on inert materials such as Pt,^64^ C,^65^ and Ru^66^ has been shown to display excellent retention, thanks to a reduced chemical ionization of the bottom electrode and reduced interaction with the oxide layer.

### 2.4 Nonfilamentary RRAM Devices

Although most of the RRAM implementation relies on the filamentary concept, RRAM devices based on uniform (or interface) switching were also reported. In these devices, resistance switching results from a change in the resistivity which extends uniformly across the active device area via an electrically induced change of the stack composition impacting the local conductivity. A possible physical mechanism for the uniform switching is illustrated in Figure 8, showing the density profile of oxygen vacancies in the LRS (a) and the HRS (b).^67^ First, oxygen vacancies are uniformly distributed in an OEL at the top-electrode side, thus resulting in relatively high conductivity across the RRAM oxide layer. The application of a negative voltage to the top electrode results in the migration of oxygen ions toward the OEL, thus causing the reoxidation of the OEL with a local increase of resistivity due to the formation of a Schottky barrier.^67^ The reoxidized layer is indicated as Oxide B in the figure, where reoxidation has taken place via partial depletion of oxygen from the Oxide A layer.

**Figure 8:**
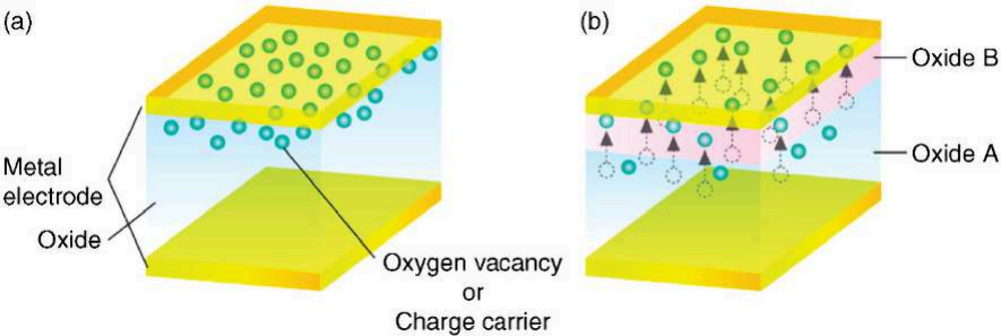
Uniform switching RRAM concept illustrating the defect distribution in the LRS (a) and HRS (b). Migration of oxygen vacancies from the top electrode side to the bottom electrode side under a negative applied bias in (b) causes the top electrode reoxidation of the OEL at the top electrode (oxide B in the figure), thus causing an enhanced Schottky barrier, and hence reduced carrier injection characterizing the HRS.^67^ Reproduced with permission from ref 67. Copyright 2008 IEEE.

The most typical materials showing uniform switching are perovskites, such as manganites,^68–70^ where switching was shown to occur by oxygen transfer from the manganite layer to an active electrode, such as Al^69^ or Sm.^70^ Uniform switching can be generally recognized by the absence of an abrupt set transition and from the linearity of LRS and HRS resistance on the device area.^71^ Given the area-scaling property of the programming current, uniform switching RRAM has been considered for ultralow-power RRAM suitable in high-density 3D CBAs.^72^

### 2.5 RRAM Area Scaling

Device scaling is among the most important properties of any memory concept, to support area scaling, bit-cost reduction, and competitiveness compared to conventional CMOS-based memory concepts, such as SRAM, DRAM, and Flash. RRAM scaling has been supported by several reports, evidencing the ability to reduce both the area and thickness scaling. Figure 9a shows a top-view SEM image of a RRAM device with a TiN/Hf/HfO_*x*_/TiN RRAM stack where the size of both the top and bottom electrodes was defined in the range of 10 nm.^40^ This is shown in Figure 9b and c, reporting the TEM cross-sectional images of the device along the top and bottom electrodes, respectively. A key concern of area downscaling was shown to be the forming voltage, which tends to increase according to the Poisson area scaling of time-dependent dielectric breakdown (TDDB).^73^ To compensate for such an area dependence, the switching layer thickness can be reduced and optimized by composition profiling. For optimized scaling behavior of the forming voltage, an amorphous structure of the oxide layer is preferred compared to a polycrystalline structure, where grain boundaries might induce local nonuniformities.^40^

**Figure 9:**
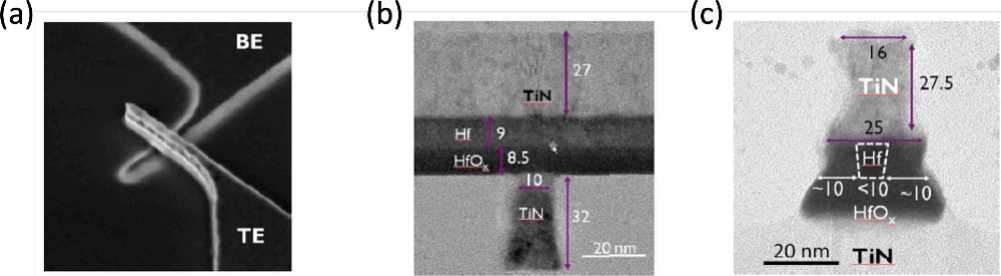
Scaling of RRAM devices. (a) Top view SEM image of TiN/Hf/HfO_*x*_/TiN RRAM device with CBA structure. (b) Cross-sectional TEM image of the same device along the top electrode direction, exhibiting the 10 nm width of the bottom electrode. (c) Cross-sectional TEM image along the bottom electrode direction, exhibiting the 10 nm width of the Hf cap at the top electrode side.^40^ Reproduced with permission from ref 40. Copyright 2011 IEEE.

Sub-10 nm scaling of RRAM devices was shown by advanced techniques based on vertical film deposition and fin exposure.^74^ RRAM CBA circuits with a 2 nm width of the top and bottom electrodes were demonstrated, while a line pitch of about 12 nm was achieved, corresponding to a device density in the range of 4.5 terabits per square inch. The switching of the TiO_2_/HfO_2_ stack was shown to occur with a low current in the range of about 50 nA.^74^

### 2.6 RRAM Based on 2D Materials

In addition to area and pitch scaling, thickness scaling is essential to enable a good aspect ratio of the device geometry and a low forming voltage. Toward this goal, RRAM with atomic thickness was demonstrated by adopting a 2D transitional metal dichalcogenide (TMD) monolayer as the switching layer.^75^ Various types of single-layer TMDs were demonstrated as switching layers, including MoS_2_, MoSe_2_, WS_2_, and WSe_2_ with Ag and Au electrodes. This device was dubbed ‘atomristor’ to highlight its ability for thickness miniaturization to the atomic scale. The TMD monolayer was deposited by chemical vapor deposition (CVD) or metal–organic CVD (MOCVD) and then transferred on the bottom electrode and completed with top electrode deposition and patterning.^75^ Figure 10a shows the sketch of the device while Figure 10b shows the cross-section of the RRAM devices, evidencing the atomically thin MoS_2_ monolayer between Au top and bottom electrodes. Figure 10c shows the crystalline atomic structure of the MoS_2_ layer, indicating the presence of S vacancies, which potentially influence the forming and switching behavior of the device.

**Figure 10:**
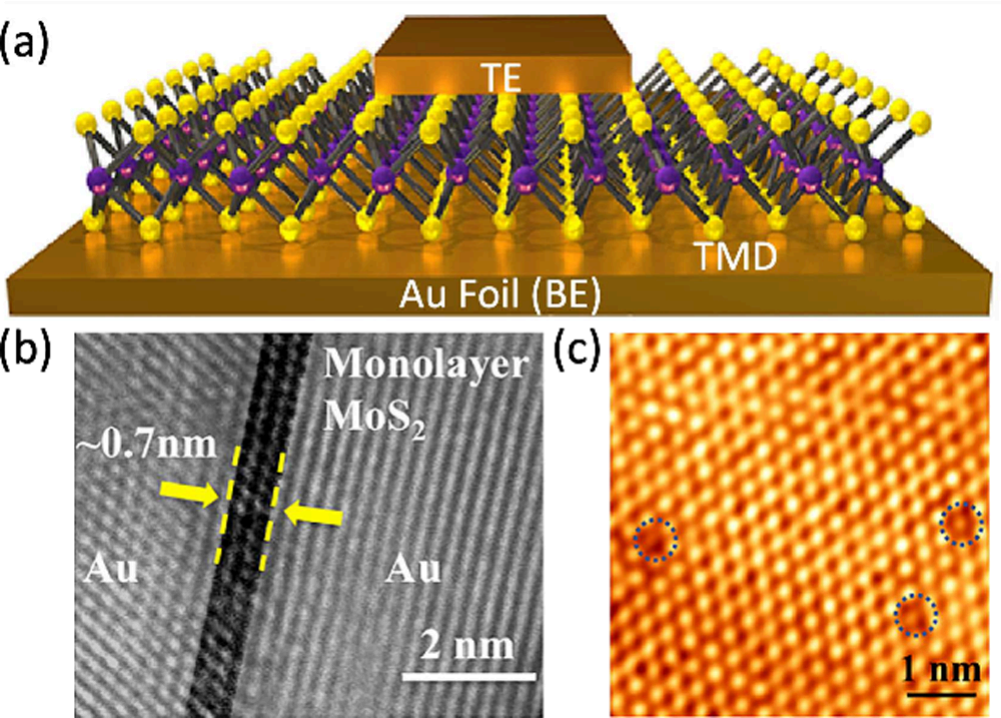
RRAM device with TMD switching layer. (a) Sketch of the device with CBA structure evidencing the TMD layer sandwiched between the top and the bottom electrodes. (b) TEM cross-sectional image illustrating the Au/MoS_2_/Au stack with monolayer thickness and atomic smoothness of the interface. (c) Scanning tunneling microscopy (STM) image of a monolayer MoS_2_ evidencing the S vacancy defects.^75^ Reprinted from ref 75. Copyright 2017 ACS.

Thickness scaling was further demonstrated in van der Waals (vdW) structures, where both the switching layer and the electrode consist of a 2D material. The vdW heterostructure graphene/MoS_2–*x*_O_*x*_/graphene was demonstrated in a RRAM device with switching endurance of up to 10^7^ and with the possibility for deposition on flexible organic substrates.^76^ RRAM devices based on hexagonal boron nitride (hBN), an insulating 2D material, were demonstrated in combination with MoS_2_-based select transistors, thus supporting the feasibility of 2D-based one-transistor/one-resistor (1T1R) memory in the BEOL at relatively low temperature.^77^ Wafer-scale integration^78^ and full-CMOS integration at the 180 nm node^79^ were recently demonstrated for hBN-based RRAM devices.

2D semiconductors provide an attractive solution as active channel materials for scalable CMOS transistors, thanks to their atomic-scale thickness and their capability for 3D, BEOL integration.^80,81^ Significant progress has been recently reported to support 2D semiconductors as a feasible technology to extend the Moore’s law of CMOS transistor scaling.^82–84^

Memory devices based on 2D semiconductors include not only RRAM but also charge-based concepts such as floating gate memories^85,86^ and charge trap memories.^87,88^ RRAM and transistor functionalities were merged in a new device named ‘memtransistor’, consisting of a 3-terminal device with a 2D-semiconductor channel controlled by a gate and contacted by source and drain.^89–92^ The device can operate as a conventional transistor, where the gate voltage enables control of the channel conductivity. However, the application of a relatively large voltage across the drain and source can result in RS of the channel conductance, similar to RRAM operation. The switching mechanism in MoS_2_-based memtransistors has been explained by the field-induced dislocation migration in the polycrystalline MoS_2_ channel^89,90^ or the modulation of the Schottky barrier at the metal–semiconductor contact.^91^

Figure 11a shows a top-view SEM image of a memtransistor device based on a MoS_2_ channel with Ag source and drain separated by a 18 nm gap.^93^ The channel conduction was controlled by the gate voltage V_*G*_ applied to the Si back gate, with a SiO_2_ layer of thickness 285 nm. To initiate the RS behavior, a forming operation was initially carried out by applying a voltage of 1.8 V across the source and drain. Figure 11b shows the *I*–*V* curves after formation, indicating a set transition at about V_*DS*_ = 0.9 V from HRS to LRS, followed by a spontaneous decay from LRS to HRS as V_*DS*_ is reduced below a characteristic holding voltage V_*hold*_ of about 0.2 V. The volatile switching can be attributed to the formation of a conductive bridge shunting the source and drain as a result of voltage-induced Ag migration on the surface of the MoS_2_ channel. The decay of the Ag CF can be explained by its instability as a result of the large surface energy, which is minimized by collapsing the elongated CF shape into isolated nanoparticles, as already shown by in situ experimental results^94^ and simulations.^95^ More detailed time-resolved studies indicate a retention time in the range of about 100 ms.^93^ Figure 11c shows the measured V_*set*_ and V_*hold*_ as a function of V_*GS*_ from Figure 11b, indicating that the set and holding voltage do not depend on the applied gate voltage, which only controls the channel leakage current in the HRS. Similar memtransistor devices were reported, although with an asymmetric structure of source and drain electrode materials.^96,97^ Thanks to the controllability of the gate and the drain, memtransistors are a promising device technology for neuromorphic computing applications.

**Figure 11:**
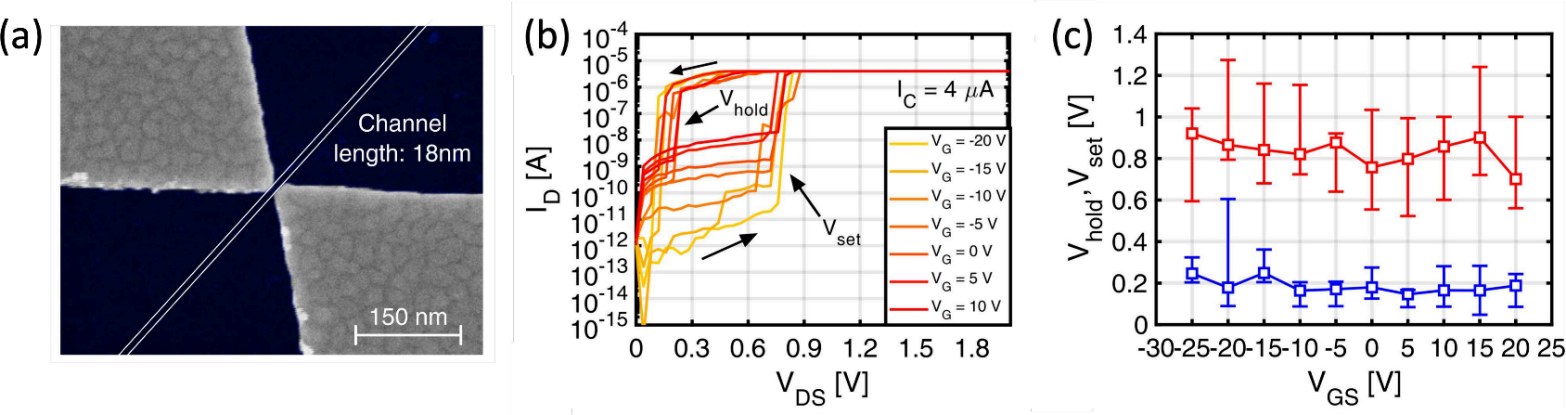
MoS_2_-based memtransistor device. (a) Top-view SEM image of the back-gated MoS_2_-based transistor with a Ag source and drain. (b) *I*–*V* curves of the set transition at V_*set*_ followed by a spontaneous collapse to the HRS at the characteristic holding voltage V_*hold*_. Changing the gate voltage affects only the HRS current without any impact on V_*set*_ or LRS resistance, with the latter being controlled by the CC. (c) Measured V_*set*_ and V_*hold*_ as a function of V_*GS*_.^93^ Reproduced with permission from ref 93. Copyright 2022 Wiley VCH.

## 3 RRAM Cell and Array Structure

For memory and computing applications, the RRAM device element can be replicated several times to realize a device array arranged in rows and columns, usually referred to as word lines (WLs) and bit lines (BLs). Figure 12 shows a summary of the various structures for the RRAM cell and the array, including one-resistor (1R) structure (a), one-selector/one-resistor (1S1R) structure (b), one-transistor/one-resistor (1T1R) structure (c), and one-capacitor (1C) structure (d).^10^ In the 1R structure, every RRAM device is connected between a row and a column of the array. While being particularly attractive from a density point of view, the 1R array, also referred to as passive CBA, is prone to disturb effects during set/reset programming and to sneakpath problems during readout.^98^

**Figure 12:**
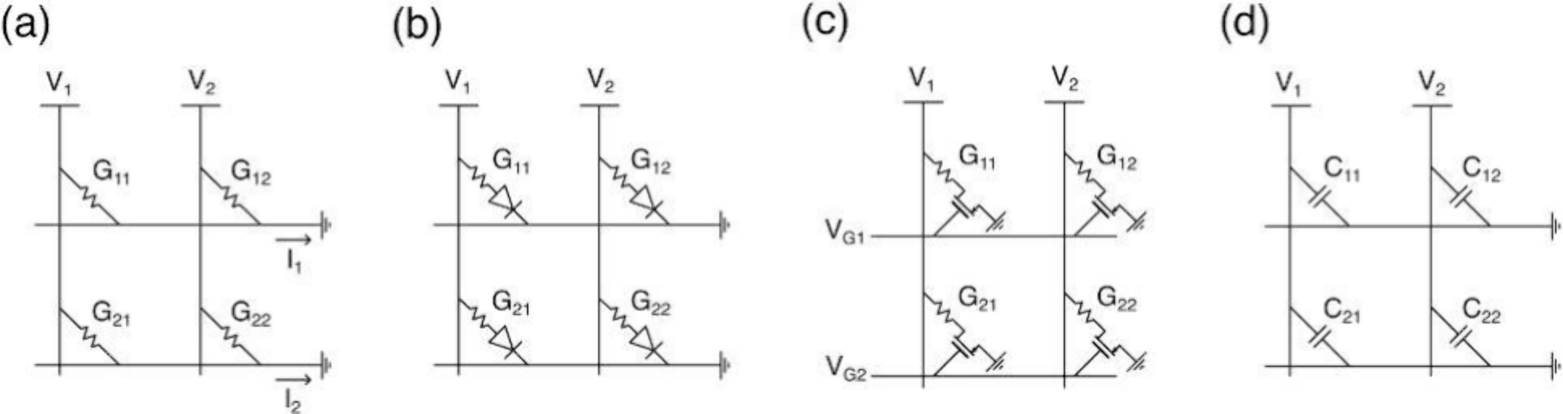
RRAM cell and array structure. (a) One-resistor (1R) array, where RRAM is connected between each row and column in the CBA. (b) One-selector/one-resistor (1S1R) array, where each RRAM element is combined with a selector device in series. (c) One-transistor/one-resistor (1T1R) array, where each RRAM element is combined with a transistor device requiring an additional line for connecting the transistor gates. (c) One-capacitor (1C) array, where the RRAM is operated as a capacitor and is connected between each row and column in the CBA.^10^ Reproduced from ref 10. Copyright 2023 AIP Publishing with Creative Commons Attribution 4.0 license https://creativecommons.org/licenses/.

### 3.1 1S1R Arrays

To prevent the sneakpath current, a nonlinear selector element can be added in series to the device in the 1S1R structure.^99–101^ Thanks to the nonlinear element, when a device is selected by applying a voltage to its row and column, all other devices are subject to a smaller voltage, which translates into an exponentially lower current.^102^

The adoption of a two-terminal selector element, such as an antifuse element,^99^ a p-n diode,^25^ or an ovonic threshold switch (OTS) device,^101^ allows maintenance of a small cell area of only 4F,^2^ where F is the lithography feature of the technology.^103^

The selector element must satisfy a number of challenging properties, including (i) a sufficient nonlinearity, to enable safe select/unselect bias schemes where the cumulated unselected device current is negligible compared to the selected device one, (ii) a sufficient on-state current, to support the programming current of the memory RRAM device in both set and reset processes, (iii) a sufficient endurance, to enable several set, reset, and read operations of the device, (iv) a low variation of threshold switching voltage and on/off-state currents, (v) a high speed to enable fast transition from select to unselect bias modes, and (vi) a bipolar operation, where the selector device can operate under both positive and negative voltage polarities to support set and reset of bipolar RRAM devices. Oxide-based p-n diodes generally display unipolar operation due to their p-n structure; thus, they are compatible only with the class of unipolar RRAM devices.^25^ Mixed ionic/electronic conduction (MIEC) devices have been shown to display a high nonlinearity combined with a bipolar operation, although their operating voltage is relatively low compared with that of typical RRAM devices.^104^ Similarly, tunneling-based selector devices with a barrier engineered stack of oxide layers show excellent bipolar characteristics with high nonlinearity, although dielectric breakdown may critically affect endurance at the high operating voltages needed to set/reset RRAM devices.^105^

Selector devices with threshold switching characteristics have also been explored with a range of different materials, including reversible insulator–metal transition metal oxides such as VO_2_ ^106^ and NbO_2_.^107^ These metal oxides display a reversible threshold switching from an off-state to an on-state, which can be used to select and unselect devices within a crosspoint array. However, the on/off current ratio is generally not sufficient to enable cell selection within a relatively large memory array. An on/off ratio of several orders of magnitude is offered by field-assisted superlinear threshold (FAST) devices^108^ and diffusive memristors consisting of a volatile RRAM device made of an Ag electrode and an oxide layer, such as SiO_2_.^109^ In this case, key concerns are the stochastic variation of the threshold voltage and the relatively long retention time for the transition from on-state to the off-state, which is generally limited by the rediffusion of cations to dissolve the conductive filament responsible for the on-state conduction.^110^ The OTS selector device shows excellent properties, including high on/off current ratio, high speed and high endurance, which must be sufficient for both the programming and the read operations.^111^ A relatively high on/off ratio is generally achieved by operating the device at a high threshold switching voltage, which, however, affects the power consumption and the design of the front-end transistors in the peripheral circuits. To improve the trade-off between threshold voltage and nonlinearity, low-voltage OTS devices were recently developed.^112^ Most recently, OTS device technology gained a renewed interest in selector-only memory (SOM) devices, where a nonlinear OTS layer can both serve as a selector and store memory states consisting of different threshold voltages.^113^ Up to 8 levels of different SOM threshold voltages were demonstrated,^114^ although the mechanism for the threshold voltage variation is still under debate.^115–117^

### 3.2 1T1R Arrays

The drawbacks of the 1S1R devices are alleviated in the 1T1R structure, where the 2-terminal selector device is replaced by a 3-terminal MOS transistor as shown in Figure 12c.^118–120^ The select transistor allows for better current control during the set transition as well as minimizing the leakage current from half-selected and unselected devices, at the expense of an additional line, usually called the WL, to access the gate terminal. Another limitation of the 1T1R structure is the need for a relatively large selector device to sustain the programming current of the device. As a result, the cell area is generally much larger than 4F,^2^ which prevents achievement of a large integration density. Figure 12d shows the 1C passive array, where the device memory bit is encoded in the capacitance instead of the device resistance, which is typical of ferroelectric materials and devices.^121^

### 3.3 RRAM Array Demonstrators

Table 1 reports a summary of RRAM technology demonstrators, namely prototypes of memory arrays with a density of at least 1 kb.^44,122–137^ Prototypes are listed for increasing years of the report, between 2011 and 2023, evidencing a consistent decrease of the technology node from 180 nm^122^ to 12 nm.^137^ All demonstrators adopted a 1T1R structure, except for ref 126, where a 1S1R structure with a high capacity of 32 Gbit was reported.

**Table 1:** Summary of RRAM Integrated Demonstrators^44,122–138^

Year	Node [nm]	Capacity	Institution	Stack	Ref
2011	180	4 Mb	ITRI	TiN/Ti/HfO_2_/TiN	^122^
2011	130	384 kb	Adesto	Ag/GeS_2_	^44^
2011	180	4 Mb	Sony	CuTe/GdO_*x*_	^123^
2012	180	8 Mb	Panasonic	TaN/TaO_2_/Ta_2_O_5_/Ir	^124^
2013	180	500 kb	Panasonic	TaN/TaO_2_/Ta_2_O_5_/Ir	^125^
2013	24	32 Gb	Sandisk/Toshiba	Metal Oxide	^126^
2014	28	1 Mb	TSMC	Metal Oxide	^127^
2014	27	16 Gb	Micron/Sony	Cu-based/oxide	^128^
2015	90	2 Mb	Renesas	Metal/Ta_2_O_5_/Ru	^129^
2017	90	500 kb	Winbond	TiN/HfO_2_/Ti/TiN	^130^
2018	40	11.3 Mb	TSMC		^131^
2019	22	3.6 Mb	Intel		^132^
2020	22	13.5 Mb	TSMC		^133^
2020	28	500 kb	TSMC		^134^
2020	28	1.5 Mb	TSMC/IMECAS		^138^
2021	14	1 Mb	IMECAS	Cu-based/oxide	^135^
2022	28	800 kb	Infineon/TSMC		^136^
2023	12	1 Mb	TSMC		^137^

Figure 13 shows the array capacity (a) and the technology node (b) as a function of the year of the demonstrator. In most cases, the prototypes in Table 1 display relatively small capacity, aiming at the demonstration of eNVM capable of being integrated into the same chip as analog and digital circuits for sensing and processing, such as microcontroller units (MCUs). RRAM technology is among the most promising thanks to the BEOL integration requiring only metal and insulator layers for the active cell, while the CMOS select transistor can be integrated in the front-end of the line. This solution allows for overcoming the difficult integration of Flash devices in advanced CMOS nodes beyond the 28 nm node, where CMOS transistors adopt the high-k/metal-gate (HKMG) process.^139^

**Figure 13:**
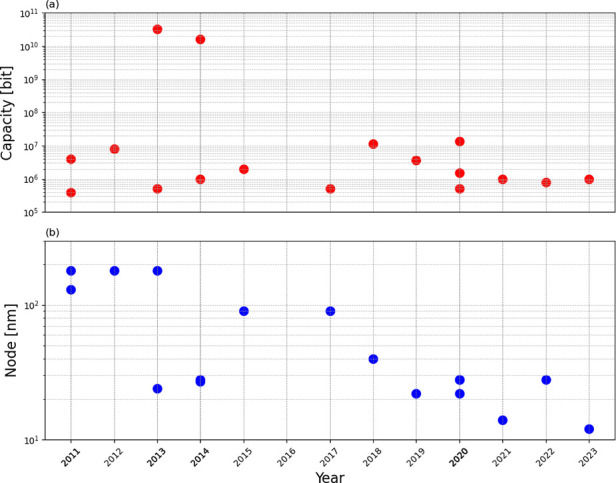
Summary of RRAM technology scaling according to Table 1. (a) Array capacity and (b) technology nodes of the reported RRAM demonstrators.

In just two cases, the capacity in Table 1 exceeds the Gbit level, which evidences the effort to achieve RRAM arrays with high capacity approaching the typical range of Flash and DRAM.^126,128^ This was possible thanks to an extremely small cell area of 4F^2^ for the 1S1R structure^126^ and 6F^2^ for the 1T1R structure.^128^

This technological trend generally goes under the name of storage class memory (SCM), which identifies a memory technology capable of filling the gap in the memory hierarchy between volatile DRAM, characterized by relatively high performance and relatively large area, and nonvolatile Flash NAND storage, characterized by relatively small area, low cost and slow access times.^140^

Figure 14a shows the TEM cross-section along the BL direction of the 1T1R array with 6F^2^ cell area in the 27 nm node.^128^ The cross-section evidences the V-shaped recess access transistors with elongated channels and buried WL and the RRAM devices sharing the same TE line, which minimizes the cell footprint along the BL direction. Figure 14b shows the cumulative distributions of the measured read current for the LRS and the HRS after 10^3^ cycles for various cells, namely the integrated cell in Figure 14a, the intrinsic cell with larger RRAM active area and the scaled cell with larger pitch.^128^ The distributions show a similar shape and similar read window, suggesting that the integration process does not significantly affect the RRAM cell behavior. Note the relatively large statistical spread of the HRS read current, which makes the effective read window relatively small in the large array.

**Figure 14:**
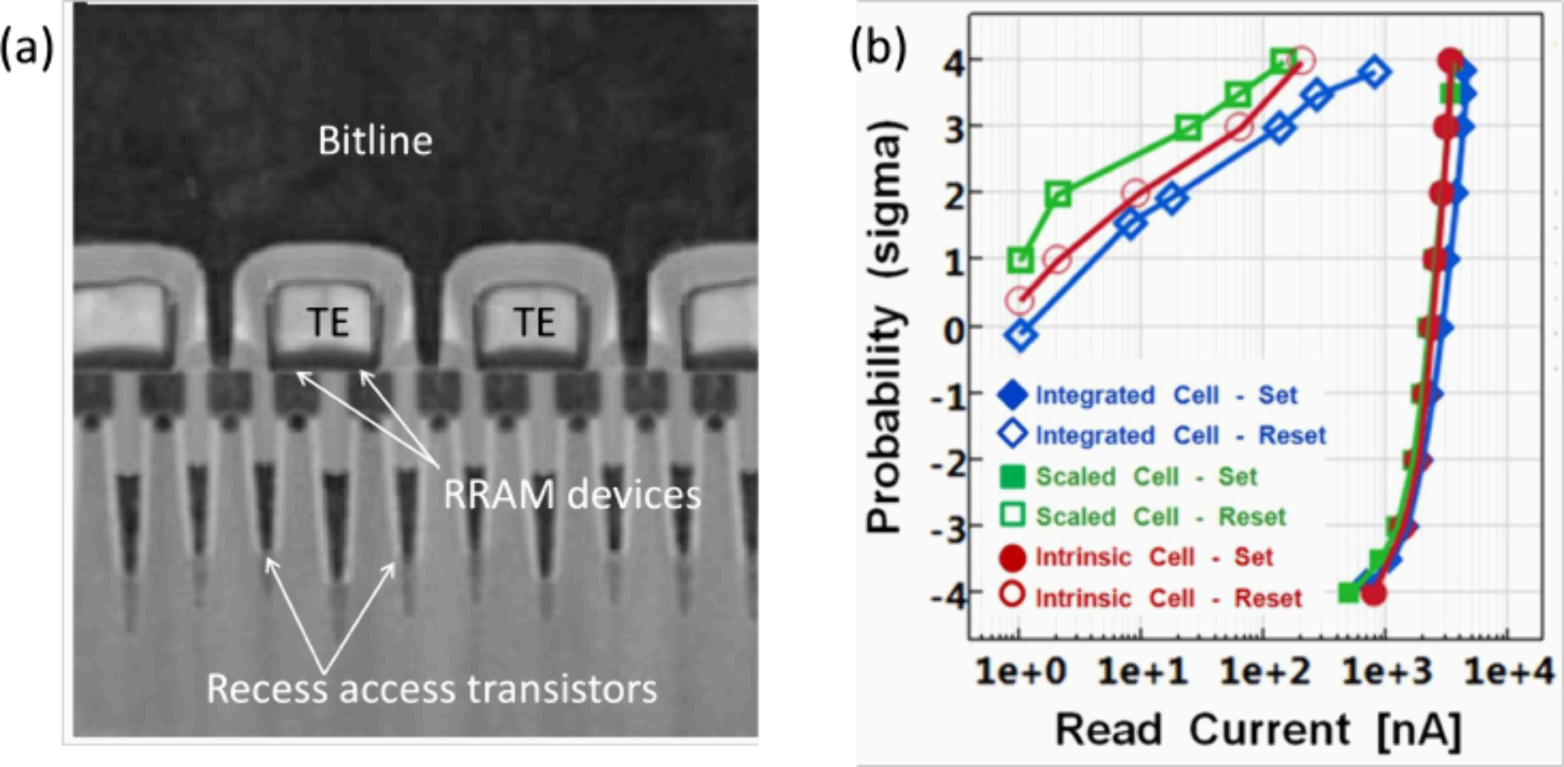
1T1R RRAM array with 6F^2^ cell area. (a) TEM cross-section of the array along the bit line (BL), evidencing the select transistors with recess geometry and the RRAM devices sharing the TE. (b) Cumulative distributions of read current for the LRS and HRS for the intrinsic cell, integrated cell and scaled cell after 10^3^ cycles.^128^ Reproduced with permission from ref 128. Copyright 2014 IEEE.

An even higher capacity was achieved by the 1S1R array in ref 126 thanks to (i) the 2-terminal structure of the selector element thus enabling a CBA architecture and (ii) the 3D stacking, where two devices occupy the same cell area. This is the horizontal 3D approach evidenced in Figure 15a,^141^ where multiple CBAs are stacked on top of each other to minimize the effective cell area and, hence, maximize the bit density. A horizontal 3D RRAM array with 6 layers was demonstrated with a Cu/Ta/TaN/TaON/Cu stack in 28 nm HKMG CMOS technology.^142^ A similar horizontal 3D approach has been pursued in the 3DXP technology consisting of stacked 1S1R CBAs of a PCM element combined with an OTS selector.^143,144^ However, horizontal stacking is prone to layer-to-layer variation due to thermal degradation during the fabrication process. Most importantly, the process yield decreases sharply with the number of stacked layers, due to the repetition of critical lithography masks, similar to the case of 3D flash NAND technology.^145^

**Figure 15:**
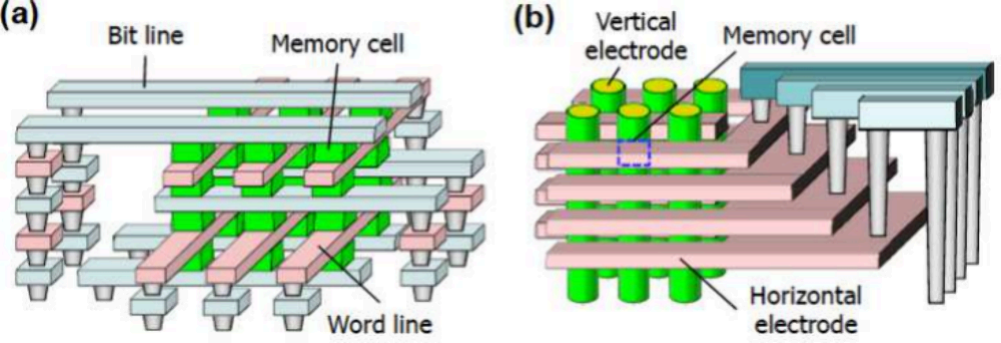
3D RRAM arrays. (a) Horizontal 3D RRAM array, where two or more CBAs are stacked to share the same area. (b) Vertical RRAM array, where the memory cells are obtained at the interface between vertical and horizontal electrodes.^141^ Reproduced with permission from ref 141. Copyright 2011 IEEE.

To maximize the bit density while maintaining a high process yield, the vertical 3D RRAM technology of Figure 15b was developed.^141^ Here, each bit cell is located at the crossing between a horizontal metal plane or electrode and a vertical electrode. The processing yield is maximized with this approach, since there is just one critical lithography mask to realize the vertical holes to be filled with the oxide/electrode stack. 3D vertical RRAM concepts were demonstrated with both filamentary-type HfO_*x*_-based RRAM^146^ and uniform-switching RRAM with TiO_2_/TaO_*x*_ bilayers (see also Figure 7c,d,e^55,58,59^ ). In all vertical 3D RRAM implementations, conformal deposition techniques such as ALD become critical for the deposition of the vertical oxide/electrode stack within holes or trenches with a high aspect ratio.

## 4 RRAM Circuits for Computing

RRAM has been initially developed for memory applications and identified as a promising technology for SCM^140^ and eNVM for consumer, industrial^147^ and automotive microcontrollers.^148^ Besides the pure memory application, RRAM can provide an enabling technology in computing applications, where the memory plays a crucial role within the von Neumann architecture. In fact, bringing the compute function near (or even inside) the memory can provide several advantages for data-intensive computing tasks.^1,3,4,10^ Such a memory- or data-centric approach, as opposed to the conventional compute-centric one, is a promising paradigm to accelerate modern computing tasks such as data search, data analytics, machine learning, and artificial intelligence (AI).^149^ This constitutes the so-called *in-memory computing* (IMC) concept, where computing in situ within the memory can alleviate or suppress the data movement which is responsible for most of the energy consumption and latency in conventional digital computing systems.^2^

RRAM features several advantages for IMC, such as high density, scalability, low-power operation, nonvolatile storage, multilevel operation, and CMOS-compatible BEOL integration. Memory applications in computing generally rely on the ability to perform analog-domain operations with high parallelism within the RRAM array, typically exploiting Kirchhoff’s law for summation and Ohm’s law for multiplication.^4^ Several concepts for such physical computing within RRAM have been proposed in the literature, as summarized in Figure 16.^10^ The schematic *I*–*V* curve in Figure 16a highlights two potential regimes for IMC operation, namely (i) the static regime at low voltage, where RRAM can store a pretrained, preprogrammed parameter for computation (Figure 16b), and (ii) the dynamic regime at high voltage across the switching regime where the device can dynamically change its programmed state to mimic spike integration, learning, adaptation, and other linear or nonlinear functions (Figure 16c).^10^

**Figure 16:**
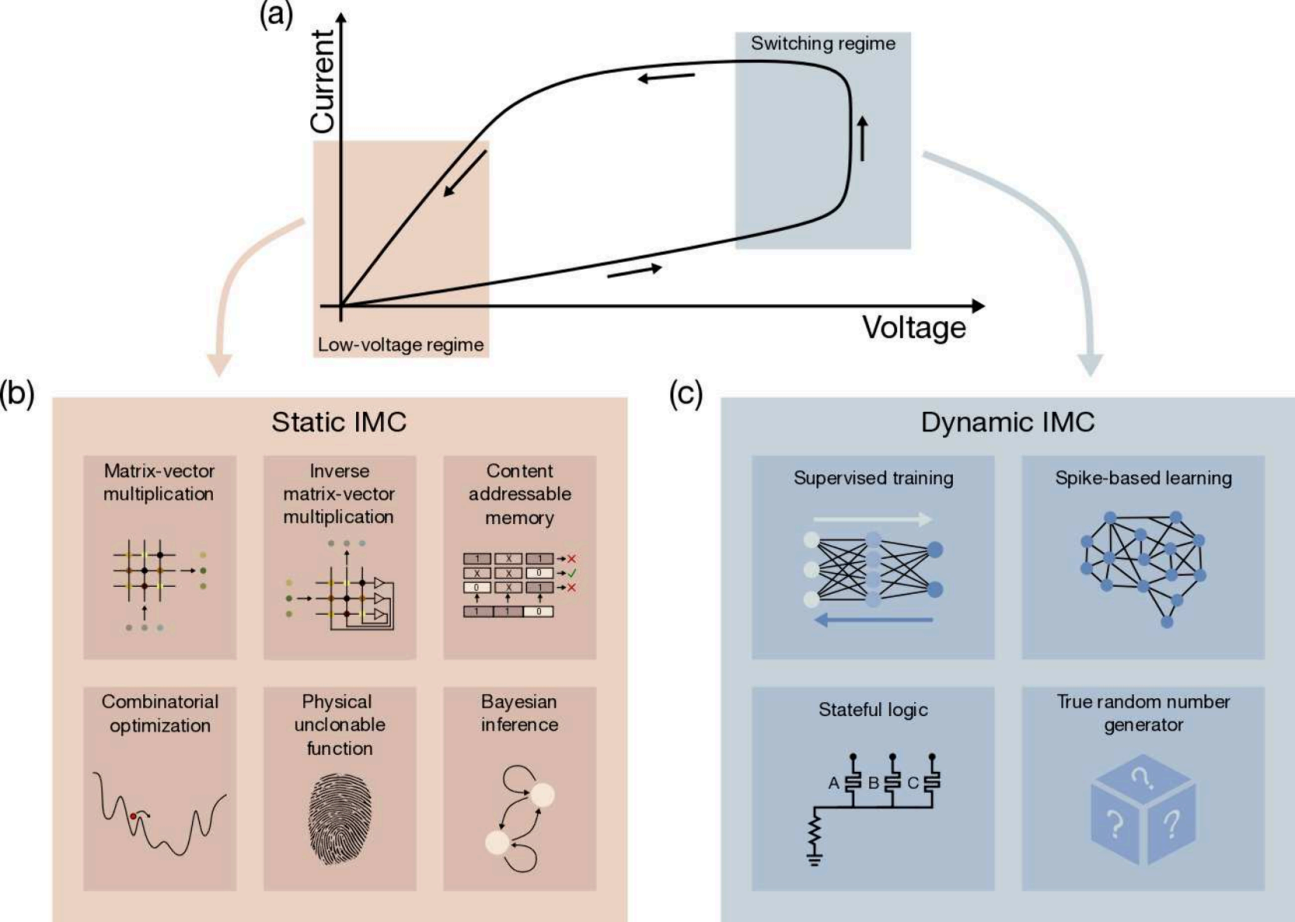
Applications of RRAM Devices for in-memory computing (IMC). (a) Schematic *I*–*V* curve of a RRAM device with low-voltage and high-voltage regimes, corresponding to static and dynamic IMC, respectively. (b) Examples of static IMC, where pretrained parameters are stored in the memory to perform physical computation tasks, such as matrix-vector multiplication (MVM). (c) Examples of dynamic IMC, where pulses are applied in the switching regime to induce dynamic changes in the conductance for reproducing adaptation, learning, and other types of linear/nonlinear computing functions.^10^ Reproduced from ref 10. Copyright 2023 AIP Publishing with Creative Commons Attribution 4.0 license https://creativecommons.org/licenses/.

Among the static IMC functions, matrix-vector multiplication (MVM) is probably the most popular and explored, due to its implication in the deep neural network (DNN) for both the inference and the training processes.^4^ Similarly, a RRAM CBA can be used for inverse MVM, where a linear system is solved, thus facilitating the calculation of inverse, pseudoinverse matrices, eigenvectors and singular value decomposition.^150^ Other static functions include content addressable memory (CAM) for data search and query,^151,152^ combinatorial optimization,^153,154^ physical unclonable function (PUF)^155,156^ and Bayesian inference.^157^

Dynamic IMC aims at exploiting the programming property of the RRAM to reproduce dynamic functions, such as nonlinear neuron activation,^158^ stateful Boolean logic gates,^159,160^ synaptic plasticity,^161,162^ and learning in supervised/unsupervised neural networks.^162–165^ Typically, the dynamic regime leverages controlled switching close to the set or reset voltage to modify the conductance of the RRAM device in response to the applied pulse width and amplitude. Randomized switching in the dynamic set/reset range can be used to develop circuits for true random number generation (TRNG).^166–168^ Steep-slope logic devices have also been proposed based on the abrupt set transition in the dynamic regime of RRAM devices.^169^

A key issue with dynamic RRAM computing is the limited set/reset endurance of RRAM devices as well as the energy consumption required by the set/reset operations. On the other hand, static IMC provides nonvolatile storage of computational weights for the execution of standardized tasks, such as neural network inference. Dynamic and static IMC can be generally combined in the same platform to provide energy-efficient processing capable of learning and adaptation.^158,162^

### 4.1 RRAM Crossbar Arrays for Matrix-Vector Multiplication

The CBA circuit of RRAM devices has been widely used for accelerating MVM, or dot product, which, among various use cases, is one of the dominating bottlenecks in accelerating inference and training of neural network models. Figure 17a shows a circuit for performing MVM based on CBAs, namely a dot-product engine (DPE).^118^ The CBA can be a passive 1R array (Figure 12a) or a selected-memory CBA, such as the 1S1R array (Figure 12b) or the 1T1R array (Figure 12c). Thanks to the possibility of programming analog parameters into a RRAM device, the RRAM CBA can be adopted as a physical transcription of a matrix, where each RRAM cell serves as a matrix entry. A matrix $A\in Z^{N\times M}$ is programmed in the CBA such that each RRAM conductance is given by *G*_*ij*_ = *A*_*ij*_ × *G*_0_, where *G*_0_ is a suitable unit conductance. An analog voltage vector $v\in Z^{N}$ is applied to the rows that are connected to the RRAM top electrodes. By connecting the *M* columns at ground, the resulting vector of currents *i* is given by

**Figure 17:**
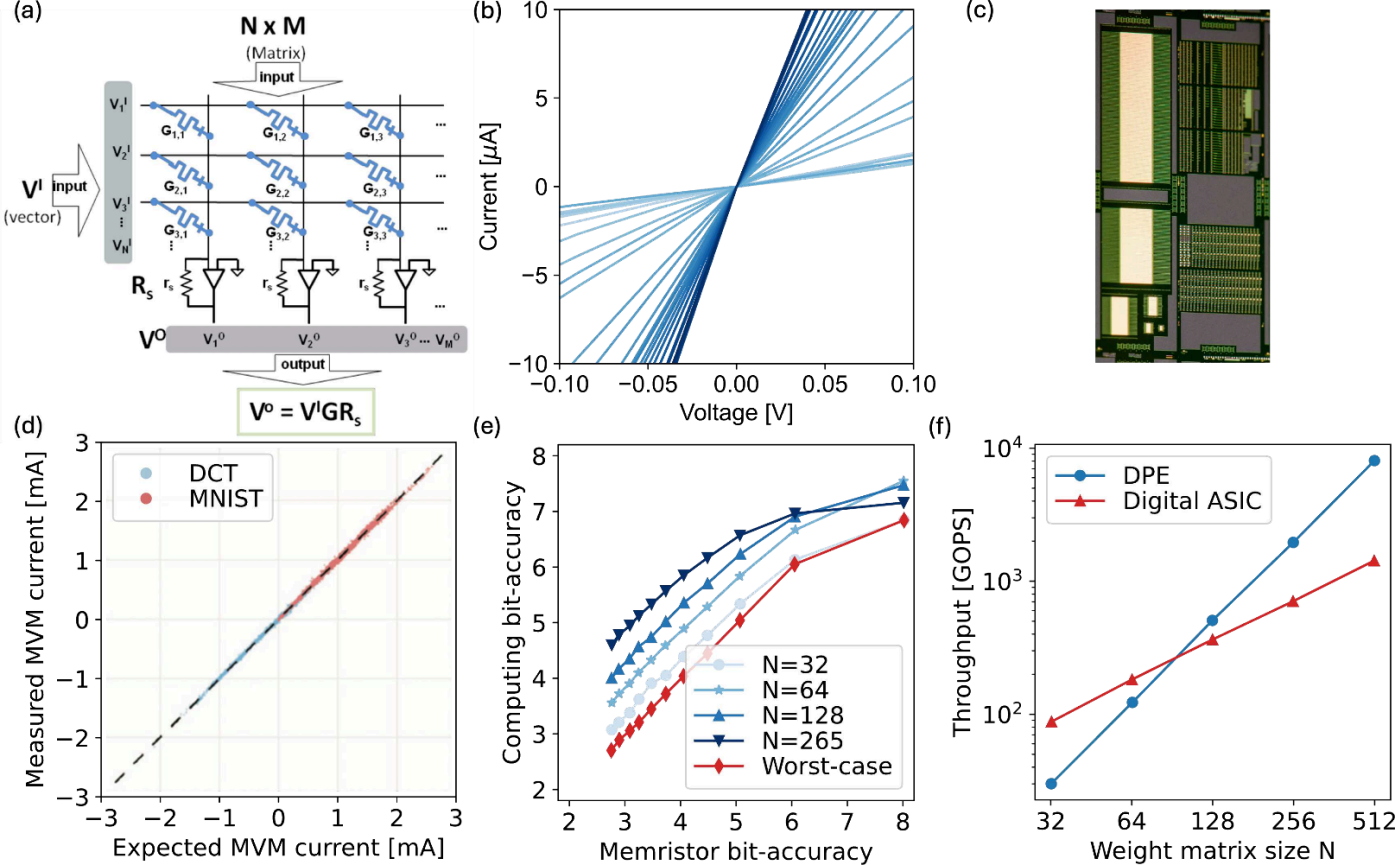
Matrix-vector multiplication (MVM) with RRAM CBAs. (a) Circuit schematic of a dot-product engine (DPE) with RRAM devices. Input voltages are applied on the TE (rows), and currents are accumulated on the columns and sensed using a transimpedance amplifier (TIA). (b) *I*–*V* curves of the RRAM devices programmed in multiple conductance states, indicating both the multiple achievable states and the linear (ohmic) conduction for relatively high conductance. (c) Image of an integrated circuit including multiple 1T1R arrays of various sizes. (d) Correlation plot between the experimentally measured MVM with linear correction and the ideal MVM performed in software. (e) Equivalent MVM bit-precision as a function of memristor bit accuracy for multiple array sizes, i.e., the number of rows in the CBAs. (f) MVM throughput as a function of array size of DPE and digital ASIC. Panels (a,c,e,f) are adapted with permission from ref 118. Copyright 2016 Association for Computing Machinery. Panel (b) is adapted from ref 170. Copyright 2021 Nature Publishing Group with Creative Commons Attribution 4.0 license http://creativecommons.org/licenses/by/4.0/. Panel (d) is adapted with permission from ref 171. Copyright 2018 Wiley VCH.

2 i=vG

which corresponds to a MVM or dot product. The output current *i* can be sensed by a transimpedance amplifier (TIA) which converts the current into a voltage, which can, in turn, be converted into a digital word with an analog-to-digital converter (ADC).

Figure 17b shows the current–voltage characteristic for several RRAM devices in the read (low-voltage) regime, with multiple stable conductance levels having ohmic, i.e. linear, conduction,^170^ which is essential for performing dot products without errors due to nonlinear parasitic effects. To avoid nonlinearity issues due to the non-ohmic behavior of RRAM devices for some states, a 1T1R structure can be used with the TE voltage fixed to a convenient voltage *V*_*read*_ and the gate voltage equal to the logic binary input, while the summation current is accumulated along the column. After conversion of the current to a digital word, further summation can be achieved with the help of a shift-and-add operation in the digital domain. Such a binary-input approach also comes with the advantage of effectively eliminating the need for a digital-to-analog converter (DAC) at the CBA input rows, with benefits of reduced area and improved energy efficiency.

Figure 17c shows the physical implementation of multiple CBAs with a maximum size of *N* = 64 rows and *M* = 128 columns of 1T1R cells.^171^ The chip in the figure was used to demonstrate an on-chip MVM and to assess the impact of the array size on the accuracy of the dot-product operation. Figure 17d shows a correlation plot of the measured analog output as a function of the expected *software* output. A linear correction was applied to address the column-wise error due to IR-drop, and the results show a good agreement between the software and experiments. However, as the array size increases, the impact of the parasitic wire resistance of the TE and BE becomes increasingly relevant, thus limiting the effectiveness of linear correction. In fact, the array wire resistance causes a current resistance (IR) drop along the rows and columns. Considering the same current *I* flowing in each device, the voltage drop Δ*V*_*IR*_ across the wire can be estimated by

3 $$\Delta V_{IR}=rI+2rI+...+NrI=\frac{rIN^{2}}{2}$$

where *r* is the cell-to-cell wire resistance.^172^ Considering, for instance, *r* = 1 Ω, *I* = 10 μA and *N* = 128, we obtain an estimate for the total *IR* drop along the line of Δ*V*_*IR*_ ≈ 8 mV, which can contribute significantly to the dot-product error. By performing a dot product on arrays of different sizes and comparing it with the error obtained by performing a digital MVM with reduced precision, such as fixed-point INT8 or INT4, it is possible to correlate the array size with a given bit precision, as shown in Figure 17e. If only one value is programmed per column, the computational accuracy equals the RRAM accuracy (worst case); however, 7-bit computational precision can be reached for dense matrices of 6-bit RRAMs, given that noise is assumed uncorrelated among multiple devices. Computational accuracy saturates to 8-bit even for a large number of bits stored in the RRAM, due to the *IR* drop becoming the dominant factor compared to device noise. By properly modeling the *IR* drop, it is possible to introduce compensation techniques at both circuit level^173,174^ and system level.^175^ Large arrays are desirable to maximize the equivalent throughput, defined as the number of operations (two in the case of multiply accumulate) performed in a unit of time. Figure 17f shows the throughput as a function of matrix size *N* for a RRAM-based DPE^171^ and a digital ASIC,^176^ demonstrating that DPE can reach a higher computing speed than a digital counterpart for *N* ≈ 128, and be 10 times faster for *N* > 512.

### 4.2 RRAM CBA Circuits with Analog Feedback for Inverse MVM

By modifying the analog peripherals of the CBA, it is possible to perform inverse linear algebra operations, such as computing the solution of linear systems,^150^ extracting the eigenvectors of a matrix,^177^ performing linear regression^178,179^ and others. Figure 18a shows the circuit schematic of the CBA and its analog peripherals required for computing the solution of a linear system. The columns and rows of the CBA are connected to the input and output terminals of operational amplifiers, respectively, to provide a feedback loop. The conductance *G*_*ij*_ values of the RRAM devices in the CBA are programmed with the coefficients of a positive-definite matrix *A*; then a current vector *i* is injected at the column terminals, which are kept at virtual ground potential by the feedback loop. The application of the input current pulse stimulates an analog output voltage vector *v*:

**Figure 18:**
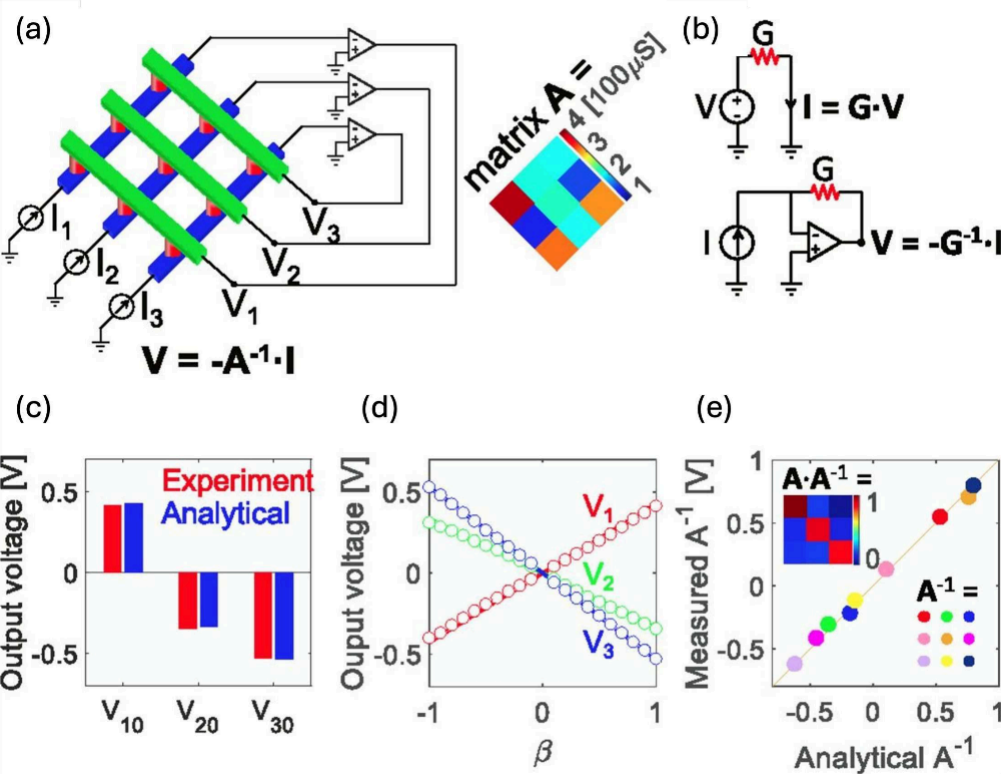
Inverse MVM in CBAs with a feedback loop connection. (a) Circuit schematic of a closed-loop CBA for the solution of linear systems. The inset shows a programmed 3 × 3 matrix. (b) The conceptual schematic highlighting the difference with conventional circuits for the direct operation of CBAs. (c) Experimental and analytical output voltages for the solution of a linear system. (d) Output voltage as a function of the input parameters β with *i*_*in*_ = *βi*_*ref*_. (e) Correlation plot of analytical and measured inverse matrix computation. Reproduced with permission from ref 150. Copyright 2019 National Academy of Sciences.

4 $$v=-G^{-1}i$$

which is the same as eq 2 but referred to the output voltage. Equation 4 provides the solution of the linear system with matrix *A* = *G* and the known vector *i*.^150^

Figure 18b shows the concept of this circuit and its relationship with the open-loop MVM of Section 4.1. While the MVM circuit is similar to the simple case of a voltage applied to a conductance, resulting in a scalar product *I* = *GV*, the inverse MVM case resembles the TIA circuit, where the applied current is converted to a voltage *V* = −*G* ^–1^ *I* thanks to the concept of feedback loop enabled by the operational amplifiers.^150^

Figure 18c shows an experimental demonstration of the concept, with the measured output of a linear system with 3 equations closely matching the analytical result. The result is confirmed for various inputs in Figure 18d, where parameter β provides the relative amplitude of the applied input current. The same circuit can be used for computing the inverse of a matrix by applying the vectors of an identity matrix as input and collecting the various obtained output voltage vectors to form the inverse matrix.^150^ Figure 18e shows the correlation plot comparing the elements of the inverse matrix computed with the analytical formula to those obtained from the experimental output voltage, indicating a good accuracy of the inverse matrix circuit. This circuit with the CBA in analog feedback can be used for various applications beyond the solutions of linear systems, as further illustrated in Section 5.4.

### 4.3 Content Addressable Memories

The content addressable memory (CAM)^180^ is a fundamental memory structure that operates in a complementary way with respect to the random access memory (RAM). As shown in Figure 19a, reading a RAM circuit consists of selecting an address as input and obtaining a data bit stored at the address location as output. On the other hand, reading a CAM requires that a content is presented as input, while the output yields the memory address where that specific content is stored. Figure 19b shows a ternary CAM (TCAM), where each cell verifies whether the input is equal to the stored value. A wildcard (‘X’, or *don’t care*) is added to match both 0s and 1s as input. If all of the TCAM cells in a row are matched, a match value is returned on the match line (ML), which can be then converted into the specific address.

**Figure 19:**
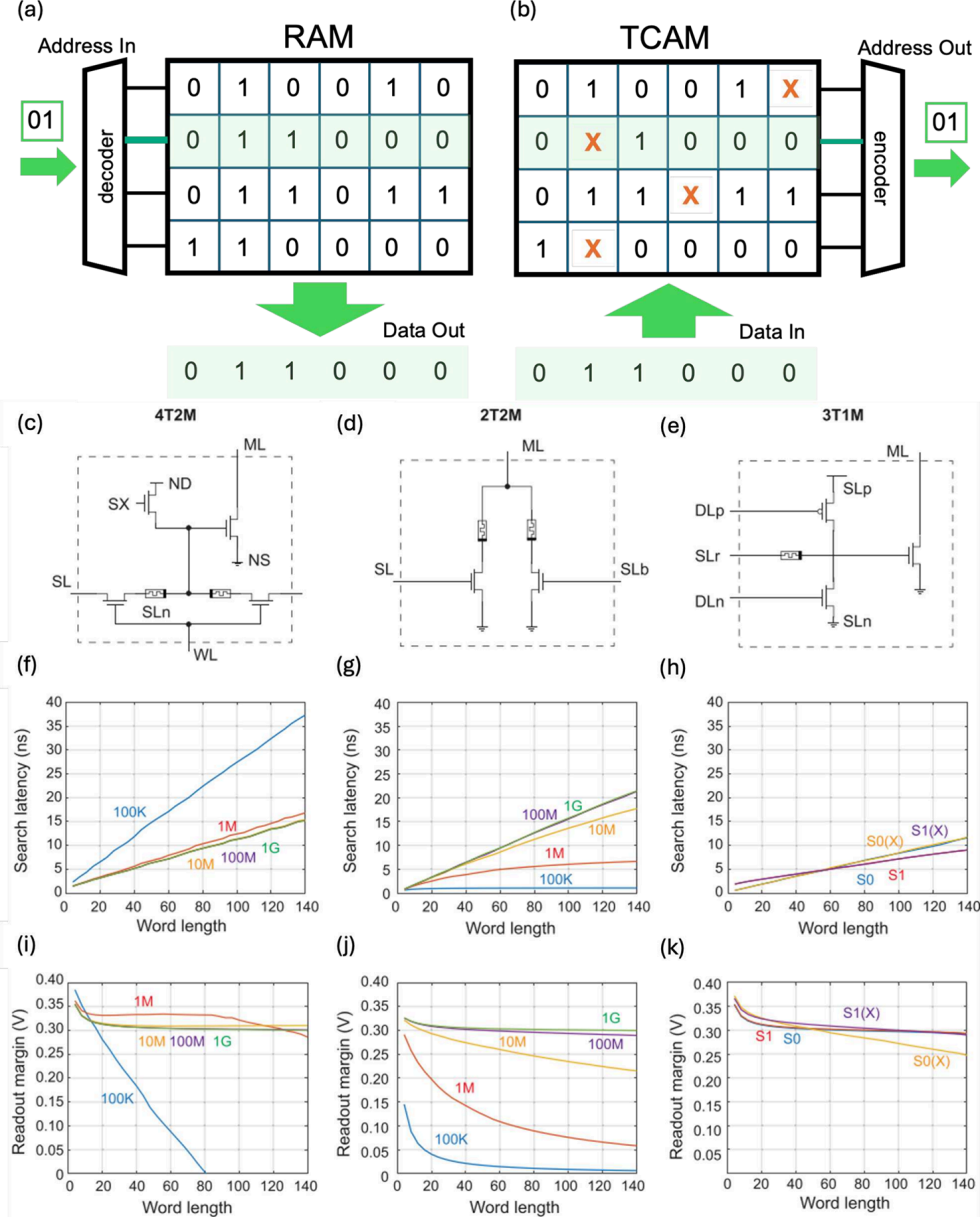
CAM architectures. (a) Conceptual circuit schematic of the RAM. (b) Conceptual circuit schematic of TCAM. (c) TCAM cell with 4T2M structure. (d) TCAM cell with 2T2M structure. (e) TCAM cell with 3T1M. (f,g,h) Search latency for 4T1M, 2T2M, and 3T1M cell structures, respectively. (i,j,k) Search margin for 4T1M, 2T2M and 3T1M cell structures, respectively. The RRAM resistive window Δ*R* was changed to assess its impact on the circuit performance. Adapted with permission from ref 152. Copyright 2019 IEEE.

TCAMs are ubiquitous in several applications, particularly in networking.^181,182^ However, the broader use of TCAMs for computing has been hindered by the large area and power consumption of SRAM-based TCAMs, which require 16 transistors for storing and searching ternary values. From this viewpoint, RRAM-based TCAM implementation is attractive, as it enables a smaller cell area and hence a higher bit density. Various designs of RRAM-based TCAM cells have been proposed, including 4-transistors/2-memories (4T2M, Figure 19c),^152^ 2-transistors/2-memories (2T2M, Figure 19d),^183^ and 3-transistors/1-memory (3T1M, Figure 19e).^151^ In general, a TCAM circuit is operated by connecting all the input search line (SL) terminals along the column direction and all the output ML terminals along the row direction for creating the array in Figure 19b. For the TCAM search operation, the ML is initially precharged to a convenient potential, and then the input data are applied to the SL. If the input data match the value stored in the RRAM devices, the ML remains at the precharged potential; otherwise, a pull-down transistor is activated to discharge the ML. A sense amplifier connected to the ML is used for sensing and latching the output after a given search time.

In the case of the 4T2M TCAM in Figure 19d,^183^ two RRAM devices are programmed to represent a 0, 1, or X in [HRS,LRS], [LRS,HRS] or [LRS,LRS], respectively. As an example of the TCAM operation, when a ‘1’ is applied to the SL (and a ‘0’ on SLn), if a ‘0’ is stored, then a voltage divider between the two RRAMs activates the pull-down transistor, thus causing the discharge of the ML. A similar behavior can be derived for other cases such as search ‘1’ store ‘1’, search ‘0’ store ‘0’, and search ‘0’ store ‘1’. In the case of an ‘X’ stored, the voltage divider node is the mean voltage which is tuned to be below the pull-down threshold. In the case of the 2T2M cell, the encoding of the cell state into the RRAM device is the same; however, the input transistors act directly as pull-down transistors, thus avoiding the need for an additional transistor.

The 3T1M cell utilizes three RRAM states, namely, LRS, medium resistance state (MRS), and HRS to store 1, X, and 0, respectively. When a ‘1’ is searched, both data lines (DL) are kept at the ground, with only SLp at 1. If the cell stores a 0, most of the voltage drop is on the RRAM, thus activating the pull-down node and vice versa in the case where a 1 is stored. The conductance of the MRS is tuned such that the voltage drop on the RRAM device is not sufficient to activate the pull-down transistor. Note that TCAM operations can also be emulated by conventional CBAs,^156^ although this requires significant additional peripheral overhead.

Figure 19f,g,h shows the worst-case search latency as a function of array word length for 4T2M, 2T2M, and 3T1M simulated with the same technology node, i.e. CMOS 180 nm.^152^ The worst case is defined as a 1-bit mismatch since only one pull-down transistor is activated to remove the charge from the ML, thus resulting in a relatively long search time. Search time scales linearly with the word length since a drain-source parasitic capacitance on the ML is added for each cell on a row, increasing the overall ML capacitance. Figure 19i,j,k shows the read margin as a function of the word length for 4T2M, 2T2M, and 3T1M, respectively, under a similar simulation on the CMOS 180 nm technology node.^152^ The readout margin is defined as the difference between the voltage on the ML during the worst-case match (all ‘X’s) and the worst-case mismatch (1-bit). Longer columns have larger leakage, thus significantly reducing the read margin, due to the nonactivated pull-down transistors providing a parasitic contribution to the discharge of the ML.

While 2T2M has the most compact structure, it requires a large HRS (e.g. >1 MΩ) to minimize the parasitic discharge leakage and, hence, maximize the array size. On the other hand, the 3T1M architecture provides the fastest response, which comes at the cost of a relatively large static power consumption during search operation.^151^ The 4T2M cell design displays a larger area; however, it can provide a suitable trade-off between conductance window requirements, latency, and power consumption. The proposed RRAM-based TCAM cells have been demonstrated in various compute applications, including regular expression matching,^184^ genomics,^185^ and hyperdimensional computing.^186,187^

By leveraging the analog operation of emerging NVMs, an analog CAM (Figure 20a) was recently proposed,^188^ where TCAM columns are merged in *ranges*, to return a match in a cell if the analog input is within the stored range. For this purpose, two RRAM devices can be used for the lower and upper bound, as shown in the circuit of Figure 20b with a 6-transistors/2-memories (6T2M) design. If the input voltage applied to the DL is high enough, T1 is turned on, effectively pulling down the gate of T2, which is switched off and thus plays no role in affecting the ML, which returns a match. Figure 20c,d shows the lower and upper bound circuits. The lower bound circuit operation is shown in Figure 20e with the voltage on the gate of the lower bound pull-down transistor as a function of the input voltage on the data line (DL) for multiple programmed conductances in M1. By increasing the M1 conductance, it is possible to move the lower bound to a higher value. Complementary to the lower bound, the upper bound is realized by adding an inverter between the voltage divider on M2 and the pull-down transistor. Figure 20f shows the voltage on the upper bound pull-down gate G2 and a function of the input voltages for multiple conductance values programmed on M2. Similarly, by increasing the conductance on M2, it is possible to extend the upper bound. Analog CAM has been demonstrated for the acceleration of multiple compute workloads, including tree-based machine learning,^189^ one-shot learning^190^ and query processing.^191^

**Figure 20:**
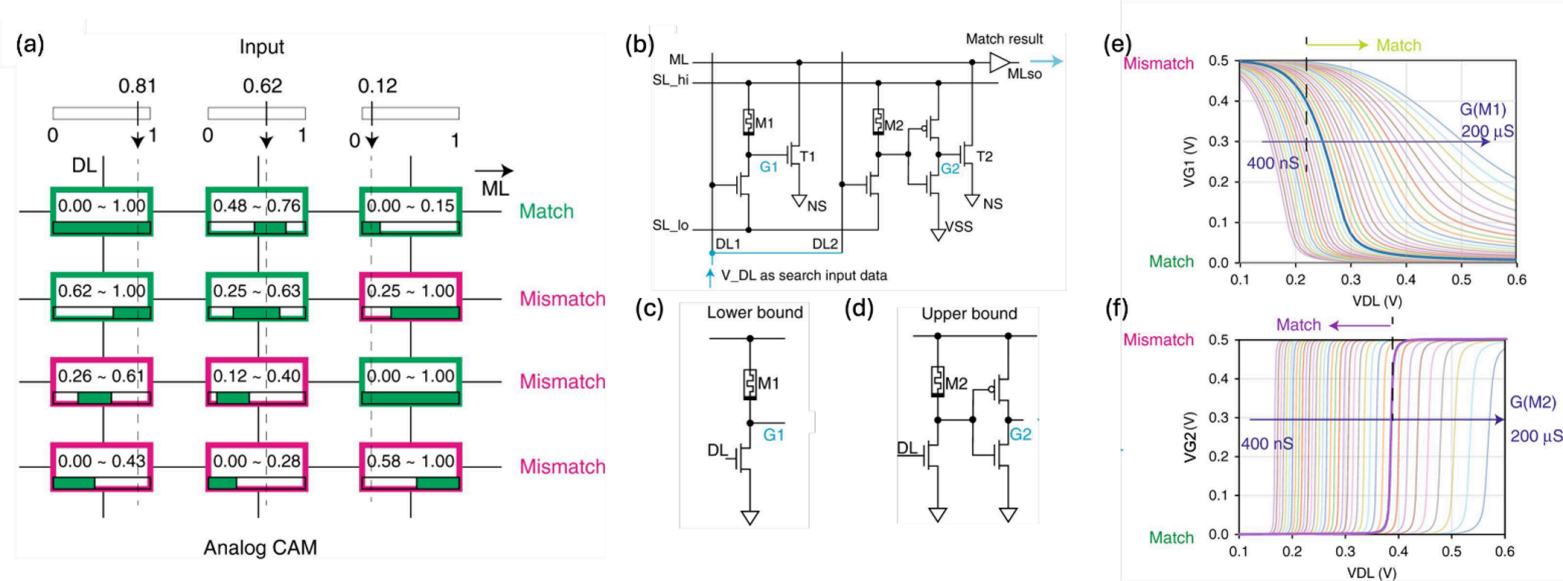
Analog CAM concept. (a) Conceptual circuit schematic. (b) Circuit design of the 6T2M TCAM cell. (c) Lower bound subcell controlling the G1 pull-down transistor. (d) Upper bound subcell controlling the pull-down transistor G2. (e) Lower and (f) upper bound pull-down voltages as a function of input voltage V_*DL*_ for multiple programmed conductance. Adapted from ref 188. Copyright 2020 Nature Publishing Group with Creative Commons Attribution 4.0 license http://creativecommons.org/licenses/by/4.0/.

## 5 Computing Applications

IMC is extremely promising for the execution of data processing tasks directly in the memory, thus reducing the energy consumption and taking advantage of the extreme parallelism and analog operation of the memory array circuit. Table 2 summarizes the most relevant computing applications that have been explored for IMC. The mainstream applications attracting widespread interest are inference and training of AI models, such as DNNs and large language models (LLMs). Other computing applications include solving linear equations, linear regression problems, principal component analysis (PCA), decision trees, combinatorial optimization of complexes, multiple-variable problems, stochastic computing and spiking neural networks in neuromorphic computing. Each of these computing applications generally relies on a different IMC circuit primitive. For instance, DNN inference generally requires MVM to support the extensive weighted summation that takes place in each fully connected or convolutional layer. On the other hand, linear regression requires IMVM to support the pseudoinverse matrix calculation. Most importantly, each circuit/application combination may require a different set of properties of the RRAM device. The device requirements that should be fulfilled for each specific computing application are summarized in Table 2 and include multilevel operation, data retention, endurance, linear conductance update, linear conduction, and short-term memory.

**Table 2:**
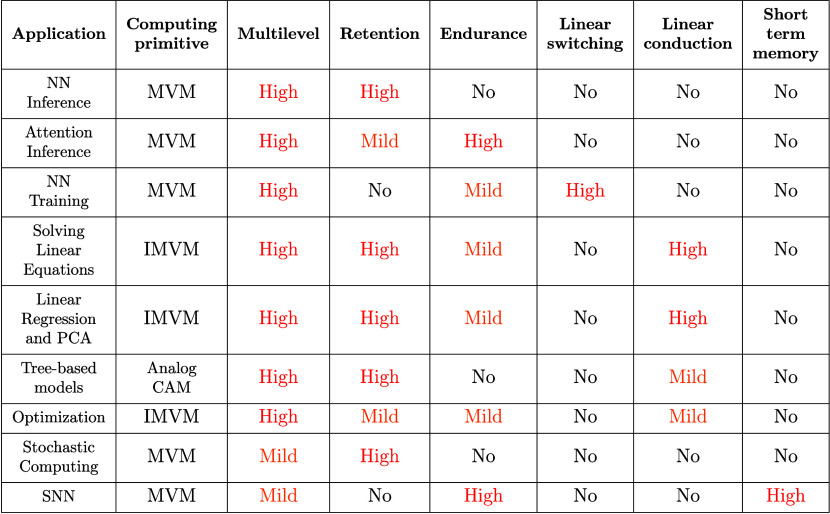
Summary of the Most Relevant Computing Applications and Corresponding RRAM Requirements

### 5.1 Neural Network Inference

The DPE circuit for MVM can be used for accelerating neural network inference.^165^ In fact, each layer of a feedforward DNN performs the dot product of the i-th layer neurons with their corresponding synaptic weights, to be accumulated at the i+1-th layer neurons as input for the nonlinear activation function. Thus, each layer of a DNN can be mapped to one or more CBAs, whose outputs are then accumulated and sent to an activation function unit. Recently, multiple fully integrated DNN accelerators have been realized using RRAM-based CBAs.^192–194^ Such accelerators generally include the CBA circuit, all of the sensing units required for operating it, and a bus or network-on-chip to control the data flow.

A notable example is NeuRRAM, consisting of 48 cores, each one of them able to realize multiplication of vectors with 256 × 256 stored matrices.^194^ Figure 21a illustrates the NeuRRAM circuit schematic of the core architecture. Each core consists of 16 × 16 corelets that share common bit-lines and word-lines along the rows and source-lines along the columns. Each corelet (Figure 21b) comprises a 16 × 16 RRAM CBA and one neuron circuit. CBAs in NeuRRAM can be connected in multiple configurations, including forward, where inputs are applied on the rows, for typical MVM, and backward, where input are applied on the column to perform the transposed MVM, which can be used for computing gradients during training.^172^ Compared to conventional DPE circuits performing MVM with current mode sensing (Figure 21c), NeuRRAM performs voltage mode sensing (Figure 21d) to reduce energy consumption. Accumulating the current of multiple devices can result in a large power consumption, thus requiring *large* TIAs. Also, as shown in Figure 21e, different DNN models, having different weight distributions, would result in strongly different current distributions to sense. By performing voltage mode sensing, the output is normalized by the total equivalent conductance seen by each neuron, resulting in a more uniform current distribution. Thanks to voltage-mode sensing, the energy-delay product in the NeuRRAM circuit outperforms other accelerators^195–198^ despite being designed in a relatively old technology, as shown in Figure 21f. To improve the classification accuracy during inference, NeuRRAM employs several hardware-software codesign techniques, such as noise-aware training and on-chip fine-tuning.

**Figure 21:**
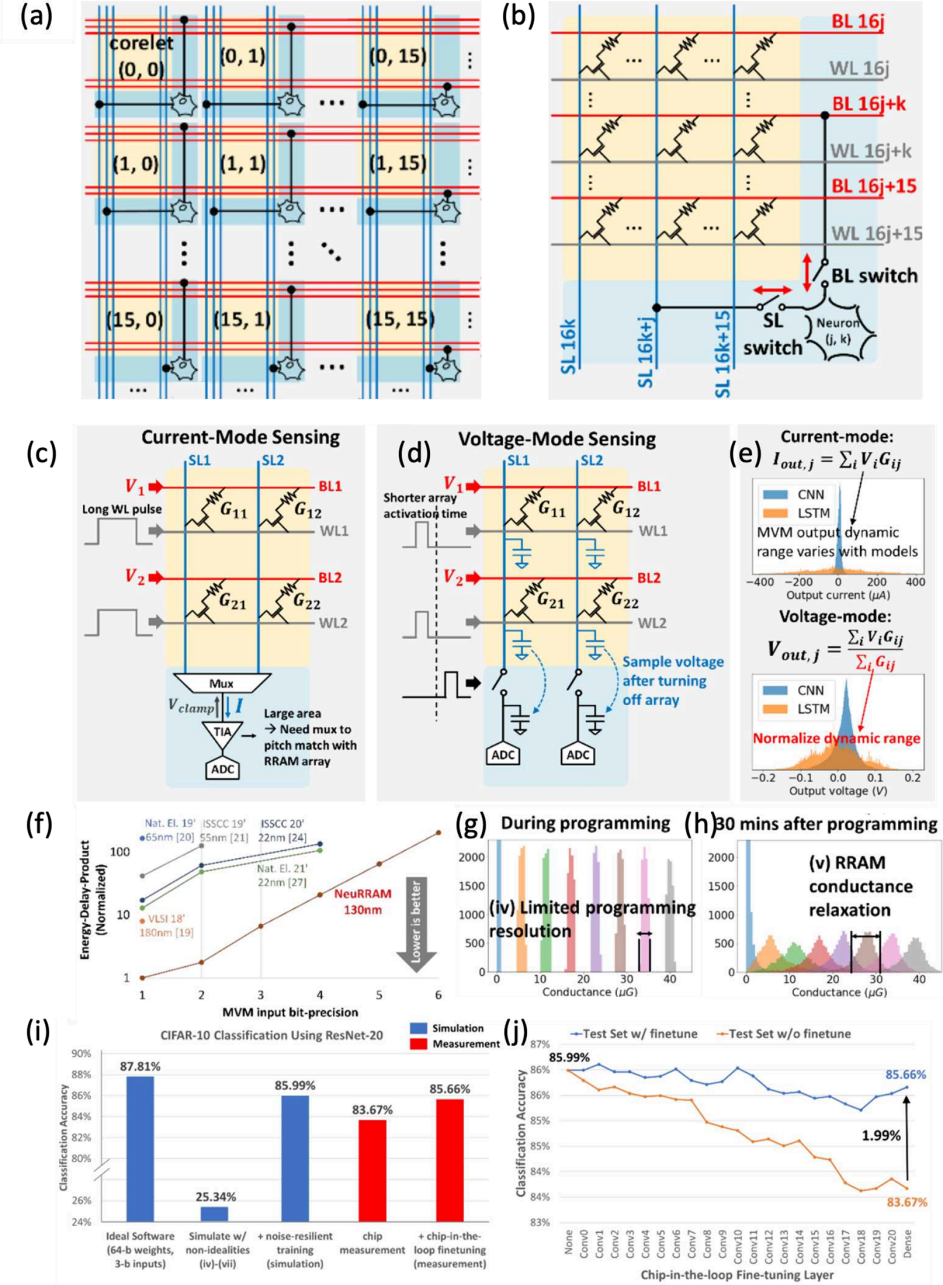
Illustration of DNN inference in NeuRRAM based on 1T1R arrays of RRAM devices. (a) Overall architecture and (b) individual corelet of the NeuRRAM chip. Each corelet of size 16 × 16 is connected to a neuron. 16 × 16 corelets are organized in a core, which has a resulting size of 256 × 256. (c) Illustration of current mode sensing and (d) voltage mode sensing. (e) Comparison of the distribution of output for current- and voltage-mode sensing. (f) Energy delay product as a function of bit-precision for multiple taped-out RRAM-based accelerators. (g) Distribution of programmed conductance during programming and (h) after 30 min for multiple programmed levels. (i) Classification accuracy for the data set of the Canadian Institute for Advanced Research with 10 classes (CIFAR-10) of NeuRRAM with various operation modes. (j) Layer-wise accuracy comparison with and without fine-tuning. Adapted from ref 192. Copyright 2022 Nature Publishing Group with Creative Commons Attribution 4.0 license http://creativecommons.org/licenses/by/4.0/.

Figure 21g shows the distribution of conductances programmed in 8 equally spaced levels, corresponding to 3 bits. The programming resolution is limited by several factors, including IR drops, capacitive coupling, and limited ADC range. Three bits are not enough to reach good classification accuracy, with most networks requiring 4 or even 8 bits for quantization for good enough results. Moreover, as shown in Figure 21h, after 30 min it is possible to observe a distribution broadening.^199^ To solve this issue, instead of quantizing a pretrained model or training a quantized network, which is equivalent to injecting uniformly distributed noise into weights, networks are trained with floating point precision with Gaussian distributed noise extracted from RRAM characterization. Figure 21i shows the simulated and experimentally verified result of such operation while performing inference on the CIFAR-10 data set, improving accuracy from 25.35% to 83.67%. Moreover, a fine-tuning training with chip-in-the-loop is performed to further increase the accuracy. Weights are programmed and finely adjusted in each layer while the network is undergoing training, to avoid multiple reprogramming while keeping hardware-awareness during the training operation. First, the model is trained completely offline. Then the first layer is programmed into the chip. Afterward, inference with the training set is performed by using experimental activations coming from the on-chip first layer and offline software activation for the other layers. The weights of all of the offline layers are adjusted to minimize the loss. The operation is repeated by programming the second layer on the chip, performing fine-tuning of all others, and so on until all of the model is programmed. The overall effect of such operation and noise injection is shown at the right-most bar in Figure 21i, resulting in an accuracy of 85.66%, with the layer-wise comparison between training with and without fine-tuning shown in Figure 21j.

A key advantage of using RRAM compared to traditional CMOS memories, such as SRAMs,^200^ for IMC is the multilevel programming capability, which results in improved computational efficiency per unit area and reduced complexity of the peripheral circuits. Multiple conductance states can be achieved by properly modulating the pulse parameters during the set operation or during a reset operation. Figure 22a shows the cumulative distribution function (CDF) of the conductance obtained after a set operation in a 1T1R RRAM device by changing the gate voltage of the select transistor at a fixed TE voltage, namely using an incremental gate pulse programming algorithm.^201^ Similarly, Figure 22b shows the CDF of conductance obtained after the reset operation, where the stop voltage was gradually increased during reset at a fixed gate voltage, which is referred to as the incremental reset pulse programming algorithm. These results demonstrate the ability to tune the average analog conductance via set or reset operations; however, the conductance variations are relatively large, which suggests that adoption of a closed-loop program-verify (PV) algorithm is advantageous.

**Figure 22:**
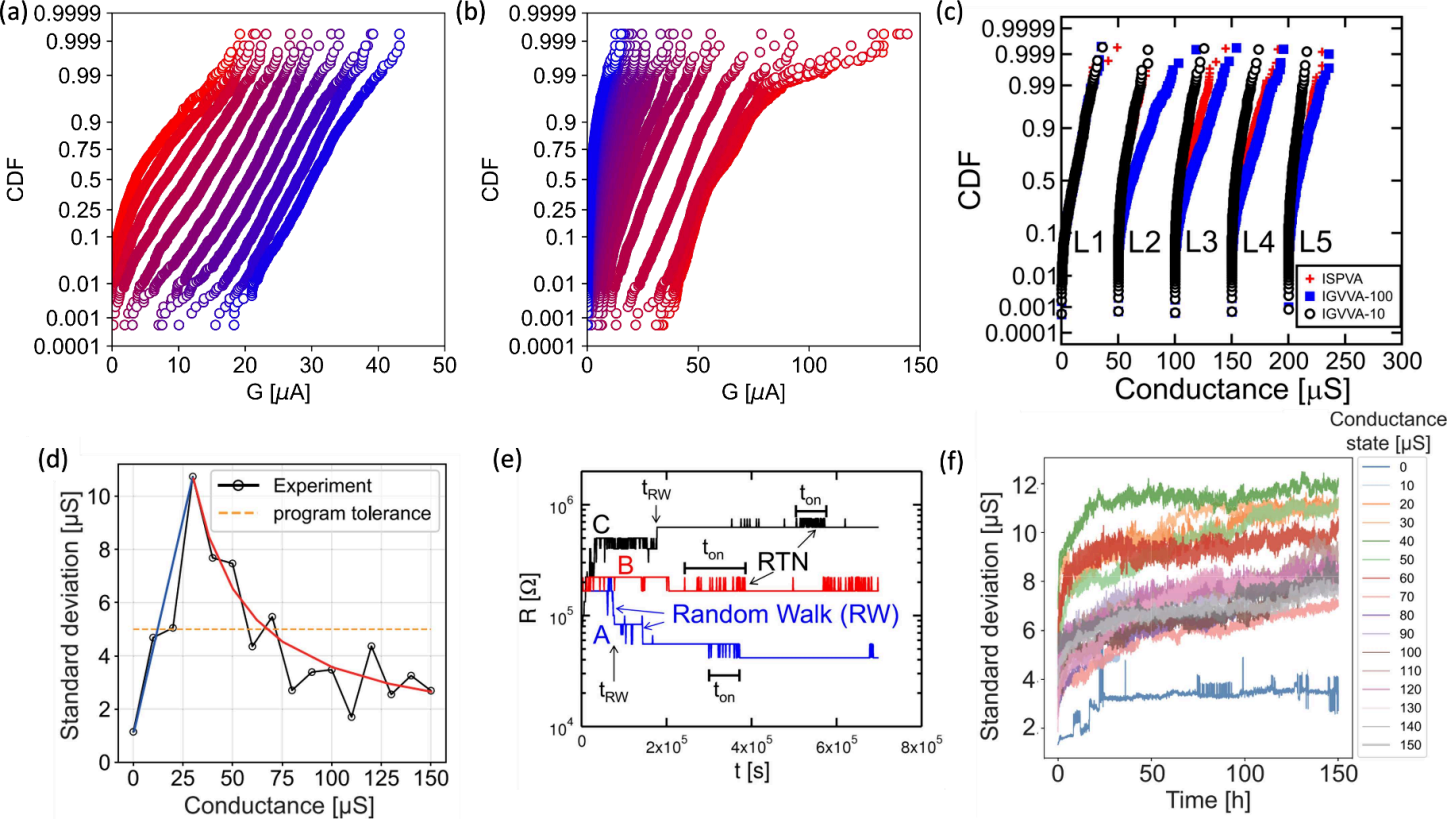
Multilevel programming of RRAM devices. (a) Cumulative distribution function (CDF) of the conductance obtained by modulating the set voltage. (b) Same as (a) but obtained by modulating the reset voltage. (c) CDF comparison for multiple program and verify algorithms. (d) Programming error standard deviation as a function of the median conductance. (e) Measured resistance as a function of time after programming, indicating fluctuations due to random walk (RW) and random telegraph noise (RTN). (f) Standard deviation of the programmed conductance as a function of time, indicating broadening of the programmed distributions. Panels (a,b) are adapted with permission from ref 200. Copyright 2021 IEEE. Panel (c) is adapted with permission from ref 201. Copyright 2021 IEEE. Panels (d,f) are adapted with permission from ref 202. Copyright 2023 IEEE. Panel (e) is adapted with permission from ref 203. Copyright 2015 IEEE.

Figure 22c shows the CDF of 5 conductance levels for different PV algorithms, namely incremental step pulse with verify algorithm (ISPVA)^202^ and incremental gate voltage with verify algorithm (IGVVA) based on 100 mV (IGVVA-100) and 10 mV (IGVVA-10) voltage steps.^203^ ISPVA consists of programming LRS in 5 different levels by keeping the gate voltage fixed at different levels, for example from 1 to 1.6 V with steps of 200 mV, and gradually increasing the TE voltage until the desired conductance is reached. In the case of IGVVA, the TE voltage is kept fixed, while the gate voltage is modulated with incremental steps until the desired conductance is reached. Results show that by using IGVVA with small steps of 10 mV, the conductance variation is significantly reduced. Hybrid PV algorithms with the modulation of both the TE and the gate voltages were also discussed^204^ and can achieve higher precision, although the hybrid algorithm might result in a relatively large number of pulses and, hence, a significant overhead. Figure 22d shows an example of the standard deviation of conductance as a function of conductance after a hybrid PV algorithm,^205^ showing essentially two regimes, one for HRS in which the standard deviation increases with the conductance and one for LRS, with an exponential decrease of the standard deviation with the conductance. Interestingly, the results indicate a trade-off between power consumption and precision.

Note that the multilevel operation is not a strict requirement for neural network training and inference, since, in principle, binary neural networks can be trained/inferred^206^ or multiple binary devices can be used to represent a multibit weight. However, without multilevel operation, RRAM-based accelerators might significantly underperform digital accelerators based on SRAMs. Typically, neural network layers require at least 4- to 8-bit precision,^207^ which is challenging to achieve with a single RRAM device. Multiple techniques for operating more than one device and increasing the precision have been presented. For instance, bit slicing is a technique where two or more devices are used to represent different slices of the weights, such as the two most significant and two least significant bits of a 4-bit weight.^208^ After the multiply operation, the output is reconstructed with a shift-and-add operation in the digital domain. Consider for example, a RRAM device that can efficiently store 4 levels, or equivalently 2 bits. If, for instance, *x*_10_ = 11 has to be stored (where the subscript 10 denotes the decimal base), two RRAMs can be programmed to the most and least significant values, *x*_4_^*M*^ = 2 and *x*_4_^*L*^ = 3. The overall value can be reconstructed as *x* = 4^1^ × 2 + 4^2^ × 3 = 11. Interestingly, doubling the number of RRAMs results in an exponential increase in the number of levels (or doubles the number of bits).

A different approach is to program the RRAM with a floating-point value and encode the resulting error in a second RRAM device.^209,210^ Given a target conductance *G* and a programmed conductance *G*_*p*_ resulting in an error *ϵ*_*G*_ = *G* – *G*_*p*_, a second RRAM is programmed with *G*_*ϵ*_ = *αϵ*_*G*_, where α is a scaling factor to match the maximum error on a CBA column with the full-scale range, corresponding at the highest conductance to program. Consider, for example, the case of a CBA column. Initially, a first weight *G*_00_^*W*^ is programmed in a first, most significant, RRAM cell. After PV, the resulting conductance is *G*_00_^*M*^ = *G*_00_^*W*^ – *G*_00_^*Mϵ*^ where *G*_00_^*Mϵ*^ is the error resulting from the imprecise programming operation. The operation is repeated until all columns are programmed. At this point, the scaling factor of the column is calculated as

5 $$\alpha_{0}=\frac{\max_{i}\left(G_{i0}^{M\epsilon }\right)}{G_{FSR}}$$

where max_*i*_ (*G*_*i*0_^*Mϵ*^) is the maximum error across the column and *G*_*FSR*_ is the full-scale range of the RRAM conductance, namely *G*_*FSR*_ = *G*_max_ – *G*_min_. At this point, for the first weight, a second, least significant, RRAM device is programmed with the error conductance, namely:

6 $$G_{00}^{e}=\alpha_{0}^{-1}G_{00}^{M\epsilon }$$

where α_0_ is used as the scaling factor. After PV is performed, the programmed conductance is *G*_00_^*L*^ = G_00_^*e*^ – *G*_00_^*Lϵ*^. The scaling factor α_0_ is thus used as the attenuation of the second RRAM column. The resulting equivalent conductance of the most and least significant couple is thus given by

7 $$G_{00}^{eq}=G_{00}^{W}-G_{00}^{M\epsilon }+\alpha_{0}G_{00}^{L}=G_{00}^{W}-G_{00}^{M\epsilon }+\alpha_{0}\left(G_{00}^{e}-G_{00}^{L\epsilon }\right)=G_{00}^{W}-G_{00}^{M\epsilon }+\alpha_{0}\left(\alpha_{0}^{-1}G_{00}^{M\epsilon }-G_{00}^{L\epsilon }\right)=G_{00}^{W}-\alpha_{0}G_{00}^{L\epsilon }$$

The error is thus reduced directly by a factor of α_0_, given that α_0_ is generally much smaller than 1.

In other computing primitives, such as CBAs with analog feedback for scientific computing (see Section 4.2) and Ising machines with continuous time and continuous variables, the presented slicing techniques can hardly be adopted without a significant overhead. Similarly, for analog CAMs (see Section 4.3), doubling the number of cells results in doubling the number of levels, rather than the number of bits, which results in an exponential overhead necessary to increase the precision.^211^

In addition to the limited precision of programming multilevel conductances, RRAM devices can undergo significant conductance changes after programming. Figure 22e shows various two possible sources of conductance change after programming, namely random walk (RW) and random telegraph noise (RTN).^212^ RW is an abrupt change of conductance at a random time and amplitude, while RTN consists of a two-level fluctuation of the conductance. Figure 22f shows the standard deviation of conductance as a function of time for multiple conductance states of TaO_*x*_ RRAM devices.^205^ For most analog computing applications, conductance stability, or retention, is a strict requirement. In the case of DNNs inference, knowledge about conductance noise and broadening can be embedded in the training algorithm. For instance, DNN model training optimization and fine-tuning for noise-resiliency have demonstrated an increase of accuracy by 8.4% for inference of the MNIST data set.^205^ A similar approach has shown significant improvement in the inference of decision trees in analog CAMs.^213^ Nevertheless, sometimes RRAMs experience abrupt changes that can significantly debilitate the accuracy performance. For some applications, such as linear algebra, it is not possible to *train* the problem with noise awareness. For such cases, the analog error correction code (A-ECC) has been presented.^214,215^ Digital error correction codes (ECC) are ubiquitous both in memory and communication systems^216^ and use special features of the Boolean alphabet to recognize if an error occurs and eventually decode the correct output. In the case of A-ECC, the alphabet is considered an integer value, but the techniques can still be applied. By equipping the RRAM arrays with the required redundancy, it is possible to compute a *syndrome* which can determine if the result is correct by its parity. In case the computed dot product is wrong, a decoder matrix can be used to correct the wrong output. Experiments have shown accuracy recovery from 73.12% to 97.36% for the inference classification of the MNIST data set.^217^

### 5.2 Inference in Attention-Based Models

With the growing interest in LLMs and their increasingly high energy consumption, LLM hardware accelerators based on RRAM have started to emerge. However, performing inference of LLMs and other attention-based models^218^ raises novel challenges to IMC accelerators. Figure 23 shows the differences in performing inference of conventional DNNs or recurrent neural networks compared with attention-based ones. The programming operation is highlighted by the red arrows in the figure, while the inference (or read) operation is represented with black arrows. In conventional DNNs (Figure 23a), the inference operation in the *i*-th layer consists of two steps: (1) an MVM between the input *x* and the static weight matrix *W*_*i*_ and (2) passing the output of the multiplication through an activation function and sending the result to the next layer. To accelerate the inference operation, the weights *G*_*W*_*i*__ of the *i*-th layer are programmed in the CBA only once (Figure 23b) and then reused for the whole lifetime of the model. As a result, inference can be viewed as a mere read operation on the CBA (Figure 23c).

**Figure 23:**
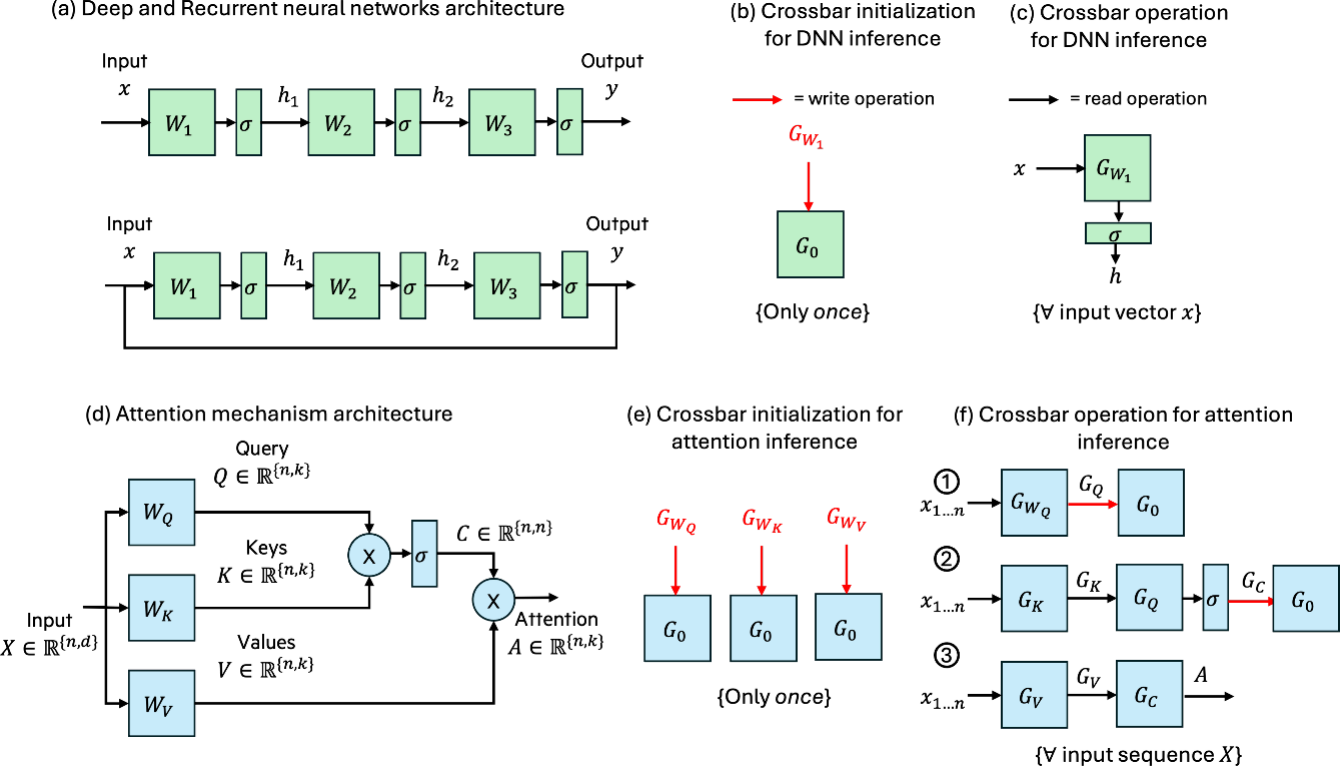
Architecture of a multihead attention unit in a transformer. (a) DNN structure and pipeline. (b) Programming and (c) performing inference of a DNN in a CBA. (d) Attention-based mechanism in transformers. (e) Programming and (f) performing inference of an attention layer in CBAs.

Transformer models, which are the foundational models for LLMs, are essentially heterogeneous neural network models that differ from conventional DNNs by the attention mechanism, which is capable of assessing the relationship between different tokens in the input sequence. Figure 23d illustrates the attention mechanism architecture. An input sequence *X* is multiplied by three matrices (*W*_*Q*_, *W*_*K*_, *W*_*V*_) generating the query (*Q*), key (*K*), and value (*V*) matrices, respectively. The final attention matrix *A* is defined as

8 $$A=softmax\left(\frac{QK^{T}}{\sqrt{d}}\right)V$$

where the softmax function is given by $softmax\left(z_{i}\right)=\frac{e^{z_{i}}}{\overset{K}{\underset{j=1}{\sum }}e^{z_{j}}}$ , $\sqrt{d}$ is a normalization factor and *d* is the size of the Q, K and V matrices. Note that all of the matrix multiplications in eq 8 contain dynamic values that change at each new inference sequence. After initializing the CBAs with the query, key, and value weights (Figure 23e), an inference pipeline can be built by subsequent CBA read and write. A possible example of a three-step pipeline is shown in Figure 23f. In the first step, *Q* is computed by performing an MVM with input sequence *X* and query weights *G*_*W*_*Q*__. At the same time, *K* is computed by performing an MVM with input sequence *X* and query weights *G*_*W*_*K*__. A similar operation is performed for computing *V* as well. Then, in the second step, *Q* is written to a new CBA (*G*_*Q*_) and multiplied by *K*. The product *Q* × *K* ^*T*^ is then passed through the activation function σ (representing the *softmax*), normalized, and programmed into a CBA *G*_*C*_. Finally, attention output *A* is obtained in step 3, where *V* is multiplied by *G*_*C*_.

Notably, the important difference between inference in DNNs and attention-based neural networks is that weights are static in the former case, while matrices are dynamic in the latter case and thus must be programmed in the CBA at each inference step, which dramatically reduces the performance, energy efficiency, and reliability of IMC accelerators. Accurately programming the matrix entries into RRAM CBA devices requires several iterations, thus limiting the inference speed and resulting in a significant increase in the latency. Also, given the limited endurance of RRAM devices, the lifetime of the accelerator might be significantly reduced. Assuming an optimistic endurance of 10^8^ cycles, an open-loop single-pulse programming operation^219^ and a 1 ms latency for inference of a language model,^220^ we can anticipate that the chip would die after only 10^5^ seconds, which is equivalent to approximately 1 day of operation.

From the previous analysis, the implementation of transformer accelerators with RRAM-based IMC circuits might be severely affected by endurance from the reliability viewpoint. Cycling endurance is an essential requirement not only for inference in transformer accelerators but also for computing applications leveraging the weight update as a basis for learning, such as neural network training and spiking neural networks where RRAM are used in I&F neurons and STDP synapses. For memory applications, endurance would support RRAM for reconfigurable NVM where data need to be continuously updated.

Endurance is generally evaluated as the number of set/reset cycles for which the RRAM device continues to display a minimum resistance window. For instance, Figure 24a shows the measured resistance after set and after reset for a HfO_2_-based RRAM device as a function of the number of cycles *N*_*C*_.^221^ The resistance window slightly increases with an increasing number of cycles thanks to a gradual lowering of the LRS resistance. However, after about 2.6 × 10^4^ cycles, the device displays a sudden decrease of the resistance window, where both the LRS and HRS resistances converge to an intermediate value. Endurance strongly depends on the reset condition, namely, the stop voltage *V*_*stop*_ at which the reset operation was conducted. Figure 24b shows the endurance cycles *N*_*C*_ as a function of *V*_*stop*_, showing a steep exponential decrease of *N*_*C*_ for increasing *V*_*stop*_.^63^ For relatively small *V*_*stop*_, the reset transition could not take place, thus leaving the device in a permanent (stuck) set state. The figure also shows data for various values of *I*_*C*_, which has a negligible role in controlling endurance. The fundamental mechanism for endurance failure was attributed to negative set, namely a dielectric breakdown effect taking place during the reset operation under negative voltage.^63^ Given the lack of a current limitation during the reset operation, a negative set can lead to uncontrolled filament growth. Even after reset, the resulting filamentary region shows a relatively low resistance, which accounts for the intermediate value after endurance failure in Figure 24a. Note that *V*_*stop*_ also controls the resistance window, as the HRS resistance increases with *V*_*stop*_.^65^ These results thus indicate that there is an inherent trade-off between endurance and the resistance window, where an increasing resistance window generally comes at the expense of a smaller endurance. This general trend was also observed by comparing different device stacks and materials, as shown in Figure 24c.^222^ To improve the cycling endurance, the bottom electrode was optimized by selecting inert materials, such as C,^65^ Ir,^124^ Pt,^223^ and Ru,^223,224^ resulting in relatively large endurance in the range of 10^12^ cycles.^225^ When the statistical tails of early failing bits are included, this endurance is not sufficient for the intensive cycling required by transformer accelerators and some neural network training applications. For these applications, CMOS volatile memory devices, such as SRAM and DRAM, seem to be most adequate.

**Figure 24:**
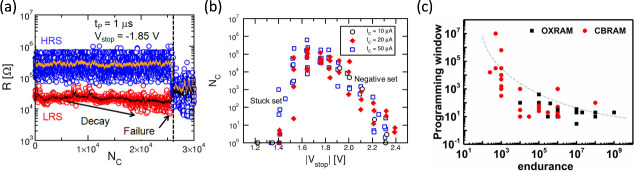
RRAM endurance. (a) Measured LRS and HRS resistances as a function of the number of cycles for a HfO_2_-based RRAM device, indicating an endurance of about 2.6 × 10^4^ cycles. (b) Endurance as a function of *V*_*stop*_, namely the maximum negative voltage in the reset pulse. Endurance exponentially decreases with *V*_*stop*_ due to the negative set effect. (c) Correlation between endurance and resistance window for oxide-based RRAM and CBRAM. Panel (a) is adapted with permission from ref 221. Copyright 2014 IEEE. Panel (b) is adapted with permission from ref 63. Copyright 2015 IEEE. Panel (c) is adapted with permission from ref 222. Copyright 2016 IEEE.

### 5.3 Neural Network Training

DNN training generally combines two major workloads, namely, forward propagation, to compute the error of the DNN, and backward propagation, or simply backpropagation, to enable the gradient descent to minimize the error function. While the first operation is similar to inference, the second one requires additional linear algebra operations.^226^ From the execution viewpoint, the radical difference between training and inference is that weights remain fixed for inference operation, while they need to be continuously updated during training at each epoch step, which thus requires extensive dynamic IMC as opposed to static IMC for DNN inference. Consider for example Figure 25a, which shows the last three layers of indices *i*, *j* and *k*, respectively, of a multilayer perceptron (MLP). Training consists of three main functions, namely (i) forward pass for computing activations, (ii) gradients computation, and (iii) weight update. Assuming an input vector *y*_*i*_ at the *i*-th layer of a neural network with weights *W*_*ij*_ between layer *i* and layer *j*, the output vector of the layer *z*_*j*_ is given by

9 $$z_{j}=W_{ij}y_{j}$$

which must be submitted to a nonlinear activation function such as a sigmoid or a rectifying linear unit (ReLU). Equation 9 can be readily mapped in a CBA as a conventional MVM reported in Section 4.1, while the activation function is usually implemented in a dedicated analog or a digital circuit,^208^ as shown in Figure 25b. Training the MLP involves back-propagation to compute the gradients of the loss function, with respect to the weights and biases. Gradients can be computed by deriving the loss function L , which generally consists of the mean squared error for regression or the cross-entropy for classification, both assessing the error between the predicted output of the last layer *y*_*k*_ and the true output *ŷ* provided by the label. Let *δ*_*k*_ = *y*_*k*_ – *ŷ* be the error term for the output layer *k*. After computing the error for the last layer, it is possible to backpropagate it to any other layer via the chain rule. For the layer *j*, the error term can be computed as

10 $$\delta_{j}=\left(W_{jk}^{T}\delta_{k}\right)⊙\sigma^{\prime }\left(z_{j}\right)$$

where σ′ is the derivative of the activation function and ⊙ is the element-wise product which is generally computed in the digital domain. Equation 10 includes a transposed MVM, or M^*T*^VM, that can be implemented by applying the inputs to the columns, rather than the rows, of a CBA as shown in Figure 25c. Finally, the weights can be updated according to the gradient descent rule as *W*_*ij*_ ← *W*_*ij*_ – *ηδ*_*j*_, where η is the learning rate. To maximize the energy efficiency for weight update, one can implement the outer-product accumulate (OPA) in the CBA to update the conductance in the direction of the gradients as shown in Figure 25c.^226,227^ The OPA approach consists of applying at the same time the input vector and the weight update vector *ηδ*_*j*_ to the CBA rows and columns, respectively.

**Figure 25:**
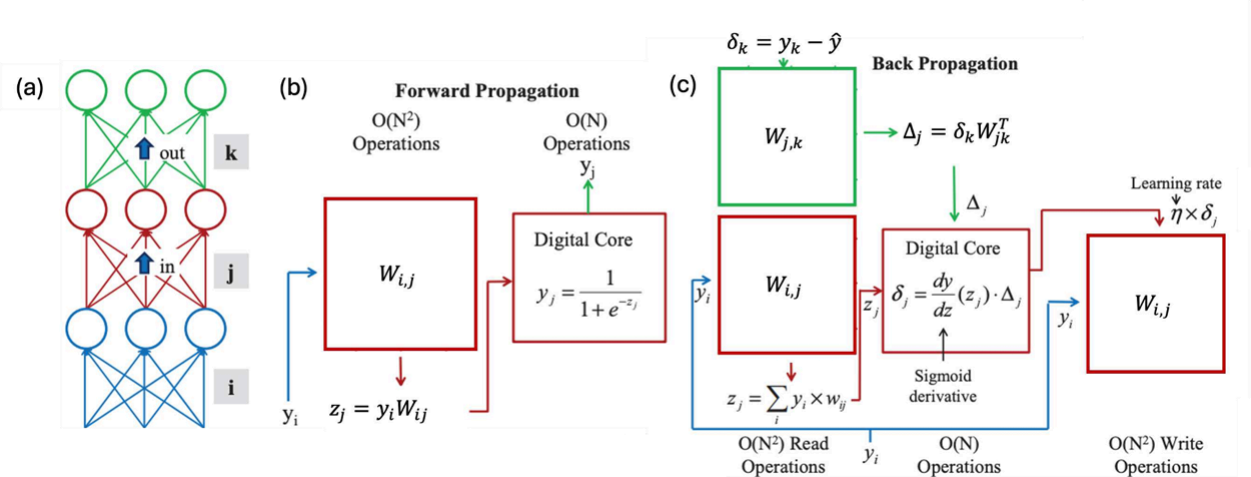
Illustration of neural network training. (a) Example of feedforward neural network. (b) Mapping of the forward propagation on CBA. (c) Mapping backpropagation in CBAs. Adapted with permission from ref 226. Copyright 2016 IEEE.

However, significant device challenges arise during training, notably the nonlinearity and asymmetry of weight updates. The gradual set and reset operations in RRAM devices are inherently nonlinear and asymmetric.^226^ The conductance change generally depends on the conductance state; e.g., a positive voltage pulse might result in a strong increase in conductance for low conductance, while the same pulse might cause a minimum change at high conductance. Also, a positive pulse might cause a small change of conductance, while a negative pulse of the same amplitude and at the same initial conductance state might result in a large change of conductance.^228^

It is important to note that the use of PV algorithms is impractical during training, as the programming operation must be rapid to expedite the overall training process, while PV would introduce an unacceptable energy/latency cost. Moreover, to maximize efficiency, it is not feasible to read each conductance state before updating; therefore, blind update pulses should be applied irrespective of the conductance state.

During blind updates, the asymmetric and nonlinear conductance as a function of the normalized update pulse number *p* can be described by^226,228^

11 $$G=G_{0}\left(1-e^{-\nu p}\right)+G_{\min}$$

where *G*_0_ is a constant given by *G*_0_ = (*G*_max_ – *G*_min_)/(1 – *e* ^–ν^), while *G*_min_ and *G*_max_ represent the minimum and maximum conductance, respectively. The exponent ν in eq 11 is a shape factor, or nonlinearity parameter, which provides a metric for the nonlinearity of the weight-update characteristic. Similarly, the conductance as a function of the negative update pulse number can be modeled by

12 $$G=G_{\max}-G_{0}\left(1-e^{-\nu \left(1-p\right)}\right)$$

where the same shape factor ν has been assumed for simplicity, although the nonlinearity might depend on the voltage polarity. Figure 26a shows the calculated conductance as a function of the pulse number for positive and negative change updates and various shape factors ν.

**Figure 26:**
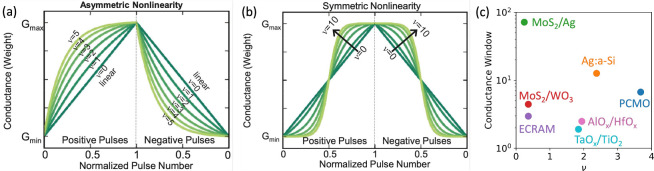
Illustration of the weight update during training. (a) Conductance as a function of the number of update pulses for multiple shape factors ν for asymmetric characteristics. (b) Same as (a), but for symmetric characteristics. (c) Conductance windows as a function of ν for multiple device technologies. Panels (a,b) are adapted with permission from ref 226. Copyright 2016 IEEE. Panel (c) is adapted with permission from ref 228. Copyright 2022 IEEE.

Some devices,^229^ while showing a symmetric switching between positive and negative pulses, might display a nonlinear operation within the switching polarity. In that case, the conductance as a function of the pulse update can be modeled as^226^

13 $$G=\frac{A}{1+\exp\left(-2\nu \left(p-0.5\right)\right)}+B$$

where parameters *A* and *B* are given by

14 $$A=\left(G_{\max}-G_{\min}\right)\frac{e^{\nu }+1}{e^{\nu }-1}$$

15 $$B=G_{\min}-\frac{G_{\max}-G_{\min}}{e^{\nu }-1}$$

Figure 26b shows calculations from eq 13 as a function of the update pulse number for various shape factors ν.

The memory device technology can be engineered to achieve a low ν to optimize the update linearity. Ideally, a suitable device for training should display both a large conductance window, allowing for multiple updates without the need for refreshing the overall weights, and a low ν. Figure 26c shows the conductance window as a function of ν for multiple resistive memory device technologies,^230^ including RRAM with different stacks, such as AlO_*x*_/HfO_*x*_,^231^ Ag:a-Si,^232^ TaO_*x*_/TIO_2_,^57^ and Pr_1–*x*_Ca_*x*_MnO_3_ (PCMO).^233^ The figure also shows non-RRAM device technologies, such as MoS_2_/Ag charge-trap memory (CTM),^230^ MoS_2_/WO_3_ CTM,^97^ and WO_3_-based electrochemical random access memory (ECRAM).^234^

Note that nonlinearity and asymmetry affect the training by changing the weight update characteristics depending on the conductance state. As a result, the hardware-operated weight update *W*_*ij*_ ← *W*_*ij*_ – *ηδ*_*j*_ features an error, compared to the theoretical gradient descent behavior, due to the device asymmetry and nonlinear update operation. To compensate for such errors, a possible solution is to implement a dedicated device-aware training algorithm, such as the Tiki-Taka methodology.^235^ The Tiki-Taka algorithm mitigates update errors by adopting a coupled dynamical system that minimizes both the original objective function and the unintended cost term due to device asymmetry at the same time. According to the Tiki-Taka algorithm, the weight matrix *W* is first split into two matrices *A* and *C* such that

16 W=γA+C

where γ is a convenient scaling factor. Then, as a one-time initial calibration, a symmetry point shifting technique is used to eliminate the asymmetry term in the weight update in matrix *A*. The symmetry point shifting technique consists of a sequence of alternating positive and negative update pulses which is applied to all devices in the CBA in parallel. Such a sequence is designed to enable the convergence of the device conductance to its symmetry point, namely, the conductance value where the conductance increment Δ*g*_*ij*_^+^ and decrement Δ*g*_*ij*_^–^ display the same amplitude. During training, the updates are accumulated on *A*, which exhibits symmetric behavior around zero, and are periodically transferred to *C*, ensuring the network weights converge to the optimal values despite device asymmetry. Simulation results show that the Tiki-Taka algorithm achieves a training accuracy that is comparable to that of ideal symmetric devices.^235^

Recently a new RRAM device was developed for Tiki-Taka training algorithm requirements, by adopting a stack of TiN/conductive-TaO_*x*_/HfO_2_/TiN structure which enables relatively low voltage and low current operation, as well as symmetric potentiation/depression characteristics.^62^ Figure 27a shows the RRAM device characteristics for repeated potentiation and depression sequences with equal amplitude of updating pulses. The characterization technique consists of (i) the application of several voltage pulses of opposite polarity followed by (ii) the application of a sequence of alternated single pulses of positive and negative amplitude to reach the symmetry point. The number of states *N*_*states*_ is defined as

17 $$N_{states}=\frac{G_{\max}-G_{\min}}{\overset{®}{\Delta G_{SP}}}$$

where *G*_max_ and *G*_min_ are the maximum and minimum conductance averaged over 10 cycles, respectively, while ( $\overset{®}{\Delta G_{SP}}$ is the standard deviation of the conductance during the final phase of single up/down pulse alternation (Figure 27a). The symmetry point skew *SP*_*skew*_, which should be ideally equal to 50%, was evaluated as

18 $$SP_{skew}=\frac{G_{\max}-\overset{®}{G_{SP}\;}}{G_{\max}-G_{\min}}$$

where $\overset{®}{G_{SP}\;}$ is the average conductance during the single up/down train (Figure 27b). Finally, the noise-to-signal ratio *NSR* is computed as

19 $$NSR=\frac{\sigma_{G,SP}}{\overset{®}{\Delta G_{SP}}}$$

which provides the relative standard deviation of conductance updates during the single up/down pulse train and ideally should be much smaller than one. Experimental results for multiple devices demonstrate median values of *N*_*states*_ = 22, *SP*_*skew*_ = 54% and generally *NSR* < 1, with a trade-off between *NSR* and *N*_*states*_. These results demonstrate the feasibility of the proposed RRAM device for Tiki-Taka training, with only a 0.7% drop in accuracy compared with digital hardware in training a three-layer network for MNIST classification.^62^ In addition to device engineering, the Tiki-Taka algorithm can be improved to mitigate device nonidealities, such as a high robustness against device noise.^236^

**Figure 27:**
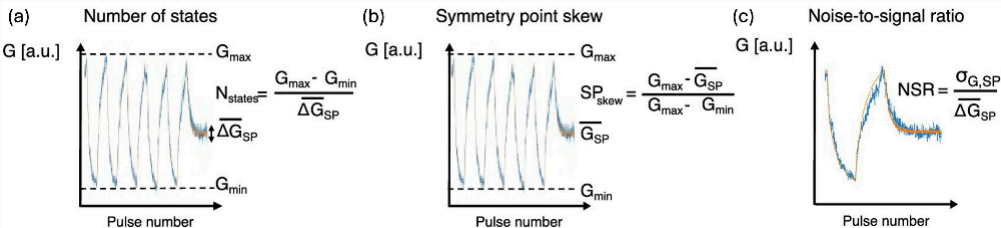
(a) Number of states, (b) symmetry point skew, and (c) noise to signal ratio as defined by the Tiki-Taka Algorithm. Reprinted from ref 62. Copyright 2024 ACS.

### 5.4 Other Machine-Learning Applications

The mainstream application of RRAM-based IMC accelerators is indeed the inference and training for deep learning and neural network primitives. However, other machine learning models such as regression, principal component analysis (PCA), and tree-based machine learning are characterized by higher robustness and explainability, and thus are preferred in sectors like healthcare, finance, and law, where decisions can significantly impact individuals’ lives.^237^ These models differ significantly in workload compared to neural networks, thus making it challenging for accelerators specialized for deep learning to efficiently run, e.g., a tree-based model. However, circuit primitives of Section 4 can provide a useful platform for various types of RRAM-based accelerators tailored to these established machine-learning algorithms. In the following, IMC implementations of linear regression, PCA, and tree-based machine learning with RRAM-based accelerators are reviewed.

Linear regression is a widely used machine learning model with applications across various domains, including biology, social science, and economics.^238^ In a simple one-dimensional case, linear regression requires finding the line that best fits a set of data by minimizing a certain error. Solving a one-dimensional linear regression thus consists of finding the intercept and slope of the best-fitting line. More generally, linear regression involves finding the *M*-dimensional vector *w* in the overdetermined linear system:

20 Xw=y

where *X* is an *N* × *M* matrix representing the input data and *y* is an known *N*-dimensional vector. A possible way to solve eq 20 is to minimize the least-squares error (LSE) given by ∥ϵ∥_2_ = ∥*Xw* – *y*∥_2_ by computing the Moore–Penrose inverse (or pseudoinverse) matrix *X* ^+^ given by^239^

21 $$w=X^{+}y={\left(X^{T}X\right)}^{-1}X^{T}y$$

The equation can be solved in an IMC circuit obtained by a proper configuration of MVM (Section 4.1) and IMVM circuits (Section 4.2).^178^ Figure 28a shows the IMVM circuit consisting of two CBAs for computing linear regression through eq 21. A first CBA with conductance *G*_*X*_ is used to map the independent variables *X*, and a current *I* is used to inject the dependent variable *y* on the CBA rows, which are kept at virtual ground. The CBA rows are connected to the low-impedance input of a TIA stage with a *G*_*TI*_^‑1^ gain, and its output is connected to the second CBA rows, which map again *G*_*X*_. The second CBA columns are kept at the virtual ground via operational amplifiers, whose output is then connected to the first CBA columns.

**Figure 28:**
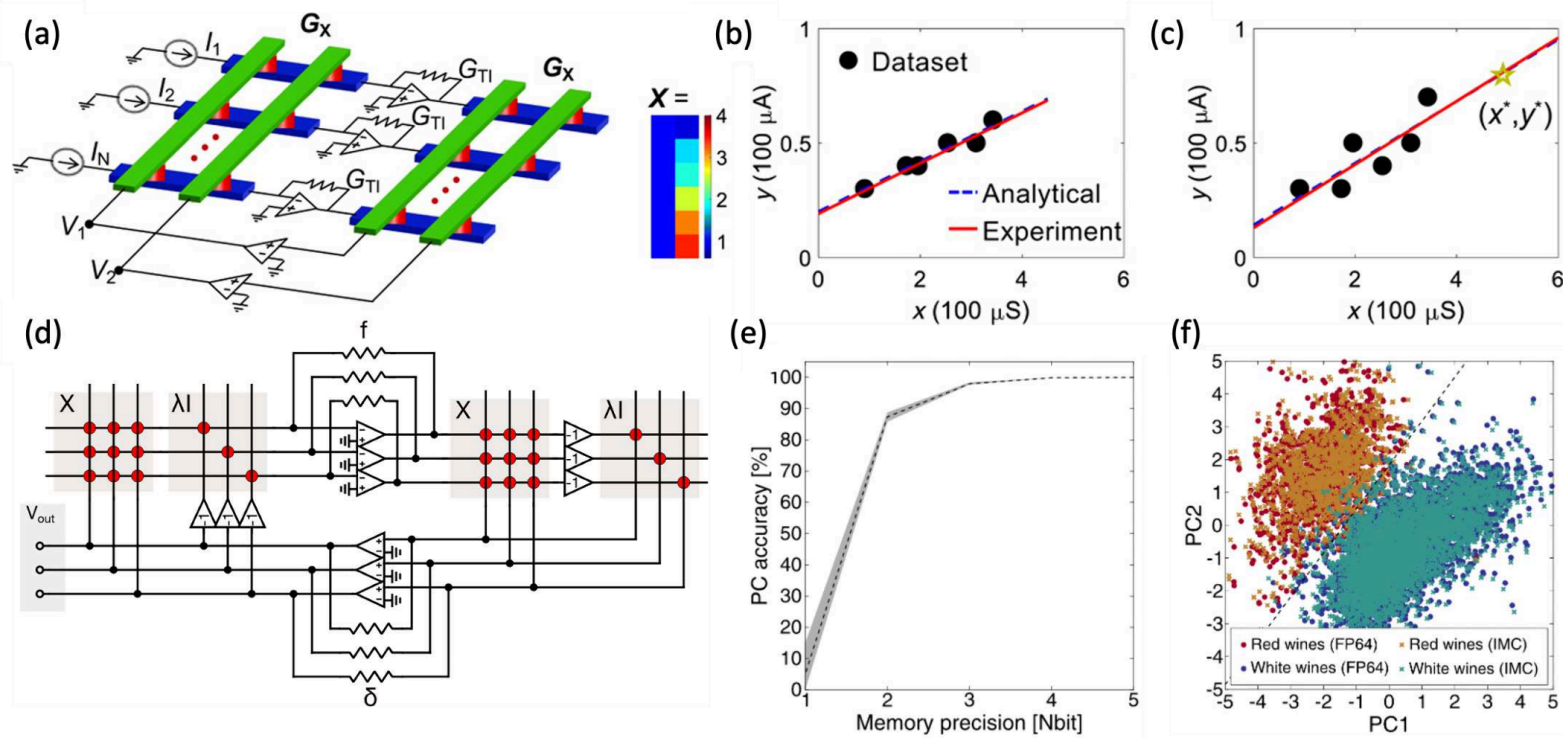
Illustration of IMC Implementations of Regression and PCA. (a) CBA circuit with closed-loop analog feedback for linear regression computation. (b) Experimental demonstration of linear regression and (c) prediction using the circuit in (a). (d) CBAs with closed-loop analog feedback for PCA computation. (e) PCA accuracy as a function of the memory device precision. (f) Comparison between the data set projections computed by either 64-bit floating-point (FP64) precision or limited precision (IMC). Panels (a,b,c) are adapted from ref 178. Copyright 2024 American Association for the Advancement of Science with Creative Commons Attribution 4.0 license https://creativecommons.org/licenses/by-nc/4.0/. Panels (d,e,f) are adapted with permission from ref 237. Copyright 2023 IEEE.

The input currents in the TIA stage are then given by *I*_*T*_ = *G*_*X*_ *V* + *I*, where *V* is the output voltage of the high impedance amplification stage. The current *i*_*T*_ is then divided by the TIA feedback conductance as *V*_0_ = – *I*_*T*_/*G*_*TI*_. The high impedance stage forces the current flowing in the second CBA columns to be zero, which leads to

22 $$I_{0}=G_{X}^{T}V_{0}=-G_{x}^{T}\frac{I_{T}}{G_{TIA}}=-G_{x}^{T}\frac{G_{x}V+I}{G_{TIA}}=0$$

By solving this equation, it is possible to obtain *V* = −(*G* ^*T*^ *G*)^−1^ × *G* ^*T*^ *i*, which, apart from the sign, provides the solution to the linear regression problem of eq 20 as the voltage at the columns of the left array in Figure 28a. Figure 28b shows an experimental verification of the circuit, where the experimentally obtained linear regression closely matches the analytical result. Finally, by programming an extra row with a new independent variable and keeping the input floating, it is possible to predict its regression by measuring the current flowing into it. Figure 28c correspondingly shows an experimental result, where the extra row was programmed with values of x*, allowing extraction of the predicted value y*.

The concept of combining multiple CBAs in a feedback loop to solve complicated algebraic equations can be further extended. An example of combining multiple CBAs and feedback circuits for solving multiterm equations is shown in Figure 28d for the case of principal component analysis (PCA). PCA is a technique aimed at reducing the dimensionality of a data set by computing the vector directions along which the data set variance is maximized.^240^ In particular, the *i*-th principal component corresponds to the *i*-th eigenvector of the data set covariance matrix *C* = *m* ^–1^ *D* ^*T*^ *D*, where *D* is a matrix representing the data set and *m* the number of observations, namely the number of *D* rows. To reduce the dimensionality of the data set, only the PCs with eigenvalues larger than a threshold are used to create a basis *P* and a corresponding data set projection *Y* = *DP*. The principal eigenvector can be obtained by the circuit in Figure 18a modified by the removal of the external input signal generators, since the known term is zero in calculating the eigenvectors.^241^ However, for computing any eigenvector starting from its eigenvalue, a more complicated circuit is needed. Figure 28d shows the closed loop analog feedback circuit for the eigenvectors computation.^242^ Here, given a known matrix *X* and a tentative value for its eigenvalue λ, matrix *X* – *λI* is programmed in a total of four separate CBAs, interleaved by two stages of TIAs in two opposite directions, with gains *f* and δ, respectively. By following a similar approach as in the regression example, it is possible to compute the output of the operational amplifier as

23 $$V_{out}=\delta^{-1}{\left(X-\lambda I\right)}^{T}f^{-1}\left(X-\lambda I\right)V_{out}$$

which leads to

24 $$\left({\left(X-\lambda I\right)}^{T}\left(X-\lambda I\right)-f\delta I\right)V_{out}=0$$

For *fδ* approaching zero, eq 24 can be approximated to (*X* – *λI*)*V*_*out*_ = 0, where *V*_*out*_ thus provides an estimation of the eigenvector. Finally, by replacing *X* with a matrix–matrix multiply circuit for computing *C* = *m* ^–1^ *D* ^*T*^ *D*, namely two CBAs mapping *D* interleaved by a TIA, all the operations of the PCA can be computed in the analog domain in one step.

Figure 28b shows the accuracy in computing the principal components (PC) as a function of the number of bits for representing the matrix values, namely, the RRAM memory bit precision, demonstrating the need for at least 4 bits for reaching good results. A graphical example is shown in Figure 28c comparing the two data set projections obtained by using 64-bit floating-point (FP64) precision on a digital processor and integer 4-bit precision using the proposed analog IMC circuit, demonstrating a good agreement. Results show that the IMC approaches significantly provide better performance than a graphical processing unit (GPU), having similar accuracy and throughput but up to 10000× improved energy efficiency.

Another approach to infer traditional machine learning models with IMC is the use of an analog CAM for accelerating tree-based machine learning.^189^ Decision trees are well-established machine learning models that are highly appreciated by the machine learning community thanks to their easiness to train, state-of-the-art performance for tabular and time-series data,^243^ and explainable decision process.^244^

Figure 29a shows an example of a decision tree with four input features, three nodes, a depth *D* = 2, and *L* = 4 leaves. A node consists of a comparison of one of the input features with a trainable value resulting in a branching operation; for example, the first node performs the comparison *f*_1_ ≤ 0.2 for the first feature with the trained value 0.2 taking either the left branch in case the condition is met or the right branch if the condition is not met. Conditional branching is performed until reaching the leaf which stores the model prediction, such as the predicted class. Ensembles of decision trees such as Random Forests^245^ and eXtreme Gradient Boosting (XGBoost)^246^ consist of multiple trees inferred in parallel and trained either by bagging, i.e., each tree is trained on a subsample of the data set, or boosting, i.e. the i-th tree is trained based on the errors of the i-1-th tree. The output of each tree is combined with the others through a reduction operation, such as a majority vote or a summation, followed by an activation function. Modern tree-based models consist of millions of nodes and thousands of trees that need to be run in parallel. Such large ensembles are not suitable for being accelerated with traditional hardware, such as CPUs and GPUs, due to the highly irregular memory access and unpredictable traverse time, given the presence of short and tall tree branches.^247^

**Figure 29:**
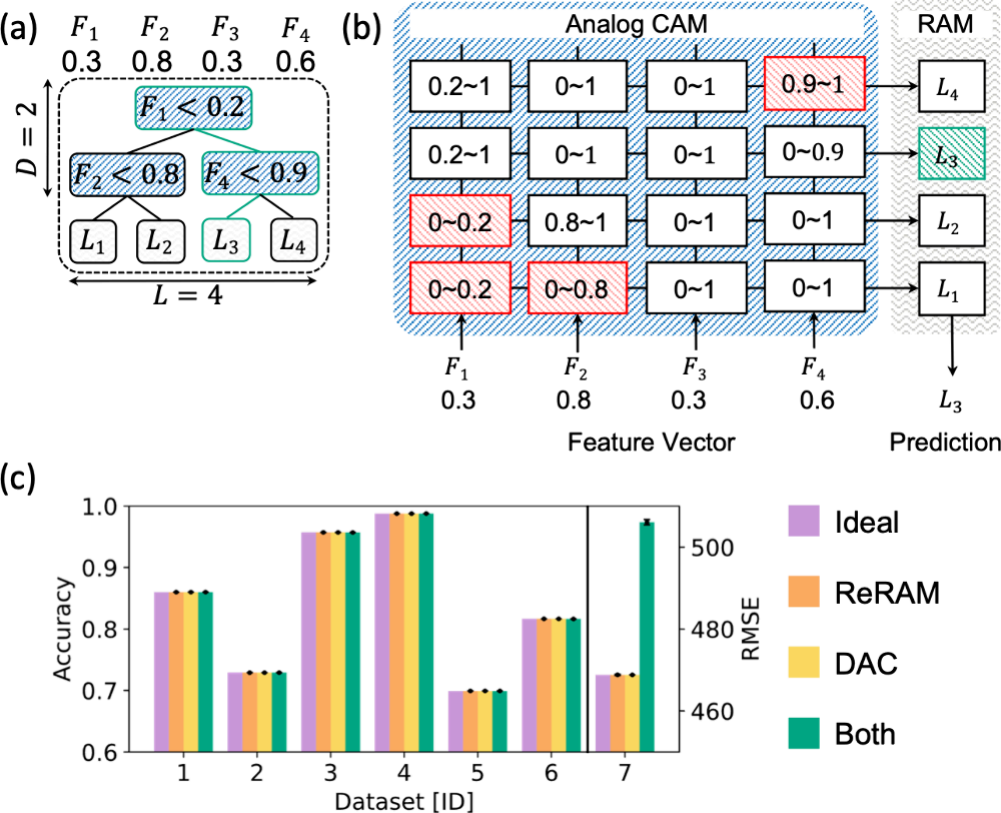
Illustration of Analog CAM-based decision tree inference. (a) Example of decision tree and (b) its mapping into the analog CAM. (c) Accuracy for multiple data sets considering ideal and noisy devices/circuits. Adapted from ref 211. Copyright 2023 IEEE with Creative Commons Attribution 4.0 license https://creativecommons.org/licenses/by-nc-nd/4.0/.

Figure 29b shows the mapping of the decision tree in Figure 29a into an analog CAM. Root-to-leaf branches are mapped in the analog CAM rows in which each cell is performing the comparison corresponding to a node. The feature vector is applied at the input, resulting in a match for the predicted class, which can be retrieved in the adjacent memory. Large-scale simulations and benchmarks have shown tremendous improvement in the throughput and latency compared with other technologies,^211^ with up to 10,000× shorter latency. Such a large improvement is because the performance is essentially independent of the model size given the massively parallel inference operation. Interestingly, thanks to their ensembling behavior, tree-based models do not require high precision of the memory cell, given that even if an error is committed in one tree, it might be recovered by another in the ensemble. Figure 29c shows the accuracy for multiple data sets inference using ideal devices and circuits, injecting RRAM and DAC noise. Results demonstrate only a small impact in the case of regression problems, in which the Root Mean Square Error (RMSE) is considered, and no statistically significant degradation in the case of classification problems.

Both IMVM and analog CAM circuits rely on the multiplication taking place in the circuit as *I* = *GV*, thus requiring that the memory element in the CBA displays a highly linear, or ohmic, *I*–*V* curve. While in CBAs for MVM, typically inputs are applied digitally to the WL, and analog inputs are applied directly to the TE in the case of IMVM. Similarly in analog CAM, the voltage divider between the input transistor, which is activated with analog voltages representing the input, and the RRAM device effectively induces a different analog voltage applied to the RRAM device. However, RRAM devices generally display nonlinear *I*–*V* characteristics, which are correlated to the density of oxygen vacancies in the CF.^248^ Nonlinearity effects can be minimized by operating the device in a limited number of states, typically close to the LRS, in which the conduction appears more linear. In that case, simulations have shown a uniform CF independent of the CC, which allows better heat dissipation. However, this approach may significantly limit the available stable levels in the cell.

The RRAM device stack can be optimized to offer a better linearity. For example, the TE, or oxygen exchange layer, can affect oxygen availability during forming and switching, thus affecting the shape and uniformity of the CF and ultimately controlling the conduction linearity. Figure 30a shows the measured and simulated *I*–*V* characteristics for multiple CCs of a RRAM device with a Ti/Ta_2_O_5–*x*_ stack.^249^ The curves indicate a significant nonlinearity at low CCs, which are attributed by simulations to the conical shape of the CF and the limited thermal conductivity of the switching material. Figure 30b shows the effect of replacing Ti with Ta as the TE material, resulting in a Ta/Ta_2_O_5–*x*_ stack and largely contributing to improving the linearity of the *I*–*V* curve. Figure 30c shows a comparison between the Ti/Ta_2_O_5–*x*_ and Ta/Ta_2_O_5–*x*_ through the CDF of the nonlinearity parameter, defined as *I*(*V*_*read*_)/*I*(0.5*V*_*read*_), where *V*_*read*_ is the read voltage, after programming multiple devices for multiple cycles with *I*_*C*_ = 100 μA The results suggest a nonlinearity of about 2, which corresponds to a perfectly ohmic behavior, for the quasi-metallic filament of the Ta-based RRAM, compared to a large spread of nonlinearity parameters with a median along 10 for the Ti-based RRAM.^249^

**Figure 30:**
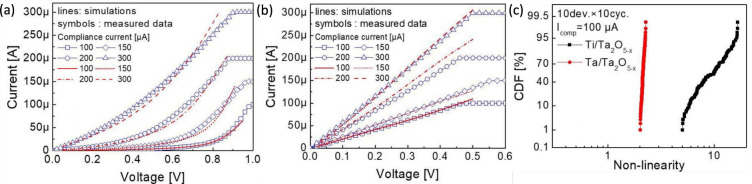
Linearity of the conduction characteristics of RRAM devices. (a) Measured and simulated *I*–*V* characteristics of Ti/Ta_2_O_5–*x*_. (b) Same except for Ta/Ta_2_O_5–*x*_. (c) CDF of the nonlinearity for the two stacks, including both device-to-device and cycle-to-cycle variability. Adapted with permission from ref 248. Copyright 2019 IEEE.

### 5.5 Combinatorial Optimization

Optimization problems, such as Boolean satisfiability, are at the core of many scientific, security, and machine-learning problems.^250^ Typically, optimization consists of finding a set of input values that satisfy a certain number of conditions, e.g. clauses. There are two classes of algorithms for solving optimization problems, namely exact and stochastic solvers. Exact solvers always lead to a solution, although they tend to be slow for classes of problems in which the structure is unknown or difficult to grasp. On the opposite side, stochastic solvers based, for example, on quadratic unconstrained binary optimization (QUBO) with simulated annealing (SA)^251^ can efficiently solve problems with random structures. Interestingly, such workloads can be mapped in computing primitives such as the Ising machine or the Hopfield neural network (HNN).^252^ The typical HNN operates in a recurrent, iterative mode, where, at each iteration, given a vector of *spins s* as input, the network computes the output *v*_*i*_ defined as

25 $$v_{i}=\{\begin{array}{ll} +1, & \mathrm{if}\;u_{i}\geq \theta_{i}\\ -1, & \mathrm{otherwise} \end{array}$$

where *u*_*i*_ is the MVM operation, namely:

26 $$u_{i}=\underset{j\neq i}{\sum }W_{ij}v_{j}$$

where *W*_*ij*_ is a coupling matrix, representing the specific problem to be solved,^250^ while *θ*_*i*_ represents a threshold. The HNN iteratively converges toward the minimum of its energy function given by

27 $$E=-\frac{1}{2}\underset{i,j}{\sum }W_{ij}v_{i}v_{j}+\underset{i}{\sum }\theta_{i}v_{i}$$

However, depending on the initial conditions and energy landscape defined by the coupling matrix *W*_*ij*_, the HNN can remain stuck at a local minimum. To prevent getting stuck at the local minima, noise can be added to *u*_*i*_, which is thus equivalent to implementing an SA algorithm.^153^

The main computational complexity of the HNN consists of the MVM for computing *u* in eq 26; thus, RRAM CBAs offer an efficient implementation of HNNs and Ising machines.^153,154,253,254^ Figure 31a shows the circuit schematic of an optimization solver based on an RRAM CBA.^253^ The circuit is very similar to a recurrent version of the DPE in Section 4.1, where the output is directly fed back to the input without any postprocessing. Also, input and output signals are digital according to eq 25, which is implemented by comparators in the circuit of Figure 31a. Note that the HNN circuit generally does not need power-hungry ADCs or any complicated digital periphery between inputs and outputs, thus making the circuit particularly energy efficient. Finally, the intrinsic noise of RRAM devices can be leveraged for performing a variety of annealing techniques.

**Figure 31:**
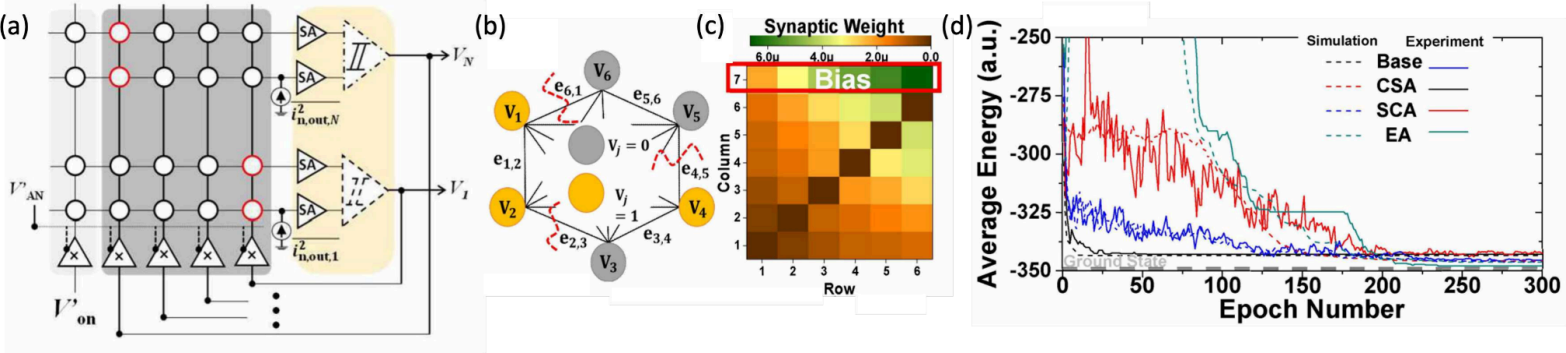
RRAM-based solution of combinatorial optimization problems. (a) Hopfield neural network (HNN). (b) Example of a 6-node weighted graph partitioning problem and (c) its corresponding synaptic weights mapped into a RRAM CBA. (d) Energy as a function of the number of epochs for multiple annealing schemes. Adapted with permission from ref 250. Copyright 2019 IEEE.

Figure 31b shows an example of a weighted graph partitioning problem that involves dividing the vertices of a graph into disjointed subsets while optimizing a specific criterion. Figure 31c shows the mapping of the graph partitioning problem into a CBA.^253^ In this example, multiple intrinsic annealing techniques, such as chaotic simulated annealing (CSA), stochastic simulated annealing (SSA), and exponential annealing (EA), have been tested and compared in terms of the performance and efficiency of the solution. Figure 31c shows the energy function during the circuit iterations, indicating different convergence speeds for the various annealing schemes. The annealing scheme can be controlled by effectively programming the CBA to inject random currents having different types of chaotic behavior. While HNNs can efficiently solve QUBO problems, practical industrially relevant optimization problems, such as Boolean satisfiability problems (SAT), show a higher number (i.e., >2) of interactions. Mapping SAT or in general polynomial unconstrained binary optimization (PUBO) can lead to a significant overhead, due to auxiliary variables making the problem more complex.^255^ Recently, higher-order optimization solvers based on RRAM have also been presented.^256,257^

### 5.6 Stochastic Computing

While noise can be useful for SA and other optimization metaheuristics, it can also be used as a source of entropy or seed for stochastic computing. For example, in the case of Bayesian approaches, noise can be used in different ways; namely, it can be (i) tolerated, such as in the case of Bayesian machines, (ii) embraced, for example during inference Bayesian neural networks, or (iii) exploited for Bayesian learning.^258^

Bayesian machines can be implemented in near-memory accelerators able to perform efficient Bayesian inference, namely, generating a posterior distribution *p*(*Y* = *y*|*O*_*i*_) based on the prior distribution *p*(*Y* = *y*) and observations *O*_*i*_. Referring to the simple case of conditionally independent processes, the Bayesian inference consists of computing:

28 $$p\left(Y=y|O_{i}\right)=p\left(Y=y\right)\underset{i}{\prod }p\left(O_{i}|Y=y\right)$$

A RRAM-based Bayesian machine^259^ implements eq 28 by encoding each likelihood factor, i.e. *p*(*O*_*i*_|*Y*_*m*_ = *y*_*m*_), in an independent RRAM array and performing multiplication, which in the case of stochastic computing consists of just an AND operation between stochastic streams of random bits, with a multiplier tree close to the memories. The observations *O*_*i*_ are used as addresses for the memory arrays, selecting the corresponding likelihood value. Being that the operation is inherently stochastic and since the RRAM devices are programmed in binary states, RRAM-based Bayesian machines are highly resilient to noise and variations.

In Bayesian neural networks, the model weights are probability distributions that are sampled during inference; thus, noisy RRAM devices can naturally represent the network weights.^260^ The noise of RRAM devices in the LRS generally displays a normal probability distribution, where its standard deviation is tightly related to the mean value.^261^ Thus, by considering this relationship during training and without using the standard deviation as a free parameter, it is possible to implement the distribution of a model parameter with one or more RRAM devices. A Bayesian neural network layer can thus be mapped similarly to a conventional DNN, as shown in Section 4.1, where each computational layer consists of a dot product operation. Interestingly, Bayesian neural networks can provide a distribution of the outputs, leading to important insights for model explanation.

Finally, the stochastic properties of RRAM devices can be leveraged for Bayesian learning, for example in the case of training Metropolis-Hasting Monte Carlo Markov Chain (MCMC) models.^261^ Weights are sampled randomly from a Gaussian distribution whose mean was learned at the previous learning iteration step. Sampling can thus be directly obtained from RRAMs in LRS. The technique was used to train a Bayesian neural network with software equivalent accuracy.^261^

### 5.7 Neuromorphic Computing

Neuromorphic computing refers to the ability of electronic circuits to emulate specific mechanisms of information processing taking place in the brain.^262^ The human brain is characterized by extremely low energy consumption in the range of 20 W, combined with high parallelism and a unique capability to adapt to the environment and learn from external stimulation. Compared to artificial computers based on the von Neumann architecture, where memory and processing functions take place in different modules within the computing system, the human brain is characterized by the memory and processing being colocated within the same biological network. Such *in situ* processing of information within memory has been a constant inspiration for the IMC field to maximize energy efficiency by minimizing data movement.^263^ Neuromorphic engineering and computing were introduced in the early 1990s and revived in the last 20 years as a response to the fast growth of interest in AI and the emergence of the hardware limitations to solve AI tasks.^264,265^

A specific challenge of neuromorphic computing is the misalignment between the brain processes, including fundamental physiological, biochemical, and biophysical processes, and the conventional CMOS technology, including transistors, resistors, capacitors, and their constitutive electrical characteristics. For instance, the input/output characteristics, localization, connectivity, and time scales are different in the human brain and in a digital or analog computer. To fill this gap, it is essential to introduce a new technology platform of neuromorphic devices that can provide a realistic equivalent of the bioneurological processes within electronic hardware.^5,266,267^ From this standpoint, RRAM devices have been the object of growing interest as a memristive technology capable of providing unique properties of dynamic and static learning.^268–270^ In fact, RRAM and other emerging NVMs can provide a wide portfolio of device physical properties capable of mimicking the most important neurobiological processes occurring at the neuron soma, synapses and dendrites.^267^

Figure 32 shows an overview of the major neurobiological processing mechanisms and their possible emulation via emerging NVM such as RRAM and PCM.^267,271^ Spikes are delivered from one neuron to the others to exchange information, inducing synaptic excitatory/inhibitory adaptation and dendritic processing. The summation of weighted spikes can be mimicked by the MVM operation taking place in RRAM CBAs, where input voltage signals are converted into currents by Ohm’s law, followed by current summation and collection by Kirchhoff’s law (see Section 4.1).^4,118,170^ Leaky integrate-and-fire (LIF) neuron dynamics can be emulated by pulse accumulation in various types of multilevel NVMs, such as PCM^158,272^ and RRAM.^273,274^ Other neuron mechanisms, such as stochastic spiking^275^ and homeostasis,^276^ have also been implemented in RRAM devices and circuits. Dendritic filtering has been reported via the use of volatile RRAM devices based on TaO_*x*_/AlO_δ_ for the artificial dendrite and on NbO_*x*_ for the artificial soma.^277^ Synaptic elements based on RRAM were demonstrated to display both long-term potentiation (LTP)^146,161,278,279^ and short-term potentiation (STP).^22,280^

**Figure 32:**
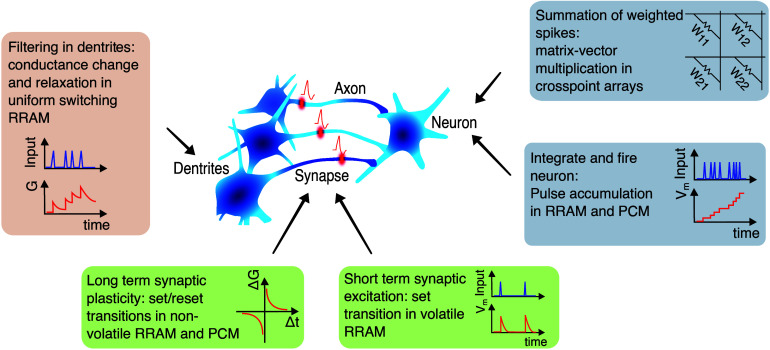
Brain-inspired computing with RRAM device physics, including neuron summation, integration and fire, dendritic processing, and synaptic long- and short-term plasticity. Adapted with permission from ref 267. Copyright 2021 AIP Publishing LLC.

Neuromorphic computing aims at implementing a specific brain-inspired function, such as unsupervised learning, spatiotemporal pattern recognition, or in-sensor computing, via a specific combination of CMOS and RRAM circuits. Within this combination, the RRAM technology can provide a unique asset of device properties that would be impossible to achieve with CMOS-only devices. For instance, RRAM can provide long-term plasticity via the nonvolatile storage of a parameter, such as a weight or a membrane potential. Such a nonvolatile effect is essential for reproducing learning and adaptation in the human brain, thus motivating the need for RRAM technology for neuromorphic computing. Similarly, working memory for short-term plasticity in speech recognition and decision-making is characterized by a typically long time scale.^281^ These long-time constants require relatively large capacitors within a pure-CMOS circuit, which would negatively impact the cost of the neuromorphic chip. Finally, RRAM devices can provide multilevel storage of parameters with high density, which ensures high connectivity of the human brain, in the range of 10^4^ synaptic connections per neuron.

Neuromorphic circuits displaying adaptive, unsupervised learning where RRAM devices play the role of synaptic elements sensitive to spikes have attracted strong attention. LTP in the human brain has been traditionally attributed to Hebbian learning, where synapses selectively display potentiation or depression depending on when they are subject to a large spiking activity or to a strong interaction of more spikes in time.^282^ Examples of Hebbian learning include paired-pulse facilitation (PPF),^94,283^ spike-timing dependent plasticity (STDP),^269,270,278,284^ triplet-based LTP^285,286^ and spike-rate dependent plasticity (SRDP) according to the Bienenstock–Cooper–Munro (BCM) rule.^287–289^

One of the most popular descriptions of Hebbian learning in the brain is STDP, where the synaptic potentiation or depression is the result of the occurrence of a pair of spikes originating at the presynaptic and postsynaptic neurons.^269,270,278,284^ In particular, according to the STDP rule, LTP takes place when the postsynaptic spike follows the presynaptic one, namely when the spiking delay time Δ*t* is positive, or Δ*t* = *t*_*post*_ – *t*_*pre*_ > 0, where *t*_*post*_ and *t*_*pre*_ are the postsynaptic and presynaptic time, respectively. On the other hand, depression takes place when the delay time is negative, namely Δ*t* < 0. STDP can be achieved by careful engineering of the post-/presynaptic spikes so that the overlap between the spikes results in a pulse across the RRAM devices with a pulse width and/or amplitude that depends on the sign and magnitude of Δ*t*.^161,290,291^ Note that the pulses resulting from the spike overlap must fall in the programming regime in Figure 16, thus resulting in dynamic IMC that is necessary for permanent modification of the RRAM conductance.

Figure 33a shows the schematic circuit for an RRAM synapse with 1T1R structure capable of LTP via STDP.^279^ The presynaptic neuron drives the gate terminal of the 1T1R synapse, while the output current is injected to the postsynaptic neuron via the source terminal, ideally serving as a virtual ground terminal. When the postsynaptic neuron fires, a spike is applied to the TE terminal of the synapse, normally biased to a read voltage to sustain the synaptic readout current. The postsynaptic spike displays a positive pulse followed by a negative pulse exceeding the set and reset voltages, respectively, as shown in Figure 33b. Due to the particular shape of the postsynaptic spike, the overlap between pre- and postsynaptic spikes causes LTP and LTD for Δ*t* > 0 and Δ*t* < 0, respectively.^279^

**Figure 33:**
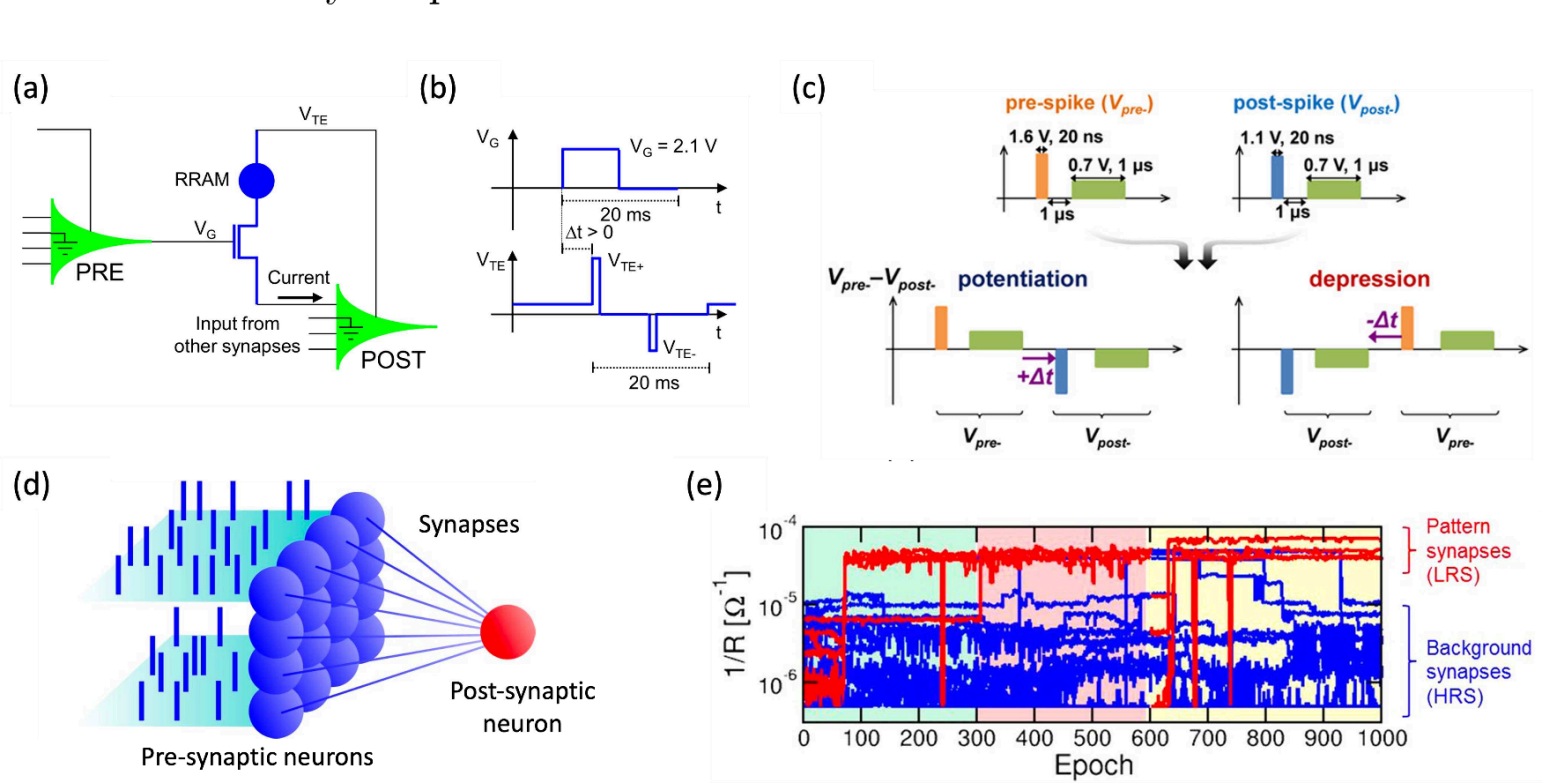
Long-term potentiation (LTP) with RRAM devices. (a) Structure of a 1T1R artificial synapse for STDP.^279^ (b) Sketch of the overlapping presynaptic (gate) spike and postsynaptic (TE) spike for the case of synaptic potentiation (Δ*t* > 0).^279^ (c) Sketch of STDP for second-order RRAM device where nonoverlapping spikes can lead to LTP or Ltd.^292^ (d) Single-layer perceptron (SLP) with STDP synapses capable of unsupervised learning of spatial visual patterns.^158^ (e) Measured synaptic conductance as a function of the number of epochs, namely the spike number, indicating that LTP and LTD take place in specific synaptic elements in (d) depending on their position with respect to the stimulating pattern.^162^ Panels (a,b) are adapted with permission from ref 279. Copyright 2016 IEEE. Panel (c) is adapted with permission from ref 292. Copyright 2015 ACS Publications. Panels (d,e) are adapted from ref 162. Copyright 2017 Nature Publishing Group with Creative Commons Attribution 4.0 International License https://creativecommons.org/licenses/by/4.0/.

The overlap concept for STDP can require relatively long pulses in the same range as the typical time delay in the STDP characteristic that must be implemented. Long pulse widths can also result in large energy consumption or occupation of shared interconnect lines for relatively long times, preventing massive parallelism of spike communication. These problems can be overcome by the nonoverlap STDP algorithm in Figure 33c, where the simple sequence of postsynaptic and postsynaptic spikes without overlap can result in delay-dependent LTP.^292^ This is possible in the so-called second-order memristor, consisting of a bilayer stack Ta_2_O_5–*x*_/TaO_*y*_, where PPF due to thermal or chemical interaction between successive pulses can lead to STDP with nonoverlapping spikes.^292,293^

STDP provides the basis for unsupervised learning within the single-layer perceptron (SLP) circuit sketched in Figure 33d, where *N* input neurons are connected to a single output neuron via *N* synapses. The output neuron is set to operate according to an integrate-and-fire mode, where input spikes cause an increase in the local membrane potential *V*_*m*_, until *V*_*m*_ reaches the threshold for fire. The application of an input spiking stimulation, such as a visual pattern, leads to selective LTP and LTD depending on the delay time Δ*t*. Due to the correlation between input pattern spikes, the presentation of the input pattern leads to output neuron fire with Δ*t* > 0, thus causing LTP. On the other hand, uncorrelated input noise spikes cause a fire with Δ*t* < 0, thus causing Ltd.^158,162^ Figure 33e shows the experimental evolution of the conductance within the SLP, indicating selective LTP and LTD taking place in synapses stimulated by the input pattern and by noise, respectively, as a result of the STDP in 1T1R synapses based on RRAM.^162^ The development of such multisynaptic circuits is extremely promising for developing perceptron-like networks capable of autonomous learning and adaptation.

In addition to LTP, neuromorphic computing takes advantage of short-term memory, which supports several functions in the human brain, such as speech/language understanding, problem-solving, decision-making, navigation, selective attention, mental arithmetic, and so on. Short-term memory can be implemented in RRAM devices by engineering the stack to achieve volatile switching, as opposed to the nonvolatile memory effect that is generally pursued for memory applications. Figure 34a shows the measured *I*–*V* curve for a volatile RRAM device based on Ag nanodots, where the Ag filament is not stable after switching, thus collapsing the device to an off state.^294^ Set transition is observed under both positive and negative voltages; however, the device switches back to the off state as the voltage is decreased below a minimum holding voltage *V*_*hold*_.^294–296^ To better highlight the time scale of the on–off transition, Figure 34b shows the measured applied voltage and the current response as a function of time.^295^ After the applied spike is removed, the current response remains active under a small applied read voltage for a retention time on the order of 150 μs.^295^ The limited retention time can be attributed to the unstable Ag filament, where the tendency to minimize the free energy associated with the surface leads to the collapse of the filament toward the electrodes, or its breakdown into smaller nanocrystals.^94,110^

**Figure 34:**
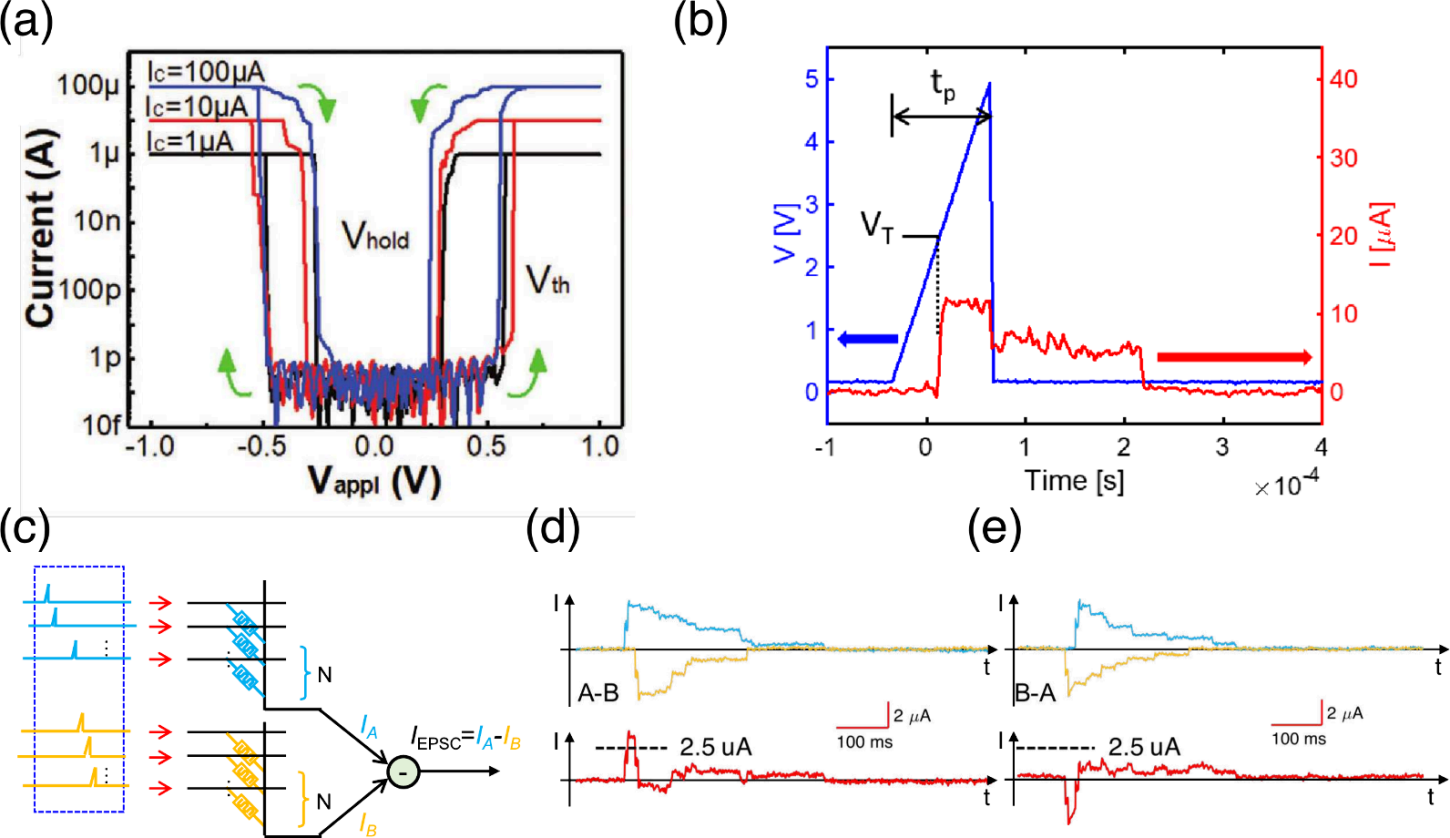
Neuromorphic computing with volatile RRAM devices with short-term memory. (a) *I*–*V* characteristics of an RRAM device with Ag nanocrystals displaying volatile switching to the LRS under both positive and negative voltage sweep.^294^ (b) Measured applied voltage and current response for an Ag-based volatile RRAM device, highlighting the retention time in the range of about 150 μs.^295^ (c) Spatiotemporal pattern recognition based on a differential circuit comparing the current response of excitatory and inhibitory synapses.^22^ (d) Measured excitatory postsynaptic current (EPSC) for a sequence A–B in (c), resulting in a positive EPSC.^22^ (e) Same as (d) but for sequence B–A, resulting in a negative EPSC.^22^ Panel (a) is adapted with permission from ref 294. Copyright 2020 Wiley VCH. Panel (b) is adapted with permission from ref 295. Copyright 2021 IEEE. Panels (c, d and e) are adapted from ref 22. Copyright 2021 Wiley VCH with Creative Commons Attribution 4.0 International License https://creativecommons.org/licenses/by/4.0/.

The volatile response of Ag-based RRAM devices can be used for emulating the excitatory postsynaptic current (EPSC) which takes place as a result of the spiking stimulation of a synapse causing the opening of ionic channels for a relatively short period of time.^297^ Figure 34c shows a differential circuit comparing the current response of an excitatory synapse and an inhibitory synapse, each stimulated by pulses of a sequence A-B (preferred) or B-A (nonpreferred).^22^ Each synapse in the figure consists of several RRAM devices, where the composition of several random rectangular responses results in an overall response with an almost exponential decay behavior. As shown in Figure 34d,e, due to the delay between the excitatory and inhibitory synaptic currents, the differential current *I*_*EPSC*_ shows a positive or negative sign for the preferred and nonpreferred sequence, respectively, thus featuring spatiotemporal sequence recognition. Such a short-term EPSC effect enables the recognition of different directions of dynamic visual signals by mimicking the direction-selective ganglion cell in the human retina.^22^

Figure 35a shows a volatile RRAM circuit for the brain-inspired processing of auditory signals. Here, several volatile RRAM devices are stimulated by voltage signals with linearly increasing amplitude close to the threshold voltage *V*_*set*_ for the set transition.^298^ Due to the exponential voltage dependence of switching probability, the number of devices being activated by the input signal increases with the frequency of the signal. This is shown in Figure 35b,c,d, where the application of a train of rectangular pulses shows the activation of an increasing number of devices as the frequency increases from a relatively low value (b) to a relatively high value (d). Note that the number of activated devices can be probed by the current level due to the parallel connection in the circuit of Figure 35a, thus enabling a frequency to current conversion. Figure 35e shows the number *N*_*on*_ of activated devices as a function of frequency, indicating a linear increase of *N*_*on*_ with the logarithmic frequency, as a result of the exponential increase of the switching probability with voltage. These results are compared with the tonotopic characteristic of the cochlea, namely the depth location of stimulated cilia in the cochlea as a function of frequency.^299^ The ability to track the frequency on a logarithmic scale allows for bioinspired tonotopic processing of auditory signals by exploiting the unique exponential voltage response of RRAM switching probability.^298^

**Figure 35:**
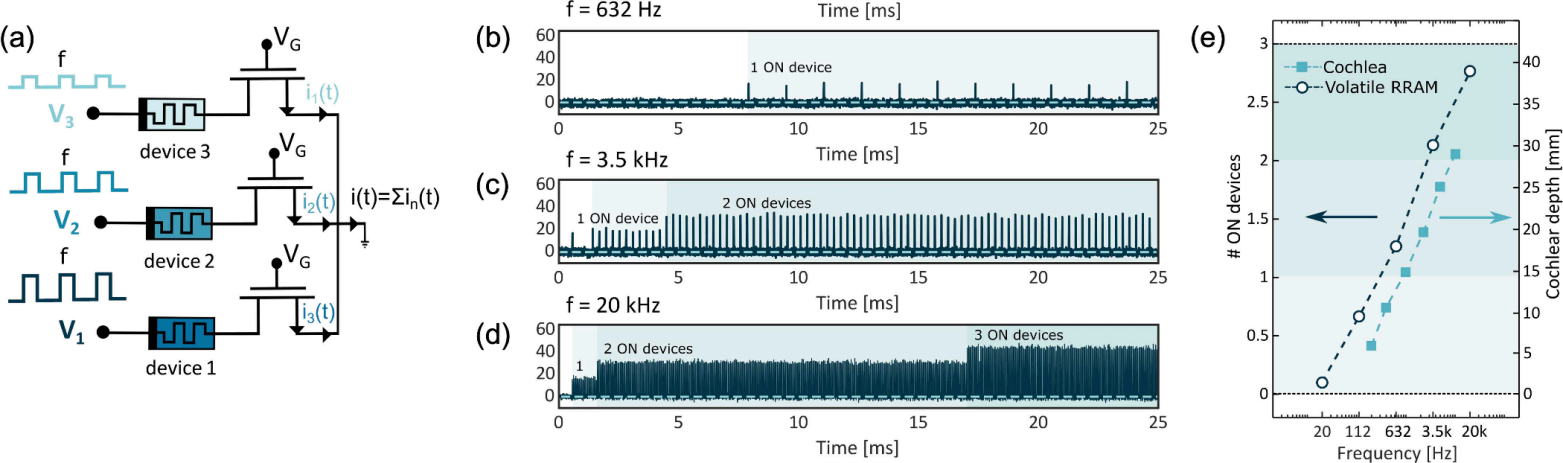
Tonotopic frequency detection in volatile RRAM devices with short-term memory. (a) Circuit for frequency-current conversion by exploiting the exponential dependence of switching probability on voltage.^298^ (b) Response of the circuit to a relatively low stimulating frequency of 632 Hz, resulting in just one device being activated. (c) Same as (b) but for a higher frequency, indicating the activation of 2 devices. (d) Same as (c), but with even higher frequency, causing 3 devices to be activated. (e) Measured number of activated devices as a function of frequency, indicating a linear increase of current on a logarithmic current, due to the exponential voltage dependence of switching probability. Results are compared with the cochlea tonotopic characteristic.^299^ Reproduced from ref 299. Copyright 2024 Nature Publishing Group with Creative Commons Attribution 4.0 International License https://creativecommons.org/licenses/by/4.0/.

An important methodology in neuromorphic computing is *reservoir computing* depicted in Figure 36a, where the input stimuli are processed by a dynamic *reservoir* layer, while a second *readout* layer is used for classification.^300,301^ For the processing of a time-dependent sequence, the reservoir layer must contain dynamic elements, such as LIF neurons or short-term memory devices, with a typical time constant in the same range as the signal being processed. For this purpose, volatile RRAM can provide an ideal technology given the high density and the tunable retention time usually in the range between 1 ms and 1 s.^300^ This is illustrated in Figure 36b, showing the time sequence of input pulses and the corresponding internal state variable, e.g., the conductance of an RRAM device subjected to the input sequence of pulses in the range of the set voltage, thus inducing dynamic short-term potentiation.^300^ The reservoir approach is most suitable for the processing of electro-physiological signals, such as neuron spike sorting,^302^ speech recognition,^303^ and epileptic seizure prediction.^304^ However, not only spatiotemporal patterns but also purely spatial patterns, such as images, can be processed via reservoir computing by suitably converting the spatial pattern into one or more spatiotemporal patterns. This is shown in Figure 36c, where a visual pattern, namely the digit ‘2’ in Figure 36d, is converted into a spatiotemporal pattern consisting of a sequence of 4 pulses across 5 channels, each connected to the input of a RRAM device. The readout current of the RRAM subject to pulse-induced potentiation is then applied to the readout layer for classification. While the reservoir layer is usually untrained, the readout network is a fully connected network where the synaptic weights are pretrained for a specific task, e.g. the classification of digits based on the internal state variables in the reservoir.^300^

**Figure 36:**
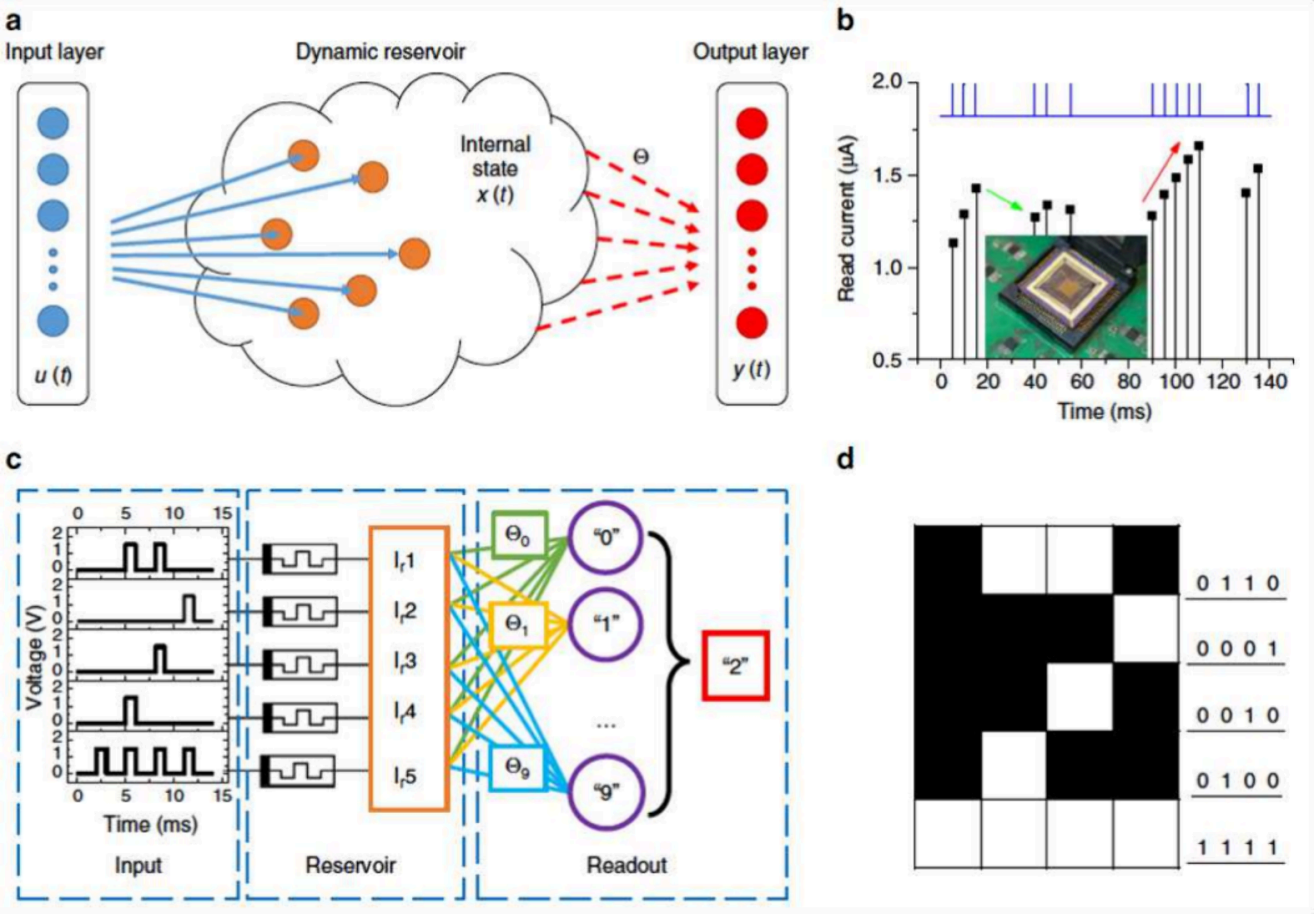
Reservoir computing concept. (a) Sketch of a reservoir computing network, including an input layer for delivering the input signals, a reservoir layer for dynamic processing, and an output layer for classification. (b) Sketch of input signals and internal state variables in the reservoir layer as a function of time. (c) Conversion of a spatial pattern into a spatiotemporal pattern, applied to an RRAM-based reservoir layer and finally classified by a pretrained fully connected read-out layer. (d) Conversion of an image into a spatiotemporal pattern for reservoir computing.^300^ Reproduced with permission from ref 300. Copyright 2017 Nature Publishing Group.

Similar reservoir computing demonstrations have been reported for other memory devices, such as MoS_2_-based charge-trap memory devices^88^ and spin-torque nano-oscillators.^305^ The dynamic layer in reservoir computing does not necessarily consist of a well-defined, top-down structured memory array; rather, it can also feature a random, bottom-up nanostructure, such as a network of nanowires^306^ or nanotubes.^307^ This is the so-called in-materia computing approach, where the reservoir layer consists of a material or structure where input signals are applied, while output signals are extracted to monitor the internal state variable, usually consisting of a localized chemical, physical or electrical property of the material.^306,308,309^ Such in-materia computing is particularly promising given the extreme scalability, BEOL integration, and dynamic response with the same time scale of the electro-physiological signals that need to be processed in the neuromorphic system.

While STM is an essential requirement for neuromorphic computing, the typical decay time constant of STM should match the time scale of the signal to be processed. For instance, speech recognition via reservoir computing requires that the memory devices in the reservoir layer react to signal stimulations within the frequency range (from 0.1 to 20 kHz) of the audio signal to be processed.^298,303^ Similarly, neuron spike sorting and classification requires that memory devices display an STM decay in the time scale between a few milliseconds to a few seconds.^302^ The STM time constant should also be statistically uniform for the same device operated in several cycles and for different devices within the same or different circuits, thus enabling the design of SNN circuits with predictable behavior.

To assess the time constant of the STM, RRAM devices are measured according to the experimental technique shown in Figure 37a. Here, a voltage pulse is applied to the RRAM device to induce the set transition; then the voltage is rapidly reduced to a relatively low value *V*_*read*_ to enable readout without disturbing the device.^110^ The device, consisting of a MIM structure with Au electrodes and a dielectric layer of silk-Ag nanowires composite, was monitored by measuring the voltage across a low series resistance, typically of 50 Ω, by an oscilloscope (see inset). Figure 37b shows the RRAM conductance, measured as the response current divided by *V*_*read*_, for increasing *I*_*C*_.^110^ The conductance sharply drops after a variable retention time *t*_*R*_. As *I*_*C*_ increases, both *G* and *t*_*R*_ increase as a result of the larger cross-sectional area of the CF, thus resulting in a larger conductance and better stability with a longer retention time. The CF decay is attributed to the structural instability of the Ag CF, where the relatively large surface energy causes a spontaneous surface rediffusion of Ag atoms to minimize the surface-to-volume ratio.^110^

**Figure 37:**
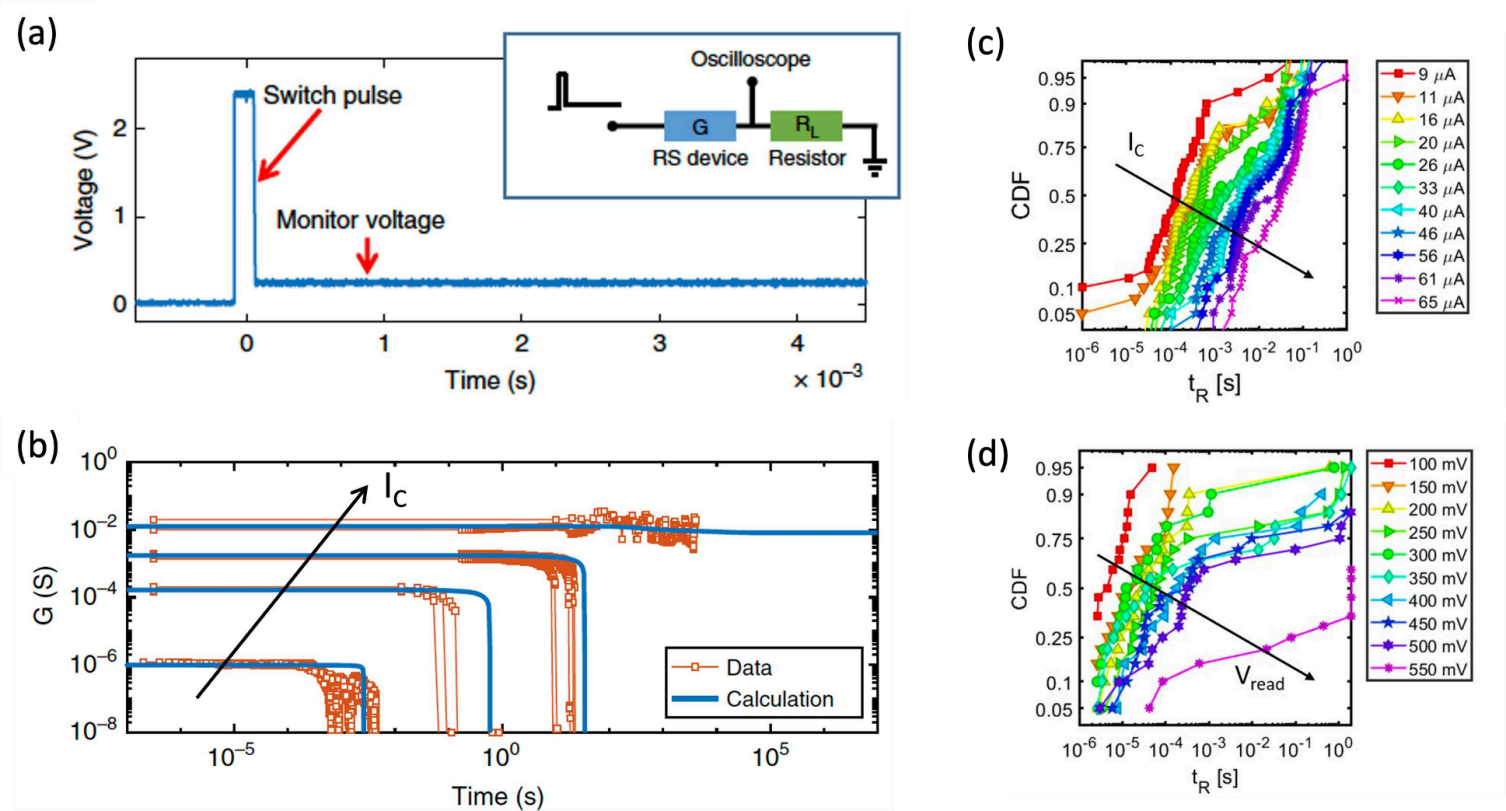
Characterization of STM behavior and time scale in volatile RRAM devices. (a) Voltage waveform for the characterization of the short-term memory effect in RRAM. The inset shows the electrical circuit connection to probe the current response of the RS element via a load resistor. (b) Measured conductance for RRAM devices at increasing *I*_*C*_. (c) Cumulative distributions of *t*_*T*_ for increasing *I*_*C*_. (d) Cumulative distributions of *t*_*R*_ for increasing *V*_*read*_. The STM time constant can be tuned by *I*_*C*_ and *V*_*read*_, thus enabling reservoir computing with dynamic RRAM for various real-life applications. Panels (a,b) are adapted from ref 110. Copyright 2019 Nature Publishing Group with Creative Commons Attribution 4.0 International License https://creativecommons.org/licenses/by/4.0/. Panels (c,d) are adapted with permission from ref 295. Copyright 2021 IEEE.

Figure 37c shows the cumulative distributions of measured *t*_*R*_ on a single RRAM with a Ag/SiO_2_/C stack.^295^ Data confirm the increase of *t*_*R*_ with increasing *I*_*C*_, as a result of the improved stability with increasing size of the CF. Data also show that the *t*_*R*_ scale can be tuned between approximately 0.1 and 50 ms by operating the device at different *I*_*C*_. This property is essential to enable neuromorphic applications for the processing of real-life data such as electrophysiological signals, speech and gesture, which generally display characteristic time and frequency over different time scales. Figure 37d shows the cumulative distributions of *t*_*R*_ for increasing *V*_*read*_, where *t*_*R*_ increases with *V*_*read*_ due to the competition between field-induced ionic drift, sustaining the CF structural stability, and ionic rediffusion responsible for CF dissolution.^295^

The results in Figure 37 support the ability to tune the retention time, which is useful for applications requiring a large range of decay times for the processing of signals with multiple frequency components. The millisecond time scale of *t*_*R*_ also supports three-factor learning, where the potentiation and depression of the synaptic RRAM is enabled within a limited time window.^310^ Note that *t*_*R*_ is affected by a non-negligible cycle-to-cycle variation, which might be due to the stochastic shape and size of the CF, as well as the stochastic rediffusion processes, which might be sensitive to the local microstructure of defects and grain boundaries. Such a stochastic variation can impact the accuracy of a reservoir computing system for signal recognition. These variations can be mitigated by circuit design solutions, such as the use of several RRAM devices in parallel where the retention time is dictated by the decay of the summation of the RRAM currents.^311^

## 6 Conclusions

This paper reviews the main applications and corresponding requirements for IMC with RRAM devices, as summarized in Table 2. RRAM development and understanding have made significant progress during the last 20 years. Thanks to the extensive efforts in device engineering, characterization, and modeling, RRAM is today a consolidated technology that has been extensively demonstrated in CMOS-compatible chips, for both stand-alone and embedded NVM, as well as for a number of technological demonstrations of IMC. RRAM appears as a unique technology capable of offering significant added value, in terms of nonvolatile storage, low-voltage/low-current operation, compatibility with the CMOS process flow, scalability, multilevel operation, and fast read. At the same time, to face the competition of conventional CMOS memories, such as DRAM and SRAM, and of other emerging NVM technologies, more work is needed to address some remaining challenges.

In particular, IMC poses significant challenges to RRAM given the diverse requirements for various computing algorithms. For instance, a key RRAM limitation for DNN inference accelerators is the stochastic variation and fluctuation of resistance, which affects the weight bit precision and the accuracy of IMC. More challenges arise with the inference of modern models, such as transformers where the attention mechanism plays a pivotal role. The attention matrices change for each input sequence; thus, CBAs have to be rewritten, making endurance an extra requirement. Endurance is also an essential requirement for DNN training due to the extensive weight update in the hardware back-propagation algorithm. DNN training also requires RRAM devices with highly linear weight updates to implement the back-propagation without significantly changing the learning rule. For inverse linear algebra operations such as the solution of linear equations, linear regression, and PCA, the main requirements are high endurance, to enable rapid and frequent reconfiguration, and highly linear *I*–*V* characteristics, in addition to high multilevel precision of the mapped weight with low variation and noise. Similar properties are needed for the implementation of optimization solvers accelerated with RRAM-based Ising machines and HNN. Note that high endurance and high retention are generally not required at the same time, since applications requiring fast/frequent reconfiguration, such as DNN training, generally do not require the persistency of the mapped parameters for a long time and high temperature.

In the case of stochastic computing, such as Bayesian learning, noise must be minimized to guarantee sampling without drift of the mean or standard deviation, whereas retention is a strong requirement. Tree-based ML circuits, specifically those relying on analog CAMs, require multilevel operation to represent different conditional branching without significant overhead in the circuit peripherals. In particular, compared with CBAs in which multiple devices can be used in parallel, slicing schemes in CAMs can result in exponential overhead. Linear *I*–*V* curves are also essential requirements for analog CAM. Finally, in the case of SNN, STM and good endurance are a strong requirement for implementing reservoir computing and neuromorphic learning and adaptation.

In conclusion, diverse RRAM requirements arise for diverse applications. Careful RRAM engineering is needed to meet these multiple requirements as much as possible within a single device technology. Alternatively, material stacks and algorithm optimization could be developed to match the specific requirements for any individual application, with the aid of specialized peripheral circuits and architecture improvements that can be used to mitigate RRAM nonidealities and assist the specialized RRAM algorithms, such as advanced PV methods. Hardware-software and device-to-system co-optimization routines tailored for the various applications, supported by novel automated design techniques, might enable RRAM technology for energy-efficient, high-performance, accurate IMC.

## List of Acronyms

1C: one-capacitor
1R: one-resistor
1S1R: one-selector/one-resistor
1T1R: one-transistor/one-resistor
A-ECC: analog error correction code
ADC: analog-digital converter
AI: artificial intelligence
ALD: atomic layer deposition
BCM: Bienenstock–Cooper–Munro
BEOL: back-end of the line
BL: bitline
CAM: content addressable memory
CBA: crossbar array
CBRAM: conductive-bridge random-access memory
CC: compliance current
CDF: cumulative distribution function
CF: conductive filament
CIFAR-10: Canadian Institute for Advanced Research with 10 classes
CMOS: complementary metal-oxide-semiconductor
CPU: central processing unit
CSA: chaotic simulated annealing
CTM: charge trap memory
CVD: chemical vapor deposition
DAC: digital-analog converter
DL: Data line
DNN: Deep neural network
DPE: dot-product engine
DRAM: dynamic random access memory
DTCO: design/technology co-optimization
EA: exponential annealing
ECC: error correction code
eNVM: embedded nonvolatile memory
EPSC: excitatory postsynaptic current
ECRAM: electrochemical random access memory
FeRAM: ferroelectric random access memory
FET: field-effect transistor
FP64: floating point 64-bit
FSR: full-scale range
GPU: graphical processing unit
HKMG: high-k/metal-gate
HNN: Hopfield Neural Network
HRS: high resistance state
I–V: current–voltage
IGVVA: incremental gate voltage with verify algorithm
IMC: in-memory computing
IMVM: inverse matrix vector multiplication
ISPVA: incremental step pulse with verify algorithm
LIF: leaky integrate-and-fire
LLM: large language model
LRS: low resistance state
LSE: least-squares error
LTD: long-term depression
LTP: long-term potentiation
MCMC: Monte Carlo Markov Chain
MCU: microcontroller unit
MIM: metal–insulator–metal
ML: match line
MLP: multilayer perceptron
MNIST: Modified National Institute of Standards and Technology
MOCVD: metal-organic chemical vapor deposition
MRAM: magnetic random access memory
MRS: medium resistance state
MVM: matrix vector multiplication
NDR: negative differential resistance
NSR: noise-to-signal ratio
NVM: nonvolatile memory
OEL: oxygen exchange layer
OPA: Outer Product Accumulate
PC: principal component
PCA: principal component analysis
PCM: phase change memory
PPF: paired-pulse facilitation
PUBO: Polynomial Unconstrained Binary Optimization
PUF: physical unclonable function
PV: program verify
QUBO: Quadratic Unconstrained Binary Optimization
RAM: random access memory
ReLU: rectifying linear unit
RMSE: root mean square error
RRAM: resistive switching random-access memory
RS: resistive switching
RTN: random telegraph noise
RW: random walk
SA: simulated annealing
SAT: Boolean satisfiability problem
SCLC: space-charge limited conduction
SCM: storage class memory
SLP: single-layer perceptron
SOM: selector-only memory
SRAM: static random access memory
SRDP: spike-rate dependent plasticity
SSA: stochastic simulated annealing
STDP: spike-timing dependent plasticity
STP: short-term potentiation
TCAM: ternary CAM
TDDB: time-dependent dielectric breakdown
TIA: transimpedance amplifier
TMD: transition metal dichalcogenide
TRNG: true random number generator
vdW: van der Waals
WL: wordline
XPS: X-ray photoelectron spectroscopy
